# Supplementary material for: Efficacy of halopeRIdol to decrease the burden of Delirium In adult Critically ill patiEnts (EuRIDICE): study protocol for a prospective randomised multi-centre double-blind placebo-controlled clinical trial in the Netherlands
Source: BMJ Open. 2020 Sep 23;10(9):e036735. doi: 10.1136/bmjopen-2019-036735 (PMC7513600; doi:10.1136/bmjopen-2019-036735)
Supplement: Supplementary data [file bmjopen-2019-036735supp001.pdf]

Appendix 1: Participating hospitals

Erasmus MC Rotterdam

Albert Schweizer Hospital Dordrecht

Maasstad Hospital Rotterdam

IJsselland Hospital Capelle aan den IJssel

Ikazia Hospital Rotterdam

Franciscus Gasthuis Rotterdam

As of July 2019 two additional ICUs have started recruitment:

Jeroen Bosch Hospital, 's-Hertogenbosch

Radboud University Medical Center, Nijmegen
