# Supplementary material for: Efficacy of halopeRIdol to decrease the burden of Delirium In adult Critically ill patiEnts (EuRIDICE): study protocol for a prospective randomised multi-centre double-blind placebo-controlled clinical trial in the Netherlands
Source: BMJ Open. 2020 Sep 23;10(9):e036735. doi: 10.1136/bmjopen-2019-036735 (PMC7513600; doi:10.1136/bmjopen-2019-036735)
Supplement: Supplementary data [file bmjopen-2019-036735supp002.pdf]

Appendix 2: Economic evaluation

ECONOMIC EVALUATION

General considerations

The primary economic analysis will be a trial-based **cost-utility analysis** from a societal and a healthcare perspective. This analysis will be performed according to the Dutch guidelines (1, 2). The time horizon will be 12 months after randomisation, in order to take all relevant costs and effects regarding the treatment procedure into account. Additionally, a **cost-effectiveness analysis** performed from a societal and health care perspective will be conducted, using delirium-free and coma-free days as outcomes.

If a difference in quality of life is observed at the end of the follow-up period, we will also perform a **model-based extrapolation** of costs and health benefits up to 5 years, exploring the following scenarios: (1) health benefit remain constant after the follow-up period, (2) health benefits are gradually phased out over the course of the modelling time, (3) health benefits are gradually phased out over the course of the modelling time over the first year after follow-up, (4) health benefits abruptly disappear after the follow-up period, but costs remain until the end of the modelling period.

If treatment with haloperidol leads to better health outcomes at higher costs, or if it leads to worse health outcomes and cost savings, incremental cost-utility and incremental cost-effectiveness ratios will be calculated. These express the additional costs per unit of health gain (QALYs, delirium-free days, coma-free days) or the savings per unit of health forgone. The uncertainty around the estimates will be addressed using bootstrapping for the analysis of costs and effects in the first 12 months, and using probabilistic sensitivity analysis in the extrapolation model.

Cost analysis

Healthcare costs will be calculated based on patient-level data on health-care utilization, which will be collected from hospital databases and questionnaires, to be filled out at regular intervals by patients and/or informal caregivers. Cost categories include medication, screenings, inpatient days, contacts with healthcare providers (GP, outpatient visits, and therapists). The questionnaire will also contain questions about absence from paid work by the patient and informal caregivers.

Costs will be calculated by multiplying resource utilization with the cost per unit of resource. Some unit costs will be taken from the 2016 Dutch Manual for Costing Studies(3), but the costs of inpatient days will be assessed following the micro-costing method, which is based on comprehensive ‘bottom-up’ analyses of the activities of staff and other resources that are used

during those days. Medication prices will be based on the official list prices, including value added tax and increased by a standard prescription reimbursement for the pharmacist. The cost of production loss will be calculated according to the Friction Cost Approach.

Patient outcome analysis

The primary outcome measure in the economic evaluation is the difference in QALYs. The secondary effects are the delirium-free and coma-free days after treatment with haloperidol or placebo. As measuring QALYs in adult critically ill patients is not feasible at baseline, it is not possible to estimate the average number of QALYs for each treatment group. However, assuming that there is no difference at randomisation, it is possible to analyse the difference in quality of life at subsequent measurements in a multilevel regression model. This will enable us to calculate a difference in QALYs between the treatment groups over the total follow-up period, using linear intrapolation. HRQoL will be measured on t=1, 3, 6 and 12 months after randomization using the EQ-5D-5L instrument.

References:

1. Hakkaart-van Roijen L., Tan SS., Bouwmans CAM. Handleiding voor kostenonderzoek, methoden en standaard kostprijzen voor economische evaluaties in de gezondheidszorg. College voor zorgverzekeringen, Geactualiseerde versie 2010.
2. Zorginstituut Nederland. Richtlijn voor het uitvoeren van economische evaluaties in de gezondheidszorg. 2015.
3. Hakkaart-van Roijen L., van der Linden N., Bouwmans CAM., Kanters T., Tan SS. Costing manual: Methodology of costing research and reference prices for economic evaluations in healthcare. 2015.
