# Supplementary material for: Efficacy of halopeRIdol to decrease the burden of Delirium In adult Critically ill patiEnts (EuRIDICE): study protocol for a prospective randomised multi-centre double-blind placebo-controlled clinical trial in the Netherlands
Source: BMJ Open. 2020 Sep 23;10(9):e036735. doi: 10.1136/bmjopen-2019-036735 (PMC7513600; doi:10.1136/bmjopen-2019-036735)
Supplement: Supplementary data [file bmjopen-2019-036735supp003.pdf]

Appendix 3: ***Haloperidol SPC***

See this weblink: https://db.cbg-meb.nl/IB-teksten/h03185.pdf
