# Supplementary material for: Efficacy of halopeRIdol to decrease the burden of Delirium In adult Critically ill patiEnts (EuRIDICE): study protocol for a prospective randomised multi-centre double-blind placebo-controlled clinical trial in the Netherlands
Source: BMJ Open. 2020 Sep 23;10(9):e036735. doi: 10.1136/bmjopen-2019-036735 (PMC7513600; doi:10.1136/bmjopen-2019-036735)
Supplement: Supplementary data [file bmjopen-2019-036735supp004.pdf]

Appendix 4: (Semi-) structured review of literature on efficacy and adverse events of haloperidol for delirium in adult critically ill patients

1. Haloperidol as a treatment for ICU delirium

Systematic review of randomised placebo-controlled trials assessing haloperidol for treatment of ICU delirium

Method: A biomedical Information Specialist (BIS) of the Erasmus Medical Center library performed a systematic search aimed at controlled studies on haloperidol for ICU delirium combining the subjects: delirium, ICU and haloperidol, or equivalent terms (see: Appendix for details). No distinction was made in the search between treatment or prevention trials.

Review: Since focus of the EuRIDICE study is on a haloperidol versus placebo comparison, the study selection for this summary is also focused on placebo-controlled haloperidol trials for the treatment of ICU delirium. Systematic reviews from the systematic search are used as a crosscheck to confirm completeness or provide additional insights. The search (total of yielded only 1 study. The MIND trial (2010) was a randomised placebo controlled feasibility, efficacy and safety trial of antipsychotics for ICU delirium in adult mechanically ventilated medical and surgical patients (1). It included three treatment arms (haloperidol, n=35; ziprasidone, n=30 and placebo, n=36) and used a well thought out design (excluding demented patients with a validated tool for cognitive dysfunction, using CAM-ICU as a validated screening tool, a clear protocol with regard to QTc prolongation and study drug dosing, measuring extrapyramidal symptoms with a validated scale and with number of days alive without delirium and coma as the primary outcome (indicating total burden of brain dysfunction, since only assessing delirium days may result in increased coma days and less delirium days being regarded as a – false – improvement). The study used oral haloperidol, no clear sedation protocol aimed at light sedation and crossover antipsychotics were allowed but discouraged. No clear differences were found in the three groups with regard to the primary outcome. Mean haloperidol dose was 15 mg a day but QTc prolongation and extrapyramidal symptoms did not differ between treatment groups. Other medications in this small trial did not differ between groups (propofol, opiates, benzodiazepines). It was concluded that a larger trial would be safe and feasible.

Overview of most recent guidelines' statements on haloperidol as treatment for ICU delirium

Method: Pubmed search on published guidelines including ICU delirium and containing information on

haloperidol. Search terms: guideline, delirium, ICU.

Review: Three recent guidelines were retrieved (2-4). In a Danish guideline (2015) no evidence is stated for pharmacological management(2). A German guideline (2015) advocates symptom-based therapy when delirium screening is positive with haloperidol as a first choice in case of delirium associated with psychotic symptoms only. The “Clinical practice guidelines for the management of pain, agitation, and delirium in adult patients in the intensive care unit” (2013)(3) advocate avoiding ‘antipsychotics’ when risk of torsades de pointes or is present or either baseline QT prolongation or concomitant QT prolonging medication is used. It states that there is no evidence that haloperidol decrease delirium duration, which was perceived as the most relevant issue to address with regard to haloperidol treatment of ICU delirium.

Cochrane review(s)

Method: Search on Cochrane (http://www.cochranelibrary.com) for reviews with search term: ‘delirium’, does not elicit any results pertaining to pharmacological treatment of delirium nor haloperidol.

Review: no Cochrane reviews exist on (ICU) delirium and it’s pharmacological management.

On-going trials

Method: A search for ‘haloperidol’ and ‘delirium’ in the following online trial databases (and including ICU patients); www.trialregister.nl (0 trials); www.clinicaltrials.gov (4 trials).

Review: Four trials were retrieved from www.clinicaltrials.gov. One trial (‘Haloquet’) was not a truly placebo controlled trial because haloperidol was allowed (‘as needed’) in the placebo group and was last updated in 2013 but not published. It consisted of three treatments arms (also quetiapine) and aimed to include a total of 45 patients (and should thus be considered a pilot trial and not an efficacy trial). A second trial enrolled 40 patients and was completed in 2011 but not published. A third trial was a phase-2 safety/efficacy study enrolling 20 patients, last updated in 2007 and not published. The fourth trial (‘The modifying the impact of ICU-associated neurological dysfunction-USA [MIND-USA] study’) is currently recruiting (last verified May 2016 on September 14th). It is a multi-center double blind placebo-controlled trial aiming to enrol 561 patients in three treatment arms: haloperidol, ziprasidone and placebo, by the same research group that did the MIND trial. It includes cognitive and

psychological follow-up at 12 months and is estimated to be completed July 2019. Maximum dose of haloperidol amounts to 10 mg IV q12 hours. Trial design is similar to the EuRIDICE trial, except for the patient experiences and perspective, and the fact that only patients on mechanical ventilation or in shock are included (i.e. the sickest ICU patients). The study protocol has not been published in a peerreviewed journal.

2. Haloperidol to prevent ICU delirium

Systematic review of randomised placebo-controlled trials assessing haloperidol for prevention of ICU delirium; including information from guidelines and Cochrane reviews

Method: A biomedical Information Specialist (BIS) of the Erasmus Medical Center library performed a systematic search aimed at controlled studies on haloperidol for ICU delirium combining the subjects: delirium, ICU and haloperidol, or equivalent terms (see: Appendix for details).

Review: the focus of this section is on randomised placebo-controlled prevention trials of haloperidol for ICU delirium. Three trials were retrieved. One trial included post-operative generally non-critically ill patients (5) and was not further considered for this review. The Hope-ICU trial (2013)(6) was a prophylactic study of haloperidol (2.5mg IV q8h, n=71) versus placebo (n=70) in adult mechanically ventilated ICU patients. The primary end-point of delirium (assessed with CAM-ICU) and coma free days did not differ between groups (5 days in both), but there was a 21% crossover rate with haloperidol in the placebo group. Secondary clinical endpoints such as length of stay at ICU or mortality did not differ but the trial was not powered on these outcomes. Another trial (2016)(7) including mechanically ventilated patients (n=68) with 'subsyndromal' delirium (=an Intensive Care Delirium Screening Checklist [ICDSC] score of 1-3 on a scale of 8, where 4 or more is compatible with delirium) used haloperidol 1mg IV q6h but did not find lower rate of progression to full delirium.

3. Haloperidol: adverse events versus treatment effects in the few available trials

The adverse events associated with haloperidol in the three aforementioned (small) trials (one treatment and two prevention trials) did not include QTc prolongation (with a threshold of >500 ms). In the Hope-ICU trial more opiates and sedatives were administered in the placebo-group but alfa-2

agonists were not clearly protocolled, more agitation was present and 26% versus 11% antipsychotics' use in the placebo group. The subsyndromal delirium trial similarly found more agitation in the placebo group.

4. Healthcare perspective

A cost-effectiveness analysis of the Hope-ICU trial found that delirium increased cognitive dysfunction at 6 months and reduced quality of life, suggestive of potential cost-effectiveness of haloperidol (8).

5. Added value of the EuRIDICE trial

Based on this review of available pertinent literature after a thorough BIS-supported systematic search, the proposed trial in this grant application is expected to have important potential additional value:

The indication of haloperidol for ICU delirium will be delineated more clearly by this trial: does it decrease ICU brain dysfunction, associated long-term cognitive, functional and psychological outcomes? Is the intervention cost-effective? Are adverse events associated with haloperidol indeed concerning or actually negligible? Or: has haloperidol become obsolete, now that alternatives have been incorporated into clinical practice, mainly the atypical antipsychotics and alpha-2 agonists (dexmedetomidine and clonidine)? The EuRIDICE trial has a very strong potential to answers all of these questions.

A similar trial as EuRIDICE in the United States is on-going. However, US-based delirium research may not necessarily translate to European/Dutch settings as has been shown before (9), which justifies performing a second large multicentre clinical trial. Moreover, evidence on the pharmacological treatment of delirium is needed because of the lack of trials to date, and the level of evidence and generalizability of the efficacy findings for haloperidol will increase with a second trial.

Third, cost-effectiveness of the intervention will be assessed from a healthcare and societal perspective and family and patient experiences will be investigated as important secondary outcomes.

Further, we aim to include all critically ill patients, and not just the sickest, i.e. those on mechanical ventilation or in shock.

Existing guidelines and systematic reviews will have to be adapted on the basis of the results this proposed trial.

Acknowledgements:

Gerdien B. de Jonge (MSc), biomedical information specialist, Medical Library, Erasmus MC, is kindly acknowledged for her help in assembling the databases for the systematic review.

References

1. Girard TD, Pandharipande PP, Carson SS, Schmidt GA, Wright PE, Canonico AE, et al. Feasibility, efficacy, and safety of antipsychotics for intensive care unit delirium: The mind randomized, placebo-controlled trial. Crit Care Med. 2010;38(2):428-37.
2. Fonsmark L, Hein L, Nibroe H, Bundgaard H, de Haas I, Iversen S, et al. Danish national sedation strategy. Targeted therapy of discomfort associated with critical illness. Danish society of intensive care medicine (dsit) and the danish society of anesthesiology and intensive care medicine (dasaim). Dan Med J. 2015;62(4):C5052.
3. Barr J, Fraser GL, Puntillo K, Ely EW, Gelinas C, Dasta JF, et al. Clinical practice guidelines for the management of pain, agitation, and delirium in adult patients in the intensive care unit. Crit Care Med. 2013;41(1):263-306.
4. D. A. S. Taskforce, Baron R, Binder A, Biniek R, Braune S, Buerkle H, et al. Evidence and consensus based guideline for the management of delirium, analgesia, and sedation in intensive care medicine. Revision 2015 (das-guideline 2015) - short version. Ger Med Sci. 2015;13:Doc19.
5. Wang W, Li HL, Wang DX, Zhu X, Li SL, Yao GQ, et al. Haloperidol prophylaxis decreases delirium incidence in elderly patients after noncardiac surgery: A randomized controlled trial*. Crit Care Med. 2012;40(3):731-9.
6. Page VJ, Ely EW, Gates S, Zhao XB, Alce T, Shintani A, et al. Effect of intravenous haloperidol on the duration of delirium and coma in critically ill patients (hope-icu): A randomised, double-blind, placebo-controlled trial. Lancet Respir Med. 2013;1(7):515-23.
7. Al-Qadheeb NS, Skrobik Y, Schumaker G, Pacheco MN, Roberts RJ, Ruthazer RR, et al. Preventing icu subsyndromal delirium conversion to delirium with low-dose iv haloperidol: A double-blind, placebo-controlled pilot study. Crit Care Med. 2016;44(3):583-91.
8. Joachim M, Daniel FM, Annalisa C, Timothy A, Xiao BZ, Eugene WE, et al. Health evaluation and cost-effectiveness analysis from a randomized trial of haloperidol in the management of delirium in the critically ill (hope-icu trial). C24 sedation, delirium, and neurocognitive dysfunction in critical care. p. A4022-A.
9. van Eijk MM, van den Boogaard M, van Marum RJ, Benner P, Eikelenboom P, Honing ML, et al. Routine use of the confusion assessment method for the intensive care unit a multicenter study. Am J Respir Crit Care Med. 2011;184(3):340-4.
