# Supplementary material for: Efficacy of halopeRIdol to decrease the burden of Delirium In adult Critically ill patiEnts (EuRIDICE): study protocol for a prospective randomised multi-centre double-blind placebo-controlled clinical trial in the Netherlands
Source: BMJ Open. 2020 Sep 23;10(9):e036735. doi: 10.1136/bmjopen-2019-036735 (PMC7513600; doi:10.1136/bmjopen-2019-036735)
Supplement: Supplementary data [file bmjopen-2019-036735supp005.pdf]

Appendix 5: Drug Accountability

The study drug will be obtained from the hospital pharmacy of each participating ICU.

The research nurse of each participating ICU will record the number of the box with study drug for each patient in the CRF.

The research nurse of each participating ICU is responsible for retrieving the boxes with study drug. The amount of vials in the boxes will be counted for each patient and will be noted in the CRF.

The research nurse will return unused drug to the hospital pharmacy. The hospital pharmacy will destroy the vials with study drug and will also record this (double administration).

The pharmacist or another appropriate individual who is designated should maintain records of the product's delivery to the trial site, the inventory at the site, the use by each patient, problems and irregularities during injection, the maintenance of the blinding, and the return to the pharmacy of unused product(s). These records should include dates, quantities, batch/serial numbers, expiration dates (if applicable), and the unique code numbers assigned to the investigational product(s) and trial patients (if applicable). Investigators should maintain records that document adequately that the patients were provided the doses specified by the protocol and reconcile all investigational product(s).
