# Supplementary material for: Efficacy of halopeRIdol to decrease the burden of Delirium In adult Critically ill patiEnts (EuRIDICE): study protocol for a prospective randomised multi-centre double-blind placebo-controlled clinical trial in the Netherlands
Source: BMJ Open. 2020 Sep 23;10(9):e036735. doi: 10.1136/bmjopen-2019-036735 (PMC7513600; doi:10.1136/bmjopen-2019-036735)
Supplement: Supplementary data [file bmjopen-2019-036735supp006.pdf]

Appendix 6: Example of the patient consent form

Subject information and consent form for participation in medical scientific research

Effectiveness of haloperidol for the treatment of acute confusion (delirium) in critically ill patients

"Efficacy of halopeRIdol to decrease the burden of Delirium In adult Critically ill patiEnts (EuRIDICE): a prospective randomised multi-center double-blind placebo-controlled clinical trial"

(note: this Patient Information Letter is a translated version of the original Dutch document. It was Google translated and checked for readability by the study PI and coordinator)

Introduction

Dear Sir / Madam,

You receive this letter because you have been admitted to the Intensive Care Unit and have a chance (about 30%) of developing a delirium (sudden confusion) during admission. We ask you to participate in a medical-scientific study. Participation is voluntary. Your written permission is required to participate. Before you decide whether you want to participate in this study, you will receive an explanation of what the study entails. Please read this information carefully and ask the researcher if you have any questions. You can also ask the independent expert mentioned at the end of this letter for additional information. You can also discuss it with your partner, family or friends.
Further information about participating can be found in the attached brochure "Medical scientific research: general information for the test subject".

1. General information

This research was set up by Erasmus MC Rotterdam and is carried out by doctors and nurses in various hospitals in the Rotterdam region. This study requires a total of 742 subjects from different hospitals in the Rotterdam region. Erasmus MC's medical ethics review committee has approved this study. General information about the approval of research can be found in the brochure "Medical scientific research: general information for the test subject".

2. Purpose of the study

The aim of this study is to examine how safe and effective the drug haloperidol is for the treatment of acute confusion (delirium) in patients admitted to the Intensive Care Unit (ICU).

Smit L, *et al. BMJ Open* 2020; 10:e036735. doi: 10.1136/bmjopen-2019-036735

Haloperidol has been widely used for many years to treat delirium in ICU patients. However, whether haloperidol can reduce delirium once it has occurred has never been properly investigated. We therefore compare the effects of haloperidol with a placebo. A placebo is a drug without an active substance, a "fake" drug.

3. Background of the study

Delirium (sudden confusion) is a common problem in patients on an ICU. Delirium is associated with an increased risk of death, memory and thinking disorders and a reduced general condition in patients who leave the ICU. A common drug used to treat delirium in ICU patients is haloperidol. This medicine can have a beneficial effect on sudden anxiety and delusions (hallucinations), which often occur with delirium, but can also have side effects. The advantages and disadvantages of treatment with haloperidol have never been properly investigated in a so-called randomized-controlled study.

4. What it means to participate

Examination of eligibility

First we determine whether you can participate. We ask you or your close family about possible memory complaints indicating cognitive dysfunction before your admission to the ICU. When a pregnancy is possible, a pregnancy test is done. If you have memory problems that require further investigation or if you are pregnant, we will tell you and you cannot participate in the study. If you do not want to know if you are pregnant, you cannot participate in this study.
Sometimes during the examination of eligibility or follow-up study we find memory complaints or anxiety or depression complaints that require further medical examination. We will always share these test results with you. Further management of any test results indicating memory issues, anxiety etc, will be done through your own GP. The costs are covered by your own insurance.

Treatment

If you give permission to participate in this study and develop a delirium during admission to the ICU, study medication will be started. We will treat you with study medication for a maximum of 2 weeks. Half of the subjects receive the active agent (haloperidol), the other half the fake agent (placebo). Random selection determines whether you will receive haloperidol or placebo. You, your close relative or family member and all caregivers, such as nurses and the researcher, do not know which group you are in. If it is necessary for your health, it can be looked up.
General information can be found in the brochure "Medical scientific research: general information for the test subject".

Visits and measurements

Data will be collected for the study in the first two weeks. A description of the measurements made for this can be found in Appendix C.

For the examination, you will need to come to the hospital twice in 12 months after discharge to accurately test for memory and thinking disorders. A visit takes about 1.5 hours. If it is difficult for you to visit the hospital, we will try as much as possible to visit you at your home for the tests. You will also be sent questionnaires by post after discharge from the hospital, after 1, 3, 6 and 12 months after the ICU admission. The questions are about your experiences with and memories of the delirium and ICU admission, how fit you are and what physical limitations there may be. We also send questionnaires to your family member that record how they experienced your delirium and how they experience caring for you.
See appendix C for a schedule with an overview and explanation of the visits and measurements.

Different from usual care
If you decide to participate, you will be randomized and you will receive either haloperidol ór no haloperidol, but will be treated with other drugs to decrease delirium symptoms. These agents other than haloperidol are already used as standard care in the ICU and are also effective against the complaints associated with delirium (such as severe anxiety or delusions or hallucinations - i.e. seeing things that are not there, which is sometimes frightening). The other treatments are the same between both groups. After ICU admission, the follow-up is more extensive compared with usual care because the tests of memory and fitness are not routine. If you do not participate in the study, you will receive routine medication and this is usually the treatment with haloperidol in this hospital.

5. What is expected of you

In order for the research to run smoothly, it is important that you adhere to the following agreements.

The agreements are that you:

- do not participate in any other medical scientific research in which a treatment is tested.
- show up at appointments for follow-up visits.

It is important that you contact the researcher:

- if you no longer wish to participate in the study.
- if your contact details change.

Pregnancy
Women who are pregnant or breastfeeding cannot participate in this study. Treatment with haloperidol can have consequences for an unborn child. This mainly concerns movement disorders, such as muscle stiffness at birth, but it is currently insufficiently known whether haloperidol is entirely safe. A pregnancy test will be performed, so that it can be established with certainty that you are not pregnant and can safely participate in the study.

6. Possible side effects / complications

Haloperidol may cause side effects / adverse effects.
The *most common disadvantages* of haloperidol:

- slowing of nerve conduction in the heart, which can lead to arrhythmias
- muscle stiffness
- some drowsiness
- mild drop in blood pressure

Rare side effects are:

- muscle breakdown and high fever (so-called “neuroleptic malignant syndrome”)
- serious heart rhythm disorders in which the heart can (temporarily) stop.

The researchers consider the chance of unknown adverse effects / side effects of haloperidol to be virtually nihil, since haloperidol has been used for a long time and all side effects of this drug are well known.

Not receiving haloperidol (placebo) could also have adverse effects.
The possible disadvantages of not giving haloperidol are:

- more restlessness / agitation
- more delusions

When you become restless or suffer from delusions in delirium, other drugs can be given that also work well against anxiety and delusions. The side effects of these other medications are known and usually mild, including slowing of the heart rhythm and drop in blood pressure.

Measurements

The management during admission to the ICU are in accordance with normal practice and do not place an additional burden on you. After discharge, you will be asked to complete questionnaires and tests will be taken during a hospital visit or at home. These tests are not painful.

7. Possible advantages and disadvantages

It is important that you carefully weigh the possible pros and cons before you decide to participate.

Haloperidol can reduce the symptoms of delirium and shorten its duration, and reduce the long-term adverse effects (cognitive complaints and your general functional status), but this is not certain. At any time during treatment with study medication, delirium symptoms may recur or worsen. This does not directly mean that you are in the placebo group, because delirium can also persist despite treatment with haloperidol.

Disadvantages of participating in the study may include:

- Possible side effects of the study medicine (haloperidol) or not receiving the study medicine (placebo)

- Possible side effects of other medications given during the study period to reduce anxiety and hallucinations
- Possibly confronting questions about your functioning during and after discharge from the hospital.
- Visits to the hospital or home visits, telephone interviews or questionnaires sent can be a burden.

Participation in the study also means:
- That you will have to spend extra time;
- That (extra) tests are done;
- That you have to adhere to agreements for the best result of the research;

All these matters are described above under points 4, 5 and 6.

8. If you do not want to participate or want to quit the study

You should decide for yourself whether you want to participate in the study. Participation is voluntary. If you do not want to participate, you will be treated for delirium in the usual way according to the applicable procedures and protocols. The researcher can tell you more about the treatment options available and their advantages and disadvantages. There is a website with information about the treatment of delirium and the study: https://icudelirium.nl. If you do participate, you can always change your mind and stop, even during the study. You will then be treated for delirium in the usual way. You don't have to say why you quit. However, you must report this immediately to the researcher. The data collected up to that point will be used for the investigation.
If there is new information about the study that is important to you, the researcher will let you know. You will then be asked if you want to continue to participate.

9. End of the investigation

Your participation in the study stops if:
- all visits are over (according to the schedule / as described under point 4)
- you choose to quit
- the researcher thinks it is better for you to quit
- Erasmus MC, the government or the assessing medical ethics review committee decides to stop the research.

The whole study is finished when all participants have been treated and have been followed for the procedures for this study.
After processing all data, the researcher will inform you about the main results of the research. The investigator can also tell you whether you have had haloperidol or the placebo. If you do not want this, you can tell the investigator. The researcher then is not allowed to tell you.

10. Use and storage of your data

For this research it is necessary that your medical and personal data are collected and used. Each test subject is given a code that will appear on the data. Your name and other personal data that can directly identify you are omitted.

Your data

All your data remains confidential. Only the researchers working in your hospital know which code you have. We will send the data on to the coordinating investigator of the study, but only with that code, never by name. The key for the code remains with the local investigator. Also in reports about the research only that code is used.

Some people are allowed to view your medical and personal data. This is to check whether the research has been carried out properly and reliably. General information can be found in the brochure "Medical scientific research: general information for the test subject". People who can view your medical data are: members of the research team, the safety committee that monitors the investigation, an inspector who works for the client (Erasmus MC), the Health Care Inspectorate. The privacy of your personal data is always maintained. By signing the declaration of consent, you consent to the collection, storage and access of your medical and personal data.

Use of data on a later point

The researcher will keep your data for 15 years. We may use the data to do additional analyses for the study. This concerns research on delirium. You can indicate whether you agree with this on the consent form. You can always withdraw this permission. If no permission is given, you cannot participate in the study.

This research is also included in a public overview of medical scientific research, namely www.trialregister.nl. This website does not contain information that can be traced back to you as a person. However, the website can show a summary of the results. You can find this research under “EuRIDICE trial”. General information about the registration of studies can be found in the brochure "Medical scientific research: general information for the subject".

11. Insurance for test subjects

Everyone participating in this study is insured. The insurance covers damage from the investigation. Not all damage is covered. Appendix B provides more information about the insurance. It also states who can report the damage.

12. Inform GP

We always send your doctor a letter to let them know that you are participating in the study. The details of the tests taken after admission to the Intensive Care Unit are secret. If you wish, or if the researcher deems it necessary, these data can be shared with the GP.

13. Fee for Participation

The additional tests for the study cost you nothing. You will not be paid for participating in this study. You will receive a compensation of € 22 per visit for your (extra) travel costs and a € 5 lunch allowance per visit. You will not be reimbursed for a home visit.

14. Do you have any questions?

If you have any questions, please contact Dr. M. van der Jagt, principal investigator, or L. Smit, physician researcher. For independent advice on participation in this study, please contact the independent physician Dr. Dinis Dos Reis Miranda, anesthetist intensivist. He knows about this research, but is not involved in its implementation.
It is best to contact the complaints committee of Erasmus MC in case of complaints. All information can be found in Appendix A: Contact details.

15. Signing of consent form

When you have had enough time to consider participation, you will be asked to decide whether to participate in this study. If you give permission, we will ask you to confirm this in writing on the accompanying declaration of consent. By your written permission, you indicate that you have understood the information and that you agree to participate in the study.
The signature sheet is kept by the investigator. You will receive a copy or a second copy of this declaration of consent.

Thank you for your attention.

Smit L, *et al. BMJ Open* 2020; 10:e036735. doi: 10.1136/bmjopen-2019-036735

16. Annexes to this information

A. Contact details

B. Insurance information

C. Schedule of investigative actions

D. Consent form test subject

E. Brochure "Medical scientific research. General information for the subject (version March 2017) (to be supplied separately)

Appendix A: Contact details Erasmus Medical Center

Principal Investigator Erasmus Medisch Centrum
Dr. M. van der Jagt, neurologist-intensivist
Via the general telephone number Erasmus MC : 010- 704 07 04

Researcher Erasmus Medisch Centrum
Lisa Smit : physician researcher
Via the general telephone number Erasmus MC : 010- 704 07 04
Or via the research team

Independent physician Erasmus Medisch Centrum
Dr. Dinis Dos Reis Miranda, anesthetist-intensivist
Via the general telephone number Erasmus MC : 010- 704 07 04

Research team Erasmus Medisch Centrum
Ditty van Duijn : Research Coördinator Intensive Care
Patricia Ormskerk : Research Coördinator intensive Care
Alicija Vileito : Research Coördinator Intensive Care
Can be reached during office hours on: : 010-703 51 42

Complaints Committee
Erasmus Medisch Centrum
Can be reached on: : 010-703 31 98

Appendix B: Information about the insurance

The sponsor insures everyone who participates in this study. The insurance covers damage due to participation in the study. This applies to damage during the investigation or within four years after its end. You must report damage to the insurer within those four years.

The insurance does not cover all damage. At the bottom of this text is briefly mentioned what damage is not covered.
These provisions are set out in the Decree on compulsory insurance for medical research involving human subjects. This decision can be found on www.ccmo.nl, the website of the Central Commission for Human Research (see 'Library' and then 'Laws and regulations').

In the event of damage, you can contact the insurer directly.

The insurer of the study is:	
Name	: CNA Insurance Company Limited
Address	: Strawinskylaan 703
	: 1077 XX Amsterdam
Phone number	: 020 – 573 72 74
E-mail	: Esther.vanherk@cnahardy.com
Polis-number	: HCCD0416C
Contactperson	: Esther van Herk

The insurance covers

- € 650,000 per subject
- € 5,000,000 for the entire study
- € 7,500,000 per insurance year

The insurance does not cover the following damage:

- damage due to a risk about which you have been informed in the written information. This does not apply if the risk is more serious than anticipated or if the risk was very unlikely;
- damage to your health that would have occurred even if you had not participated in the study;
- damage caused by not (fully) following directions or instructions;
- damage to your offspring, as a result of a negative effect of the research on you or your offspring;
- damage caused by an existing treatment method when investigating existing treatment methods.

Smit L, *et al. BMJ Open* 2020; 10:e036735. doi: 10.1136/bmjopen-2019-036735

Appendix C - Measurements overview

During treatment with the study medication, a heart film will be made daily to determine whether the conduction in the heart is good, and muscle stiffness will be examined. You will also be asked every morning how you slept. In addition, delirium will be assessed daily. The attention is examined and the general condition is examined a few months after the IC admission.

The table below shows which normal care and which extra care you receive in the context of the ICU examination. The table also shows the moments you will be asked to visit the hospital (if desired and feasible for you and us, we will strive for home visits instead of hospital visits) or to complete questionnaires. The tests for memory and thinking take about 1 hour. The table shows the approximate minutes to complete the questionnaires (In brackets).

Moment (months)	Usual care on the ICU	Extra care during the study	Cognitive tests	Experiences related to delirium	General condition
**Once before participation**		- Questionnaire about your memory<br>- Pregnancy test (if applicable)			
**During study at the ICU**	- Delirium assessment (3x/day)	- EKG (1x/day)<br>- Test muscle stifness (1x/day)<br>- Sleep quality (1x/day)			
**0 (discharge hospital)**				*Participant*: Questionnaires (30)<br>*Proxy/family*: Questionnaire (2)	
**1**					Questionnaires (30)
**3**			Hospital or home visit	*Participant*: Questionnaires (35)<br>*Proxy/family*: Questionnaire (12)	Questionnaires (40)
**6**					Questionnaires (30)
**12**			Hospital or home visit		Questionnaires (40)

Smit L, *et al. BMJ Open* 2020; 10:e036735. doi: 10.1136/bmjopen-2019-036735

Appendix D: Consent form test subject

Effectiveness of haloperidol for the treatment of acute confusion (delirium) in critically ill patients

- I have read the information letter. I was also able to ask questions. My questions have been answered sufficiently. I had enough time to decide whether to participate.
- I know that participating is voluntary. I also know that I can decide at any time not to participate or to stop the research. I don't have to give a reason for that.
- I give permission to inform my GP that I am participating in this study and to inform them about test results of memory and thinking ability and possible anxiety or depression complaints, if the researcher deems this necessary.
- I know that some people can access my data. Those people are listed in this information letter.
- I consent to the collection and use of my data in the manner and for the purposes stated in the information letter.
- I give permission to keep my data at the research location for 15 years after this research.
- I know I should not be pregnant during the study (if applicable)
- I declare to □ **give**
  □ **not give** permission to contact me again after this investigation for a follow-up investigation
- I □ **do**
  □ **do not** want to be informed about which treatment I had or in which group I was.
- I want to participate in this study.

Name:
Signature: Date: __ / __ / __

I declare that I have fully informed this subject about the said study.

If during the research information becomes known that could influence the consent of the subject, I will inform him / her in good time.

Name researcher (or its representative):
Signature: Date: __ / __ / __

Additional information is provided by (if applicable):
Name:
Function:
Signature: Date: __ / __ / __

----

* Strike out what does not apply.

The subject will receive a full information letter, along with a copy of the signed consent form.
