# Supplementary material for: Efficacy of halopeRIdol to decrease the burden of Delirium In adult Critically ill patiEnts (EuRIDICE): study protocol for a prospective randomised multi-centre double-blind placebo-controlled clinical trial in the Netherlands
Source: BMJ Open. 2020 Sep 23;10(9):e036735. doi: 10.1136/bmjopen-2019-036735 (PMC7513600; doi:10.1136/bmjopen-2019-036735)
Supplement: Supplementary data [file bmjopen-2019-036735supp007.pdf]

BMJ Publishing Group Limited (BMJ) disclaims all liability and responsibility arising from any reliance placed on this supplemental material which has been supplied by the author(s)

Appendix 7: Unblinding Procedure

The study treatment will be unblinded after Database Lock. While the safety of patients should always take priority, maintenance of blinding is crucial to the integrity of a double-blind trial. Before this planned unblinding, the blinding for a specific patient should only be broken when information about the patient’s protocol treatment is considered necessary to manage Serious Adverse Events (emergency unblinding). Unblinding procedures should preferably be initiated only after consultation of the principal investigator/coordinating investigator or his/her representative. To initiate an emergency unblinding the pharmacy in charge of the randomisation list should be contacted. Breaking the blinding on a patient will be logged and reported to the coordinating Investigator within 24 hours following the unblinding procedure, using the Emergency Unblinding Form. It is considered a major protocol violation, after which the patient goes off protocol treatment (if applicable).

Smit L, *et al. BMJ Open* 2020; 10:e036735. doi: 10.1136/bmjopen-2019-036735
