# Supplementary material for: Efficacy of halopeRIdol to decrease the burden of Delirium In adult Critically ill patiEnts (EuRIDICE): study protocol for a prospective randomised multi-centre double-blind placebo-controlled clinical trial in the Netherlands
Source: BMJ Open. 2020 Sep 23;10(9):e036735. doi: 10.1136/bmjopen-2019-036735 (PMC7513600; doi:10.1136/bmjopen-2019-036735)
Supplement: Author's manuscript [file bmjopen-2019-036735.draft_revisions.pdf]

BMJ Open

BMJ Open is committed to open peer review. As part of this commitment we make the peer review history of every article we publish publicly available.

When an article is published we post the peer reviewers' comments and the authors' responses online. We also post the versions of the paper that were used during peer review. These are the versions that the peer review comments apply to.

The versions of the paper that follow are the versions that were submitted during the peer review process. They are not the versions of record or the final published versions. They should not be cited or distributed as the published version of this manuscript.

BMJ Open is an open access journal and the full, final, typeset and author-corrected version of record of the manuscript is available on our site with no access controls, subscription charges or pay-per-view fees (http://bmjopen.bmj.com).

If you have any questions on BMJ Open's open peer review process please email info.bmjopen@bmj.com

Efficacy of halopeRIdol to decrease the burden of Delirium In adult Critically ill patiEnts (EuRIDICE): study protocol for a prospective randomised multi-center double-blind placebo-controlled clinical trial

Journal:	*BMJ Open*
Manuscript ID	bmjopen-2019-036735
Article Type:	Protocol
Date Submitted by the Author:	09-Jan-2020
Complete List of Authors:	Smit, Lisa; Erasmus Medical Center, Department of Intensive Care Adults<br>Trogrlić, Zoran; Erasmus Medical Center, Department of Intensive Care Adults<br>Devlin , John; Northeastern University, Bouve College of Health Sciences; Tufts Medical Center, Division of Pulmonary, Critical Care and Sleep Medicine, Department of Pharmacy and Health Systems Sciences<br>Osse, Robert-Jan; Erasmus Medical Center, Department of Psychiatry<br>Ponssen, Huibert; Albert Schweitzer Hospital Location Dordwijk, Department of Intensive Care<br>Slooter, Arjen ; University Medical Centre Utrecht Brain Centre, Department of Intensive Care Medicine and UMC Utrecht Brain Center<br>Hunfeld, Nicole; Erasmus Medical Center, Department of Pharmacy and Department of Intensive Care<br>Rietdijk, Wim; Erasmus Medical Center, Department of Intensive Care Adults<br>Gommers, Diederik; Erasmus Medical Center, Department of Intensive Care Adults<br>van der Jagt, Mathieu; Erasmus Medical Center, Department of Intensive Care Adults
Keywords:	Delirium & cognitive disorders < PSYCHIATRY, INTENSIVE & CRITICAL CARE, Adult intensive & critical care < ANAESTHETICS

SCHOLARONE™
Manuscripts

BMJ

I, the Submitting Author has the right to grant and does grant on behalf of all authors of the Work (as defined in the below author licence), an exclusive licence and/or a non-exclusive licence for contributions from authors who are: i) UK Crown employees; ii) where BMJ has agreed a CC-BY licence shall apply, and/or iii) in accordance with the terms applicable for US Federal Government officers or employees acting as part of their official duties; on a worldwide, perpetual, irrevocable, royalty-free basis to BMJ Publishing Group Ltd ("BMJ") its licensees and where the relevant Journal is co-owned by BMJ to the co-owners of the Journal, to publish the Work in this journal and any other BMJ products and to exploit all rights, as set out in our licence.

The Submitting Author accepts and understands that any supply made under these terms is made by BMJ to the Submitting Author unless you are acting as an employee on behalf of your employer or a postgraduate student of an affiliated institution which is paying any applicable article publishing charge ("APC") for Open Access articles. Where the Submitting Author wishes to make the Work available on an Open Access basis (and intends to pay the relevant APC), the terms of reuse of such Open Access shall be governed by a Creative Commons licence – details of these licences and which Creative Commons licence will apply to this Work are set out in our licence referred to above.

Other than as permitted in any relevant BMJ Author's Self Archiving Policies, I confirm this Work has not been accepted for publication elsewhere, is not being considered for publication elsewhere and does not duplicate material already published. I confirm all authors consent to publication of this Work and authorise the granting of this licence.

For peer review only

Efficacy of halopeRIdol to decrease the burden of Delirium In adult Critically ill patiEnts (EuRIDICE): study protocol for a prospective randomised multi-center double-blind placebo-controlled clinical trial

Lisa Smit – l.smit@erasmusmc.nl (1), Zoran Trogrlic – z.trogrlic@erasmusmc.nl (1), John W. Devlin - j.devlin@northeastern.edu (2), Robert Jan Osse - r.osse@erasmusmc.nl (3), Huibert H. Ponssen - H.H.Ponssen@asz.nl (4), Arjen J.C. Slooter - A.Slooter-3@umcutrecht.nl (5), Nicole G.M. Hunfeld - n.hunfeld@erasmusmc.nl (1, 6), Wim J.R. Rietdijk - w.rietdijk@erasmusmc.nl (1), Diederik Gommers - d.gommers@erasmusmc.nl (1), Mathieu van der Jagt – m.vanderjagt@erasmusmc.nl* (1), on behalf of the EuRIDICE study group.

1) Erasmus MC-University Medical Center, Department of Intensive Care Adults, Rotterdam, The Netherlands, Netherlands

2) Bouve College of Health Sciences, Northeastern University; Division of Pulmonary, Critical Care and Sleep Medicine, Department of Pharmacy and Health Systems Sciences, Boston, United States

3) Erasmus MC-University Medical Center, Department of Psychiatry, Rotterdam, The Netherlands, Netherlands

4) Albert Schweitzer Hospital, Department of Intensive Care, Dordrecht, Netherlands

5) University Medical Center Utrecht, Utrecht University, Department of Intensive Care Medicine and UMC Utrecht Brain Center, Utrecht, The Netherlands

6) Erasmus MC-University Medical Center, Departments of Intensive Care Adults and Pharmacy, Rotterdam, The Netherlands, Netherlands

*Corresponding author:

Department of Intensive Care, room Ne-415

Erasmus MC

For peer review only

PO BOX 2040

3000 CA Rotterdam

E-mail: m.vanderjagt@erasmusmc.nl

Telephone number: +31107040704

Protocol version: November 16th 2017, Version 2

Word count: 4326 words

For peer review only

ABSTRACT

Introduction: Delirium in critically ill adults is associated with prolonged hospital stay, increased mortality and greater cognitive and functional decline. Current practice guideline recommendations advocate the use of non-pharmacologic strategies to reduce delirium. The routine use of scheduled haloperidol to treat delirium is not recommended given a lack of evidence regarding its ability to resolve delirium nor improve relevant short and longer-term outcomes. This study aims to evaluate the efficacy and safety of haloperidol for the treatment of delirium in adult critically ill patients to reduce days spent with coma or delirium.

Methods and analysis: EuRIDICE is a prospective, multicentre, randomized, double-blind, placebo-controlled, trial. Study population consists of adult ICU patients without acute neurologic injury who have delirium based on a positive Intensive Care Delirium Screening Checklist (ICDSC) or Confusion Assessment Method for the ICU (CAM-ICU) assessment. Intervention is intravenous haloperidol 2.5 mg (or matching placebo) every 8 hours, titrated daily based on ICDSC or CAM-ICU positivity to a maximum of 5 mg every 8 hours, until delirium resolution or ICU discharge. Main study endpoint is ICU days free of delirium and coma (DCFD) up to 14 days after randomisation. Secondary endpoints include 1) 28-day and 1-year mortality; 2) cognitive and functional performance at 3 and 12 months; 3) patient- and family delirium and ICU experience; 4) psychological sequelae during and after ICU stay; 4) safety concerns associated with haloperidol use; and 5) cost-effectiveness. Differences in

For peer review only - http://bmjopen.bmj.com/site/about/guidelines.xhtml

DCFDs between haloperidol and placebo group will be analysed using Poisson regression analysis. Study recruitment started in February 2018 and continues.

Ethics and dissemination: The study has been approved by the Medical Ethics Committee of the Erasmus University Medical Centre Rotterdam (MEC2017-511). Its results will be disseminated via peer-reviewed publication and conference presentations.

Trial registration: ClinicalTrials, NCT03628391. Registered 14 August 2018 - https://clinicaltrials.gov/ct2/show/NCT03628391.

Strengths and limitations of this study

- This study is the first European sufficiently powered randomised multi-center double-blind placebo-controlled clinical trial;
- Extensive neurocognitive testing will be conducted with a valid test battery in order to assess cognitive impairment at 3 and 12 months after ICU admission;
- We will assess patient- and family experiences associated with delirium as a novel outcome;

INTRODUCTION

Delirium occurs in up to 80% of patients admitted to the Intensive Care Unit (ICU) (1, 2) and is associated with greater ICU and post-ICU mortality (2). Cognitive dysfunction and functional decline after critical illness is common, frequently persists for months after ICU discharge, and is worse among patients who experience delirium (2, 3). The symptoms and sequelae of delirium, including fear, anxiety, disrupted sleep, and post-traumatic stress disorder, may persist for months after ICU discharge. The health and societal costs of delirium are estimated to exceed $10 billion per year in the USA alone (4).

Given the burden and costs of delirium in critically ill adults, substantial research efforts have been devoted to identify safe and effective strategies to treat it. Current evidence and practice guideline recommendations advocate the use of non-pharmacologic strategies to reduce delirium, including avoidance of benzodiazepine sedation, early mobilization and

For peer review only

For peer review only - http://bmjopen.bmj.com/site/about/guidelines.xhtml

the use of sleep improvement protocols. The routine use of medication-based interventions to treat delirium, other than treatments to reduce the agitation that sometimes accompanies it, are not recommended (5, 6). The routine use of scheduled haloperidol to treat delirium is not currently recommended given a lack of current evidence regarding its ability to resolve delirium and its symptoms, nor improve relevant short and longer-term outcomes.

At the time this protocol was finalized, two randomized, placebo-controlled trials had evaluated haloperidol for ICU delirium prophylaxis or treatment and found haloperidol use did not affect days spent with delirium, days of mechanical ventilation, nor time spent in the ICU or hospital (7, 8). In one of these randomized controlled trials (RCTs), haloperidol use was associated with less agitation (7). Importantly, both studies were small (a combined total of 212 patients were enrolled), the ABCDEF bundle (a multimodal ICU bundle shown to reduce delirium by 50%)(9) was not routinely used, the effect of haloperidol on delirium-related symptoms was not evaluated, and the post-ICU, longer-term outcomes were not considered. Whether the response to haloperidol was different between patients with hyperactive versus hypoactive delirium was also not evaluated. The impact of haloperidol on patients'- and families' experiences with delirium after ICU discharge remains unknown. Whether long-term mortality is causally related to delirium or simply the persistent cognitive and functional decline associated with critical illness can only be established through a randomised trial (10). Moreover, the use of haloperidol in critically ill adults is not without potential safety concerns given it may prolong the QTc interval, induce extrapyramidal effects and cause oversedation. Despite haloperidol's lack of proven efficacy and the safety concerns associated with its use, haloperidol continues to be widely used in ICUs to treat of delirium (11).

In light of the above evidence gaps that were identified at the time this trial was conceptualized, there is a clear need for a large, multi-center, randomised controlled trial to better define the efficacy and safety of haloperidol to treat delirium in critically ill adults. This report describes the protocol for a large, multicentre, randomized, placebo-controlled,

For peer review only

For peer review only - http://bmjopen.bmj.com/site/about/guidelines.xhtml

haloperidol delirium trial that recently started enrolling patients across multiple ICUs in the Netherlands.

METHODS AND ANALYSIS

Study design

Randomized, double-blind, placebo-controlled trial of haloperidol for the treatment of delirium in patients admitted to one of six participating ICUs in the Rotterdam area in the Netherlands. See Appendix 1 for the participating hospitals.

Study population

Consecutive adults admitted to one of the participating ICUs.

Eligibility criteria

Inclusion criteria for eligibility:

1. Age ≥ 18 years
2. Admitted to the ICU.

Exclusion criteria for eligibility:

1. Admitted to the ICU with an acute neurological diagnosis (including acute stroke, traumatic brain injury, intracranial malignancy, anoxic coma). Prior non-acute stroke or another neurological condition without cognitive deterioration is not an exclusion criterion.
2. Pregnancy or lactation
3. History of ventricular arrhythmia including “torsade de pointes” (TdP)
4. Known allergy to haloperidol
5. History of dementia or an Informant Questionnaire on Cognitive Decline in the Elderly (IQCODE) score ≥ 4 (12)
6. History of malignant neuroleptic syndrome or parkinsonism (either Parkinson’s disease or another hypokinetic rigid syndrome)

For peer review only - http://bmjopen.bmj.com/site/about/guidelines.xhtml

7. Schizophrenia or other psychotic disorder
8. Inability to conduct valid delirium screening assessment (e.g. coma, deaf, blind) or inability to speak the Dutch language
9. Expected to die within 24 hours or leave the ICU within 24 hours

Inclusion criteria for randomisation:

1. Delirium, as assessed with the Intensive Care Delirium Screening Checklist (ICDSC ≥ 4) or the Confusion Assessment Method for the ICU (positive CAM-ICU assessment), at the time of ICU admission or any ICU day after ICU admission.
2. Written informed consent obtained from the patient or their legal representative
3. All eligibility *in*clusion criteria (from above) are still met.

Exclusion criteria for randomisation:

1. Prolonged QT-interval (QTc > 500ms)
2. (recent) “Torsade de pointes” (TdP)
3. (recent) Malignant neuroleptic syndrome or parkinsonism
4. Evidence of acute alcohol (or substance) withdrawal requiring pharmacological intervention (e.g. benzodiazepines or alfa-2 agonist) to treat
5. The patient is expected to die within 24 hours or expected to leave the ICU within 24 hours.
6. No (previously) signed informed consent by patient or representative
7. Current participation in another intervention trial that is evaluating a medication, device or behavioural intervention

Study endpoints

Main study endpoint:

ICU delirium- and coma free days (DCFDs) (up to 14 days after randomisation).

Secondary study endpoints:

For peer review only - http://bmjopen.bmj.com/site/about/guidelines.xhtml

ICU stay

- Demographics: age, sex, admission diagnosis category, APACHE II and APACHE IV, SOFA, ICU days before study entry, pre-admission delirium duration in participants with delirium on admission.
- Richmond Agitation Sedation Scores (RASS)
- Maximum ICU Mobility Scale (IMS (13)) and day of max IMS.
- Quality of sleep (Richards-Campbell Sleep Questionnaire [RCSQ] (14) and with a visual analogue scale between 1-7 assessing the sleep quality according to the nurse).
- Use of “escape medication” for hallucinations and/or agitation (including atypical antipsychotics, alpha-2 agonists, GABA-agonists, opiates and “open-label” haloperidol).
- Daily study drug dose corrected for body weight (mg/kg).
- Auto-extubation rate, removal of invasive devices (intravenous/-arterial catheters, drains and tubes).
- Adverse drug associated events (prolonged QTc by EKG, muscle rigidity and other associated movements disorders [Simpson Angus Scale (15)] and ventricular arrhythmia’s including torsade de pointes).
- Blood pressure will be recorded previous to and 1 hour after the first study drug dose (2.5mg equivalent) and 1 hour after the first 5mg equivalent.
- Daily respiratory status (regarding endotracheal intubation and mechanical ventilation)
- Time from randomisation to first resolution of delirium
- Time to “readiness for discharge from the ICU”

Hospital discharge

For peer review only

For peer review only - http://bmjopen.bmj.com/site/about/guidelines.xhtml

- Patient and family-member well-being and experiences associated with delirium during and after ICU stay with the ICU Memory Tool (ICU-MT (16)) and Delirium Experience Questionnaire (DEQ (17)).

28 days after randomization

- Mortality rate

3 months after randomization

- Cognitive outcomes with a brief cognitive assessment battery of validated and repeatable measures of general cognition, memory, language, processing speed, attention and executive functioning and mood (Montreal Cognitive Assessment [MOCA](18), Rey Auditory Verbal Learning Test(19), Semantic fluency(20), Digit Span [WAIS-IV](21), Trail making tests A and B(22), Boston naming Test [short version](23), Hospital Anxiety and Depression Scale [HADS](24)).
- Functional outcomes and quality of life (Short Form-36 [SF-36](25)).
- Patient and family-member well-being and experiences associated with delirium during and after ICU stay with the ICU Memory Tool (ICU-MT (16)), Delirium Experience Questionnaire (DEQ (17)) and Caregiver Strain Index (CSI (26)).
- Posttraumatic stress syndrome (PTSS) in participants and family-members with the Impact of Event Scale – Revisited (IES-R)(27).

12 months after randomization

- Cognitive outcomes with a brief cognitive assessment battery of validated and repeatable measures of general cognition, memory, language, processing speed, attention and executive functioning and mood (Montreal Cognitive Assessment [MOCA](18), Rey Auditory Verbal Learning Test(19), Semantic fluency(20), Digit Span [WAIS-IV](21), Trail making tests A and B(22), Boston naming Test [short version](23), Hospital Anxiety and Depression Scale [HADS](24)).
- Functional outcomes and quality of life (Short Form-36 [SF-36](25)).
- Mortality rate

For peer review only

A cost-effectiveness analysis will be performed in collaboration with the Department of Health Policy and Management of Erasmus University Rotterdam (see Appendix 2 for more detailed explanation).The tools for the secondary outcomes are mentioned in Table 1 with overview of timing of assessments.

Treatment of subjects

Investigational product:

Name: Haldol (haloperidol)

Mechanism: butyrophenone-derived anti-psychotic with mainly dopamine-2 receptor antagonistic properties

Placebo consists of sodium chloride for injection. Medical staff, patients and family will be blinded to the product containing haloperidol/placebo.

Summary of findings from clinical studies and of known and potential risks and benefits*:*

See: Summary of Product Characteristics (SPC) in Appendix 3 and Systematic Review (Appendix 4).

Dosages, dosage modifications and method of administration*:*

The following dosing scheme will be used: start with haloperidol/placebo (further called: “study drug”) 2.5mg IV q8h (because of delirium screening once every 8-hour shift) and increase to a maximum dose of 5mg IV q8h when delirium persists beyond the next 8-hour shift. Doses will be reduced (50% of dose) in the very old elderly (age ≥ 80 years). The study drug dose will be decreased (when dosage is 5mg IV q8h) or stopped (when dose is 2.5mg IV q8h) when delirium has resolved for the next 24 hours. Dosages can be lowered also at the discretion of the treating physician in case of evident rigidity, which is in line with current routine practice. Standard clinical practice for the administration of haloperidol will be followed.

For peer review only - http://bmjopen.bmj.com/site/about/guidelines.xhtml

Description and justification of route of administration and dosage:

Administration of the study intervention via the IV (versus the oral or enteral) route is the most feasible in critically ill patients – a population where gastrointestinal dysfunction is prevalent and haloperidol absorption (i.e., bioavailability) could be compromised. The dose of haloperidol or placebo equivalent to be used in the study is based on the following consideration: 1. PK/PD; 2. Efficacy and 3. Safety. A (pilot) study in Erasmus Medical Center (n=14 critically ill patients, abstract presented at European Society of Intensive Care Medicine 2016) showed no adverse events (e.g. no QTc > 500ms), low serum levels (1.5-2.2µg/L) and no clear relation between serum level and delirium resolution with haloperidol dosages up to 2mg IV q8h (or: 3 x 2mg IV). A feasibility trial of haloperidol for ICU delirium (MIND-trial (8)) that used an average total daily dosage of 15 mg orally found higher serum levels (interquartile range 2.85-5.8 µg/L). No differences were found in QTc prolongation between treatment groups and placebo in this trial. None of these trials demonstrated clinically important safety concerns associated with haloperidol administration. Finally, a recently published trial of haloperidol for ICU delirium using haloperidol/placebo 10mg IV q12h, did not report any safety issues, using a QTc cut-off for safety of 550ms, which may be regarded an indirect signal that such dosages are feasible and safe (28).The maximum dose of haloperidol of up to 5mg IV q8h was further chosen because a previous Dutch guideline advocating the use of haloperidol recommended an IV haloperidol treatment dose of up to 20 mg/24h period (29). In our protocol, we chose q8h dosing (titrated up to 15mg daily) given the greater potential susceptibility of critically ill adults to the side effects of haloperidol, and the fact that this dosage is in line with existing haloperidol delirium protocols in several of the participating ICUs.

Patient assessments:

Rigidity will be monitored with the Simpson-Angus scale (15) and the Barnes Akathisia Rating Scale (30) (see “Secondary study endpoints”) for study purposes only. The QTc

For peer review only - http://bmjopen.bmj.com/site/about/guidelines.xhtml

For peer review only

interval will be measured daily before the administration of the second daily (afternoon) dose using a 12-lead EKG. When the QTc interval is found to be prolonged (> 500ms or an increase from baseline (=at randomisation) of ≥ 60ms (31, 32)), all non-study medications having the potential to prolong the QTc will be held if clinically feasible. A Standard Operating Procedure (S.O.P.) lists the drugs known to prolong the QTc. Eight hours later, if QTc prolongation persists, study medication will be held or tapered according to the S.O.P. and only resumed when the EKGs (evaluation frequency increased to q8h in this situation) reveal QTc prolongation to have dissipated.

<u>General medical management at participating ICUs:</u>

In the six original participating ICUs, institutional delirium guidelines, based on the 2013 PAD guidelines and a Dutch ICU delirium guideline, were rigorously implemented over a three-year period (2012 to 2015) (6, 33, 34). During the inclusion period of the current trial, spot-checks will be performed by members of the investigative team at each center to confirm delirium screening accuracy.

<u>Preparation and labelling of Investigational Medicinal Product:</u>

Preparation and labelling will be done by the trial pharmacist ("Apotheek A15") according to GMP guidelines. Apotheek A15 is certified for these procedures. Trial medication will be dispensed to the pharmacies of the trial sites by the Hospital Pharmacy of Erasmus MC. See Appendix 5 for a description of the drug accountability.

<u>Escape medication:</u>

Knowing that half the subjects will be administered placebo, we anticipate two issues may affect the clinical management of enrolled patients: 1) agitation and 2) hallucinations.

Agitation management will be based on the following principles: a) treat pain first with opioids; b) use alpha-2 agonist for agitation that either persists or is not caused by pain; c) GABA agonists (e.g. benzodiazepines or propofol) are discouraged, but can be used on a

For peer review only - http://bmjopen.bmj.com/site/about/guidelines.xhtml

short-term basis for the treatment of severe agitation (RASS ≥ 2) that cannot be effectively managed by other means.

Hallucination management will be based on the following principles: a) pharmacological treatment may be withheld if the patient indicates they are not in distress; b) for a patient in distress, a low-dose atypical antipsychotic (e.g., quetiapine 12.5mg q8h) may be administered on a short-term basis until the distress resolves.

Because of the pragmatic design of this trial, within these boundaries, the treatment and dose of escape medication is left to the treating physician, since these are part of routine practice. However, before start of randomisation these management principles for agitation and hallucination will be thoroughly implemented first with the help of detailed S.O.P.'s to enhance uniformity in participating centres. Adherence to escape medication regimens will be closely monitored. Open-label haloperidol administration is strongly discouraged during the trial but can be used if the ICU team considers it necessary for acute breakthrough delirium symptoms that cannot be managed within the management boundaries outlined above. Open-label haloperidol will be documented.

Randomisation, blinding and treatment allocation

Legal representatives of eligible patients (when the patient is sedated or otherwise temporarily unable to consent) or the patient him-/herself will be asked for informed consent shortly after admission when the patient has no delirium, or as soon as possible after admission when the patient already has delirium. In this study the presence of delirium will be considered to be confirmed when the beside nurse deemed the patient to have delirium based on assessment with the ICDSC or CAM-ICU, given the previous large-scale implementation project (33).

Delirious patients who fulfil all inclusion but no exclusion criteria, and for whom written informed consent has been obtained (as recorded in medical file), will be randomised. Randomisation coordination and start of a new Case Record Form (CRF) will be guided by the Electronic Data Capture (EDC) system of ALEA, constructed by the Clinical Trial Center

For peer review only - http://bmjopen.bmj.com/site/about/guidelines.xhtml

(CTC) of the Erasmus Medical Center and calibrated with the coordinating (Erasmus MC) and local pharmacies. We will randomise the recruited patients using a block design of 8 patients in one block, and one block is assigned to a center. We will have 8 batches (numbered 1 through 8) of treatment and placebo, with 4 batches of placebo and 4 treatment (haloperidol). Each block will have a random assignment of 8 batch numbers, having four placebo and four haloperidol patients included (a combination of 1 to 4 and 5 to 8 in random order). After 8 patients are included in the study (i.e., a block is full), a new block will be assigned to a center.

Blinding of the medication will be performed by the pharmacy, based on a randomisation list that will be generated electronically through a randomisation module in the EDC system of ALEA. Randomisation will be stratified per study center (i.e. equal number of patients in both study groups, see “statistical analysis” paragraph). Only the involved pharmacists and the trial statistician are aware of the contents of each medication kit, so they can unblind a patient in case of an emergency. Except for the hospital’s pharmacist responsible for the randomisation list, all other involved personnel with the study, caregivers, patients or their representatives will remain unaware of the treatment groups until the time of Database Lock. The Unblinding procedure is specified in Appendix 6.

Follow-up procedures will be performed according to designated S.O.P.’s. When possible and preferred by patients or families, questionnaires will be sent or visits planned at home when possible, e.g. for incapacitated participants.

Withdrawal of individual subjects

Subjects can leave the study at any time for any reason if they wish to do so without any consequences. The investigator can decide to withdraw a subject from the study for urgent medical reasons.

Follow-up of subjects withdrawn from treatment

For peer review only

For peer review only - http://bmjopen.bmj.com/site/about/guidelines.xhtml

Data of withdrawn patients will remain in the database for statistical analysis purposes but will not be subject to follow-up. When patients specifically withdraw their consent for usage of their data, these data will be removed from the database and excluded from all analyses.

Premature termination of the study

The sponsor may decide to terminate the study prematurely based on the following criteria:

- There is evidence of an unacceptable risk for study patients (i.e. safety issue)
- There is reason to conclude that continuation of the study cannot serve a scientific purpose following confirmation of the Data Safety Monitoring Board (DSMB)
- The DSMB recommends to end the trial based on viable arguments other than described above.

The following stopping rules have been determined by the DSMB and have been laid down in a DSMB charter:

- Early stopping of one individual participant, for example, to clear benefit or harm of a treatment or the occurrence of serious adverse reactions or events in one patient. In this case de-blinding of this single patient may be necessary.
- Stopping of the trial as a whole to clear benefit or harm of a treatment or the occurrence of serious adverse reactions or events. As a result, further patient enrolment will be stopped. Deblinding may be necessary for all patients.

Reasons to stop the study include:

- Advice to do so from DSMB
- Interim analysis shows a significant benefit difference between the treatment groups which will not be expected to change after inclusion of all subject as per the power analysis.

For peer review only - http://bmjopen.bmj.com/site/about/guidelines.xhtml

SUSAR's are not expected due to the vast experience in clinical practice with the study drug (haloperidol).

If the study is terminated the Medical Ethics Committees of all participating hospital and the CCMO will be notified.

Safety reporting

AEs, SAEs and SUSARs:

Adverse events (AEs)

Adverse events are defined as any undesirable experience occurring to a subject during the study, whether or not considered related to the investigational product. Since patients admitted to an ICU are critically ill and present with many AEs, only possible adverse drug related events (on days of study drug administration: prolonged QTc by EKG, muscle rigidity and associated movements disorders [Simpson Angus Scale]) as indicated by the subject or observed by the investigator or his staff occurring from the date of randomisation until 14 days later or discharge from ICU or death (whichever comes first), will be recorded in the CRF. In addition, the following AEs will be assessed daily during 14 days after randomisation: epilepsy, tachycardia, hypotension (not explained otherwise), hepatic dysfunction (not explained otherwise), leucopenia (not explained otherwise), bronchospasms (not explained otherwise).

Serious adverse events (SAEs)

A SAE is any untoward medical occurrence or effect, occurring during the 14-day study period at the ICU, that (the SAEs for the purpose of the study are shown in *Italics* per item)

- results in death;
  - *death will always be reported as an SAE*
- is life threatening (at the time of the event);
  - *ventricular arrhythmia or malignant neuroleptic syndrome*

For peer review only

For peer review only - http://bmjopen.bmj.com/site/about/guidelines.xhtml

- requires hospitalisation or prolongation of existing inpatients' hospitalisation;
  - *Not to be expected; only applicable when the site investigator is able to explicitly show a relationship*
- results in persistent or significant disability or incapacity;
  - *Not to be expected; only applicable when the site investigator is able to explicitly show a relationship*
- is a congenital anomaly or birth defect; (*N/A*) or
- any other important medical event that did not result in any of the outcomes listed above due to medical or surgical intervention but could have been based upon appropriate judgement by the investigator.

Statistical analysis

Primary and secondary study parameter(s):

Statistical analysis will be done according to intention-to-treat-principle. All randomised participants will be included. The primary outcome is DCFDs, defined as the number of days in the first 14 days after randomisation during which the patient is alive without delirium and not in coma from any cause (7). Differences between DCFDs between the haloperidol group and placebo group will be analyzed using Poisson regression analysis, with adjustment for differences in baseline characteristics between treatment groups (when present) and for the different centers. For cognitive and functional outcomes assessed with designated test-batteries, non-parametric or parametric tests will be used depending on normality of scaled test-results. Mortality risk will be assessed as a binary end-point. A more detailed statistical analysis plan, to be drawn up before Data Base Lock, will be drafted for publication separately.

Interim analysis:

For peer review only

Pre-planned interim analyses will be performed at 1/3 and 2/3 of the trial's course (first analysis ideally estimated at 6 months after start of trial), as determined by the DSMB charter or otherwise when the DSMB requests it.

Sample size calculation

To achieve statistically significant results with (p<.05) with a power of 90% and a true treatment difference of one day for the primary outcome (from 3.2 DCFDs in the placebo group to 4.2 in the haloperidol group, SD in both groups is equal to 4.2), 371 patients are needed in each group (n=742). These estimates are derived from the previous implementation study, which included 4727 patients in three 4-month periods in the same six participating ICUs and found delirium incidence of 27% (and increase of DCFDs from 60% to 70%)(33). Consequently, presuming an informed consent rate of 40%, we need 18-months to encounter 1900 patients with a newly diagnosed delirium to include the required 742 patients. Because of estimated work-load due to follow-up visits, including e.g. neurocognitive testing, we propose to select a convenience sample of 2/3 of ICU survivors (estimated around 400 of 575 survivors) as a random sample for the cognitive, functional and secondary outcome variables.

Patient and Public Involvement

During the design and conduct of the study we involved two ex-ICU patients as patient-perspective representatives. The primary research question, its outcome measures, and the burden of the intervention have been assessed and found relevant by these patient-representatives. The role and tasks of the patient-representatives for the study have been detailed as: 1) to help select meaningful assessment-tools of patient and family experiences during and after ICU stay, 2) act as liaison between the study management team and the Dutch foundation "Family and patient Centered Intensive Care" (FCIC; one representative is a formal representative for FCIC), 3) act as members of the Stakeholders group to provide advice on the study contents, execution and course at on a regular basis to ensure the

For peer review only

For peer review only - http://bmjopen.bmj.com/site/about/guidelines.xhtml

patient and family perspective, 4) advise on the contents of the Patient Information Form (PIF) and the informed consent procedure, 5) advise on ways to minimise loss to follow-up for the functional and cognitive outcome assessments, 6) advise on contents and organisation of symposia during the study on delirium and its consequences with the aim to better inform participants of the study and their family members and maximize their involvement, 7) advise on the contents of the supporting website of the trial. Study participants will be informed about the most important results of the trial, either by post or symposium, when they indicate this on the informed consent letter.

ETHICS AND DISSEMINATION

The study has been approved by the Medical Ethics Committee of the Erasmus University Medical Centre Rotterdam (MEC2017-511) and the Institutional Review Boards of participating sites. The study will be conducted according to the principles of the Declaration of Helsinki (version, date, see for the most recent version: www.wma.net) and in accordance with the Medical Research Involving Human Subjects Act (WMO) and other guidelines, regulations and Acts.

Recruitment and consent

Recruitment of eligible patients will be done upon admission. Informed consent for *possible* participation (i.e. only when participants develop delirium at the ICU) will be obtained from subjects who are not expected to leave the ICU within the first 24 hours after admission and are not yet delirious. The informed consent will be obtained from the patient or (if the patient is unable to consent) from patient's representative. This procedure of prior request for informed consent will facilitate randomisation when the patient indeed develops delirium, because randomisation can then be performed 24/7 since informed consent is already obtained and delirium often surfaces during the evening and night when obtaining informed consent is difficult. The informed consent procedure will be clearly delineated from the randomisation procedure. Importantly, when a patient with prior informed consent develops

For peer review only

For peer review only - http://bmjopen.bmj.com/site/about/guidelines.xhtml

delirium and can thus be randomised, still a pre-randomisation check with regard to in- and exclusion criteria will be performed to confirm that the patient fulfils the inclusion, and not the exclusion criteria (because this may change over time). A team of dedicated research and ICU nurses and physicians (local PI, PhD student, PI, post-doc) will be trained to perform the informed consent procedures and help with the randomisations. Moreover, a 24/7 study consultation telephone number will be opened to help with problems or question during the study. A second type of randomisation concerns patients who are delirious upon admission to ICU. These patients' next-of-kin will be asked to grant permission to participate by means of informed consent when they are legally representative for the patients and the patient has no contraindications. After informed consent is obtained, the patient can be randomised.

REFERENCES

1. Salluh JI, Soares M, Teles JM, Ceraso D, Raimondi N, Nava VS, et al. Delirium epidemiology in critical care (DECCA): an international study. Crit Care. 2010;14(6):R210.
2. Ely EW, Shintani A, Truman B, Speroff T, Gordon SM, Harrell FE, Jr., et al. Delirium as a predictor of mortality in mechanically ventilated patients in the intensive care unit. JAMA. 2004;291(14):1753-62.
3. Pandharipande PP, Girard TD, Ely EW. Long-term cognitive impairment after critical illness. N Engl J Med. 2014;370(2):185-6.
4. Milbrandt EB, Deppen S, Harrison PL, Shintani AK, Speroff T, Stiles RA, et al. Costs associated with delirium in mechanically ventilated patients. Crit Care Med. 2004;32(4):955-62.
5. Reade MC, Eastwood GM, Bellomo R, Bailey M, Bersten A, Cheung B, et al. Effect of Dexmedetomidine Added to Standard Care on Ventilator-Free Time in Patients With Agitated Delirium: A Randomized Clinical Trial. JAMA. 2016;315(14):1460-8.
6. Devlin JW, Skrobik Y, Gelinas C, Needham DM, Slooter AJC, Pandharipande PP, et al. Clinical Practice Guidelines for the Prevention and Management of Pain, Agitation/Sedation, Delirium, Immobility, and Sleep Disruption in Adult Patients in the ICU. Crit Care Med. 2018;46(9):e825-e73.
7. Page VJ, Ely EW, Gates S, Zhao XB, Alce T, Shintani A, et al. Effect of intravenous haloperidol on the duration of delirium and coma in critically ill patients (Hope-ICU): a randomised, double-blind, placebo-controlled trial. Lancet Respir Med. 2013;1(7):515-23.
8. Girard TD, Pandharipande PP, Carson SS, Schmidt GA, Wright PE, Canonico AE, et al. Feasibility, efficacy, and safety of antipsychotics for intensive care unit delirium: the MIND randomized, placebo-controlled trial. Crit Care Med. 2010;38(2):428-37.
9. Barnes-Daly MA, Phillips G, Ely EW. Improving Hospital Survival and Reducing Brain Dysfunction at Seven California Community Hospitals: Implementing PAD Guidelines Via the ABCDEF Bundle in 6,064 Patients. Crit Care Med. 2017;45(2):171-8.
10. Al-Qadheeb NS, Balk EM, Fraser GL, Skrobik Y, Riker RR, Kress JP, et al. Randomized ICU trials do not demonstrate an association between interventions that reduce delirium duration and short-term mortality: a systematic review and meta-analysis. Crit Care Med. 2014;42(6):1442-54.

For peer review only - http://bmjopen.bmj.com/site/about/guidelines.xhtml

11. Collet MO, Caballero J, Sonneville R, Bozza FA, Nydahl P, Schandl A, et al. Prevalence and risk factors related to haloperidol use for delirium in adult intensive care patients: the multinational AID-ICU inception cohort study. Intensive Care Med. 2018;44(7):1081-9.
12. Jorm AF, Jacomb PA. The Informant Questionnaire on Cognitive Decline in the Elderly (IQCODE): socio-demographic correlates, reliability, validity and some norms. Psychol Med. 1989;19(4):1015-22.
13. Hodgson C, Needham D, Haines K, Bailey M, Ward A, Harrold M, et al. Feasibility and inter-rater reliability of the ICU Mobility Scale. Heart Lung. 2014;43(1):19-24.
14. Richards KC, O'Sullivan PS, Phillips RL. Measurement of sleep in critically ill patients. J Nurs Meas. 2000;8(2):131-44.
15. Simpson GM, Angus JW. A rating scale for extrapyramidal side effects. Acta Psychiatr Scand Suppl. 1970;212:11-9.
16. Jones C, Griffiths RD, Humphris G, Skirrow PM. Memory, delusions, and the development of acute posttraumatic stress disorder-related symptoms after intensive care. Crit Care Med. 2001;29(3):573-80.
17. Breitbart W, Gibson C, Tremblay A. The delirium experience: delirium recall and delirium-related distress in hospitalized patients with cancer, their spouses/caregivers, and their nurses. Psychosomatics. 2002;43(3):183-94.
18. Nasreddine ZS, Phillips NA, Bedirian V, Charbonneau S, Whitehead V, Collin I, et al. The Montreal Cognitive Assessment, MoCA: a brief screening tool for mild cognitive impairment. J Am Geriatr Soc. 2005;53(4):695-9.
19. Bean J. Rey Auditory Verbal Learning Test, Rey AVLT. In: Kreutzer JS, DeLuca J, Caplan B, editors. Encyclopedia of Clinical Neuropsychology. New York, NY: Springer New York; 2011. p. 2174-5.
20. Ledoux K, Vannorsdall TD, Pickett EJ, Bosley LV, Gordon B, Schretlen DJ. Capturing additional information about the organization of entries in the lexicon from verbal fluency productions. J Clin Exp Neuropsychol. 2014;36(2):205-20.
21. Wechsler D. Wechsler adult intelligence scale–Fourth Edition (WAIS–IV). San Antonio, TX: NCS Pearson. 2008;22:498.
22. Reitan RM, Wolfson D. The Halstead–Reitan neuropsychological test battery for adults—Theoretical, methodological, and validational bases. Neuropsychological assessment of neuropsychiatric and neuromedical disorders. 2009;1:36.
23. Roth C. Boston Naming Test. In: Kreutzer JS, DeLuca J, Caplan B, editors. Encyclopedia of Clinical Neuropsychology. New York, NY: Springer New York; 2011. p. 430-3.
24. Zigmond AS, Snaith RP. The hospital anxiety and depression scale. Acta Psychiatr Scand. 1983;67(6):361-70.
25. Ware JE, Jr., Sherbourne CD. The MOS 36-item short-form health survey (SF-36). I. Conceptual framework and item selection. Med Care. 1992;30(6):473-83.
26. Robinson BC. Validation of a Caregiver Strain Index. J Gerontol. 1983;38(3):344-8.
27. Weiss DS, Marmar CR, Wilson JP, Keane TM. Assessing psychological trauma and PTSD. The Impact of Events Scale—Revised. 1997;19:399-411.
28. Girard TD, Exline MC, Carson SS, Hough CL, Rock P, Gong MN, et al. Haloperidol and Ziprasidone for Treatment of Delirium in Critical Illness. The New England journal of medicine. 2018;379(26):2506-16.
29. Nederlandse Vereniging voor Psychiatrie. Richtlijn delirium. Amsterdam: Dries van Ingen, Uitgeverij Boom; 2004. p. 1-127.
30. Barnes TR. The Barnes Akathisia Rating Scale--revisited. J Psychopharmacol. 2003;17(4):365-70.
31. Fongemie JM, Al-Qadheeb NS, Estes NA, 3rd, Roberts RJ, Temtanakitpaisan Y, Ruthazer R, et al. Agreement between ICU clinicians and electrophysiology cardiologists on the decision to initiate a QTc-interval prolonging medication in critically ill patients with potential risk factors for torsade de pointes: a comparative, case-based evaluation. Pharmacotherapy. 2013;33(6):589-97.

For peer review only - http://bmjopen.bmj.com/site/about/guidelines.xhtml

32. Tisdale JE, Jaynes HA, Kingery JR, Overholser BR, Mourad NA, Trujillo TN, et al. Effectiveness of a clinical decision support system for reducing the risk of QT interval prolongation in hospitalized patients. Circ Cardiovasc Qual Outcomes. 2014;7(3):381-90.
33. Trogrlic Z, van der Jagt M, Lingsma H, Gommers D, Ponssen HH, Schoonderbeek JFJ, et al. Improved Guideline Adherence and Reduced Brain Dysfunction After a Multicenter Multifaceted Implementation of ICU Delirium Guidelines in 3,930 Patients. Crit Care Med. 2019.
34. Barr J, Fraser GL, Puntillo K, Ely EW, Gelinas C, Dasta JF, et al. Clinical practice guidelines for the management of pain, agitation, and delirium in adult patients in the intensive care unit. Crit Care Med. 2013;41(1):263-306.

AUTHORS' CONTRIBUTIONS

MJ developed the study protocol, edited and approved the final version, and was responsible for funding and supervising the study coordination. ZT contributed to the protocol development and study implementation. LS is responsible for study coordination. WR assisted with statistical analysis and NH assisted with pharmacological coordination. RO, AS, HP and JD were involved in the study design and protocol development. All authors contributed to the development and refinement of this study protocol. They have read and approved the final version of the protocol.

Acknowledgements

EuRIDICE study group authors:

Local principal investigators: M. van den Boogaard (15), A.J.B.W. Brouwers (7), J.A. Lens (9), B.J.M. van der Meer (8) , H. Ponssen (4), F.J. Schoonderbeek (10), K.S. Simons (16).

Local research nurses: E. Berger (7), A. Bouman (16), M. Campo (8), D. van Duijn (1), H. Embden – van Donk (10), D. van de Graaf (9), E. Hoogendoorn (4), P. Ormskerk (1), N. Roovers (15), E. Toscano (8), A. Vileito (1), T. van Zuylen (16).

All involved local principal investigators (MB, AB, BM, JL, EK, FS, KS) and research nurses (EB, AB, DD, HE, DG, PO, NR, AV, MC, ET, EH, TZ) have facilitated visits at their site, will be involved in executing the protocol at their sites and will be responsible for patient recruitment and data collection in their hospitals, along with follow up of study patients.

For peer review only - http://bmjopen.bmj.com/site/about/guidelines.xhtml

Other members involved: C. Exler (11), E. van den Berg (12), J. van Meeteren (13), M. Koopmanschap (14).

Patient representatives: I. Nutma and E. Kuijper.

1) Erasmus MC-University Medical Center, Department of Intensive Care Adults, Rotterdam, The Netherlands, Netherlands

4) Albert Schweitzer Hospital, Department of Intensive Care, Dordrecht, Netherlands

7) Franciscus Gasthuis, Department of Intensive Care, Rotterdam, Netherlands

8) Maasstad Hospital, Department of Intensive Care, Rotterdam, Netherlands

9) IJsselland Hospital, Department of Intensive Care, Capelle aan den IJssel, Netherlands

10) Ikazia Hospital, Department of Intensive Care, Rotterdam, Netherlands

11) Erasmus MC-University Medical Center, Department of Pharmacy, Rotterdam, The Netherlands, Netherlands

12) Erasmus MC-University Medical Center, Department of Neuropsychology, Rotterdam, The Netherlands, Netherlands

13) Erasmus MC-University Medical Center, Department of Rehabilitation Medicine, Rotterdam, The Netherlands, Netherlands

14) Erasmus School of Health Policy & Management, Department of Health Economics and HTA, Rotterdam, The Netherlands, Netherlands

15) Radboud University Medical Center, Department of Intensive Care Medicine, Nijmegen, The Netherlands

16) Jeroen Bosch Hospital, Department of Intensive Care Medicine, 's-Hertogenbosch, The Netherlands.

FUNDING STATEMENT

A grant has been provided by ZonMw – The Netherlands Organisation for Health Research and Organisation. ZonMw project number: 848041001. This funding source had no role in

For peer review only

For peer review only - http://bmjopen.bmj.com/site/about/guidelines.xhtml

the design of this study and will not have any role during its execution, analyses, interpretation of the data, or decision to submit results.

COMPETING INTERESTS STATEMENT

The authors declare that they have no competing interests.

LIST OF ABBREVIATIONS

**ABR**	**ABR form, General Assessment and Registration form, is the application form that is required for submission to the accredited Ethics Committee (In Dutch, ABR = Algemene Beoordeling en Registratie)**
**AE**	**Adverse Event**
**AR**	**Adverse Reaction**
**CA**	**Competent Authority**
**CAM-ICU**	**Confusion Assessment Method for the ICU**
**CCMO**	**Central Committee on Research Involving Human Subjects; in Dutch: Centrale Commissie Mensgebonden Onderzoek**
**CV**	**Curriculum Vitae**
**DSMB**	**Data Safety Monitoring Board**
**EU**	**European Union**
**EudraCT**	**European drug regulatory affairs Clinical Trials**
**EKG**	**Electrocardiography**
**GCP**	**Good Clinical Practice**
**IB**	**Investigator's Brochure**
**IC**	**Informed Consent**
**ICDSC**	**Intensive Care Delirium Screening Checklist**
**ICU**	**Intensive Care Unit**
**IMP**	**Investigational Medicinal Product**
**IMPD**	**Investigational Medicinal Product Dossier**
**METC**	**Medical research ethics committee (MREC); in Dutch: medisch ethische toetsing commissie (METC)**
**PAD**	**Pain, agitation and delirium**
**RCT**	**Randomized Controlled Trial**
**(S)AE**	**(Serious) Adverse Event**
**S.O.P.**	**Standard Operating Procedure**

For peer review only

SPC **Summary of Product Characteristics (in Dutch: officiële productinfomatie IB1-tekst)**

Sponsor **The sponsor is the party that commissions the organisation or performance of the research, for example a pharmaceutical company, academic hospital, scientific organisation or investigator. A party that provides funding for a study but does not commission it is not regarded as the sponsor, but referred to as a subsidising party.**

SUSAR **Suspected Unexpected Serious Adverse Reaction**

TdP **Torsade de Pointes**

Wbp **Personal Data Protection Act (in Dutch: Wet Bescherming Persoonsgevens)**

WMO **Medical Research Involving Human Subjects Act (in Dutch: Wet Medisch-wetenschappelijk Onderzoek met Mensen)**

Table 1. Overview of timing of assessments, including required time investment per visit/questionnaire.

**Moment (months)**	**Neurocognitive tests**	**Patient and family experiences (time in min.)**	**Functional outcomes (SF-36)**	**Cost effectivity questionnaires (EQ-5D-5L, iMTA MCQ, iMTA PCQ)**	**Other**
**Enrolment**					Informed consent, IQCODE-N, pregnancy test (if applicable), EKG.
**ICU study period (3x / day)**					CAM-ICU / ICDSC, RASS
**ICU study period (once daily)**					IMS, RCSQ. Only when on study medication:

For peer review only

					EKG, Simpson Angus Scale.
**0 (discharge from hospital)**		Patient: ICU-MT (15) + DEQ (15) Family: DEQ (2)			
**1**				30 min.	
**3**	45-60 min.	Patient: IES-R (5) + ICU-MT (15) + DEQ (15) Family: IES-R (5) + CSI (5) + DEQ (2)	10 min.	30 min.	
**6**				30 min.	
**12**	45-60 min.		10 min.	30 min.	

IQCODE-N = Informant Questionnaire on Cognitive Decline in the Elderly – Dutch version

EKG = Electrocardiography

Neurocognitive tests: Montreal Cognitive Assessment (MOCA), Rey Auditory Verbal Learning Test, Semantic fluency, Digit Span (WAIS-IV), Trailmaking tests A and B, Boston naming Test (short version), Hospital Anxiety and Depression Scale (HADS)

IMS = ICU Mobility Scale, measures mobility during ICU admission

RCSQ = Richards-Campbell Sleep Questionnaire, measures quality of sleep

Simpson Angus Scale = measures muscle rigidity and other associated movements disorders

ICU-MT= ICU-Memory Tool, assesses the experience and memories of ICU admission

DEQ= Delirium Experience Questionnaire, measures experiences linked to delirium

IES-R = Impact of Event Scale Revised, assesses distress linked to a traumatic experience (i.e. experiencing delirium)

CSI = Caregiver Strain Index, assesses the strain experienced by the caregiver

For peer review only - http://bmjopen.bmj.com/site/about/guidelines.xhtml

SF-36 = Short Form-36, measures the health-related quality of life

EQ-5D-5L = assesses the general health status

iMTA MCQ = instituut Beleid & Management Gezondheidszorg Medical Consumption Questionnaire (health care use)

iMTA PCQ = instituut Beleid & Management Gezondheidszorg Productivity Cost Questionnaire (productivity costs)

With the exception of the neurocognitive tests, all above mentioned tools are questionnaires that can be administered at home. Real life visits only need to be paid in order to perform the neurocognitive tests.

For peer review only

Appendix 1: Participating hospitals

Erasmus MC Rotterdam

Albert Schweizer Hospital Dordrecht

Maasstad Hospital Rotterdam

IJsselland Hospital Capelle aan den IJssel

Ikazia Hospital Rotterdam

Franciscus Gasthuis Rotterdam

As of July 2019 two additional ICUs have started recruitment:

Jeroen Bosch Hospital, 's-Hertogenbosch

Radboud University Medical Center, Nijmegen

For peer review only

Appendix 2: Economic evaluation

ECONOMIC EVALUATION

General considerations

The primary economic analysis will be a trial-based **cost-utility analysis** from a societal and a healthcare perspective. This analysis will be performed according to the Dutch guidelines (1, 2). The time horizon will be 12 months after randomisation, in order to take all relevant costs and effects regarding the treatment procedure into account. Additionally, a **cost-effectiveness analysis** performed from a societal and health care perspective will be conducted, using delirium-free and coma-free days as outcomes.

If a difference in quality of life is observed at the end of the follow-up period, we will also perform a **model-based extrapolation** of costs and health benefits up to 5 years, exploring the following scenarios: (1) health benefit remain constant after the follow-up period, (2) health benefits are gradually phased out over the course of the modelling time, (3) health benefits are gradually phased out over the course of the modelling time over the first year after follow-up, (4) health benefits abruptly disappear after the follow-up period, but costs remain until the end of the modelling period.

If treatment with haloperidol leads to better health outcomes at higher costs, or if it leads to worse health outcomes and cost savings, incremental cost-utility and incremental cost-effectiveness ratios will be calculated. These express the additional costs per unit of health gain (QALYs, delirium-free days, coma-free days) or the savings per unit of health forgone. The uncertainty around the estimates will be addressed using bootstrapping for the analysis of costs and effects in the first 12 months, and using probabilistic sensitivity analysis in the extrapolation model.

Cost analysis

Healthcare costs will be calculated based on patient-level data on health-care utilization, which will be collected from hospital databases and questionnaires, to be filled out at regular intervals by patients and/or informal caregivers. Cost categories include medication, screenings, inpatient days, contacts with healthcare providers (GP, outpatient visits, and therapists). The questionnaire will also contain questions about absence from paid work by the patient and informal caregivers.

Costs will be calculated by multiplying resource utilization with the cost per unit of resource. Some unit costs will be taken from the 2016 Dutch Manual for Costing Studies(3), but the costs of inpatient days will be assessed following the micro-costing method, which is based on comprehensive ‘bottom-up’ analyses of the activities of staff and other resources that are used

during those days. Medication prices will be based on the official list prices, including value added tax and increased by a standard prescription reimbursement for the pharmacist. The cost of production loss will be calculated according to the Friction Cost Approach.

Patient outcome analysis

The primary outcome measure in the economic evaluation is the difference in QALYs. The secondary effects are the delirium-free and coma-free days after treatment with haloperidol or placebo. As measuring QALYs in adult critically ill patients is not feasible at baseline, it is not possible to estimate the average number of QALYs for each treatment group. However, assuming that there is no difference at randomisation, it is possible to analyse the difference in quality of life at subsequent measurements in a multilevel regression model. This will enable us to calculate a difference in QALYs between the treatment groups over the total follow-up period, using linear intrapolation. HRQoL will be measured on t=1, 3, 6 and 12 months after randomization using the EQ-5D-5L instrument.

References:

1. Hakkaart-van Roijen L., Tan SS., Bouwmans CAM. Handleiding voor kostenonderzoek, methoden en standaard kostprijzen voor economische evaluaties in de gezondheidszorg. College voor zorgverzekeringen, Geactualiseerde versie 2010.
2. Zorginstituut Nederland. Richtlijn voor het uitvoeren van economische evaluaties in de gezondheidszorg. 2015.
3. Hakkaart-van Roijen L., van der Linden N., Bouwmans CAM., Kanters T., Tan SS. Costing manual: Methodology of costing research and reference prices for economic evaluations in healthcare. 2015.

For peer review only

***Appendix 3:* Haloperidol SPC**

See this weblink: https://db.cbg-meb.nl/IB-teksten/h03185.pdf

For peer review only

Appendix 4: (Semi-) structured review of literature on efficacy and adverse events of haloperidol for delirium in adult critically ill patients

1. Haloperidol as a treatment for ICU delirium

Systematic review of randomised placebo-controlled trials assessing haloperidol for treatment of ICU delirium

Method: A biomedical Information Specialist (BIS) of the Erasmus Medical Center library performed a systematic search aimed at controlled studies on haloperidol for ICU delirium combining the subjects: delirium, ICU and haloperidol, or equivalent terms (see: Appendix for details). No distinction was made in the search between treatment or prevention trials.

Review: Since focus of the EuRIDICE study is on a haloperidol versus placebo comparison, the study selection for this summary is also focused on placebo-controlled haloperidol trials for the treatment of ICU delirium. Systematic reviews from the systematic search are used as a crosscheck to confirm completeness or provide additional insights. The search (total of yielded only 1 study. The MIND trial (2010) was a randomised placebo controlled feasibility, efficacy and safety trial of antipsychotics for ICU delirium in adult mechanically ventilated medical and surgical patients (1). It included three treatment arms (haloperidol, n=35; ziprasidone, n=30 and placebo, n=36) and used a well thought out design (excluding demented patients with a validated tool for cognitive dysfunction, using CAM-ICU as a validated screening tool, a clear protocol with regard to QTc prolongation and study drug dosing, measuring extrapyramidal symptoms with a validated scale and with number of days alive without delirium and coma as the primary outcome (indicating total burden of brain dysfunction, since only assessing delirium days may result in increased coma days and less delirium days being regarded as a – false – improvement). The study used oral haloperidol, no clear sedation protocol aimed at light sedation and crossover antipsychotics were allowed but discouraged. No clear differences were found in the three groups with regard to the primary outcome. Mean haloperidol dose was 15 mg a day but QTc prolongation and extrapyramidal symptoms did not differ between treatment groups. Other medications in this small trial did not differ between groups (propofol, opiates, benzodiazepines). It was concluded that a larger trial would be safe and feasible.

Overview of most recent guidelines' statements on haloperidol as treatment for ICU delirium

Method: Pubmed search on published guidelines including ICU delirium and containing information on

For peer review only

haloperidol. Search terms: guideline, delirium, ICU.

Review: Three recent guidelines were retrieved (2-4). In a Danish guideline (2015) no evidence is stated for pharmacological management(2). A German guideline (2015) advocates symptom-based therapy when delirium screening is positive with haloperidol as a first choice in case of delirium associated with psychotic symptoms only. The "Clinical practice guidelines for the management of pain, agitation, and delirium in adult patients in the intensive care unit" (2013)(3) advocate avoiding 'antipsychotics' when risk of torsades de pointes or is present or either baseline QT prolongation or concomitant QT prolonging medication is used. It states that there is no evidence that haloperidol decrease delirium duration, which was perceived as the most relevant issue to address with regard to haloperidol treatment of ICU delirium.

Cochrane review(s)

Method: Search on Cochrane (http://www.cochranelibrary.com) for reviews with search term: 'delirium', does not elicit any results pertaining to pharmacological treatment of delirium nor haloperidol.

Review: no Cochrane reviews exist on (ICU) delirium and it's pharmacological management.

On-going trials

Method: A search for 'haloperidol' and 'delirium' in the following online trial databases (and including ICU patients); www.trialregister.nl (0 trials); www.clinicaltrials.gov (4 trials).

Review: Four trials were retrieved from www.clinicaltrials.gov. One trial ('Haloquet') was not a truly placebo controlled trial because haloperidol was allowed ('as needed') in the placebo group and was last updated in 2013 but not published. It consisted of three treatments arms (also quetiapine) and aimed to include a total of 45 patients (and should thus be considered a pilot trial and not an efficacy trial). A second trial enrolled 40 patients and was completed in 2011 but not published. A third trial was a phase-2 safety/efficacy study enrolling 20 patients, last updated in 2007 and not published. The fourth trial ('The modifying the impact of ICU-associated neurological dysfunction-USA [MIND-USA] study') is currently recruiting (last verified May 2016 on September 14th). It is a multi-center double blind placebo-controlled trial aiming to enrol 561 patients in three treatment arms: haloperidol, ziprasidone and placebo, by the same research group that did the MIND trial. It includes cognitive and

For peer review only

psychological follow-up at 12 months and is estimated to be completed July 2019. Maximum dose of haloperidol amounts to 10 mg IV q12 hours. Trial design is similar to the EuRIDICE trial, except for the patient experiences and perspective, and the fact that only patients on mechanical ventilation or in shock are included (i.e. the sickest ICU patients). The study protocol has not been published in a peerreviewed journal.

2. Haloperidol to prevent ICU delirium

Systematic review of randomised placebo-controlled trials assessing haloperidol for prevention of ICU delirium; including information from guidelines and Cochrane reviews

Method: A biomedical Information Specialist (BIS) of the Erasmus Medical Center library performed a systematic search aimed at controlled studies on haloperidol for ICU delirium combining the subjects: delirium, ICU and haloperidol, or equivalent terms (see: Appendix for details).

Review: the focus of this section is on randomised placebo-controlled prevention trials of haloperidol for ICU delirium. Three trials were retrieved. One trial included post-operative generally non-critically ill patients (5) and was not further considered for this review. The Hope-ICU trial (2013)(6) was a prophylactic study of haloperidol (2.5mg IV q8h, n=71) versus placebo (n=70) in adult mechanically ventilated ICU patients. The primary end-point of delirium (assessed with CAM-ICU) and coma free days did not differ between groups (5 days in both), but there was a 21% crossover rate with haloperidol in the placebo group. Secondary clinical endpoints such as length of stay at ICU or mortality did not differ but the trial was not powered on these outcomes. Another trial (2016)(7) including mechanically ventilated patients (n=68) with 'subsyndromal' delirium (=an Intensive Care Delirium Screening Checklist [ICDSC] score of 1-3 on a scale of 8, where 4 or more is compatible with delirium) used haloperidol 1mg IV q6h but did not find lower rate of progression to full delirium.

3. Haloperidol: adverse events versus treatment effects in the few available trials

The adverse events associated with haloperidol in the three aforementioned (small) trials (one treatment and two prevention trials) did not include QTc prolongation (with a threshold of >500 ms). In the Hope-ICU trial more opiates and sedatives were administered in the placebo-group but alfa-2

For peer review only

agonists were not clearly protocolled, more agitation was present and 26% versus 11% antipsychotics' use in the placebo group. The subsyndromal delirium trial similarly found more agitation in the placebo group.

4. Healthcare perspective

A cost-effectiveness analysis of the Hope-ICU trial found that delirium increased cognitive dysfunction at 6 months and reduced quality of life, suggestive of potential cost-effectiveness of haloperidol (8).

5. Added value of the EuRIDICE trial

Based on this review of available pertinent literature after a thorough BIS-supported systematic search, the proposed trial in this grant application is expected to have important potential additional value:

The indication of haloperidol for ICU delirium will be delineated more clearly by this trial: does it decrease ICU brain dysfunction, associated long-term cognitive, functional and psychological outcomes? Is the intervention cost-effective? Are adverse events associated with haloperidol indeed concerning or actually negligible? Or: has haloperidol become obsolete, now that alternatives have been incorporated into clinical practice, mainly the atypical antipsychotics and alpha-2 agonists (dexmedetomidine and clonidine)? The EuRIDICE trial has a very strong potential to answers all of these questions.

A similar trial as EuRIDICE in the United States is on-going. However, US-based delirium research may not necessarily translate to European/Dutch settings as has been shown before (9), which justifies performing a second large multicentre clinical trial. Moreover, evidence on the pharmacological treatment of delirium is needed because of the lack of trials to date, and the level of evidence and generalizability of the efficacy findings for haloperidol will increase with a second trial. Third, cost-effectiveness of the intervention will be assessed from a healthcare and societal perspective and family and patient experiences will be investigated as important secondary outcomes. Further, we aim to include all critically ill patients, and not just the sickest, i.e. those on mechanical ventilation or in shock.

Existing guidelines and systematic reviews will have to be adapted on the basis of the results this proposed trial.

For peer review only

Acknowledgements:

Gerdien B. de Jonge (MSc), biomedical information specialist, Medical Library, Erasmus MC, is kindly acknowledged for her help in assembling the databases for the systematic review.

References

1. Girard TD, Pandharipande PP, Carson SS, Schmidt GA, Wright PE, Canonico AE, et al. Feasibility, efficacy, and safety of antipsychotics for intensive care unit delirium: The mind randomized, placebo-controlled trial. Crit Care Med. 2010;38(2):428-37.
2. Fonsmark L, Hein L, Nibroe H, Bundgaard H, de Haas I, Iversen S, et al. Danish national sedation strategy. Targeted therapy of discomfort associated with critical illness. Danish society of intensive care medicine (dsit) and the danish society of anesthesiology and intensive care medicine (dasaim). Dan Med J. 2015;62(4):C5052.
3. Barr J, Fraser GL, Puntillo K, Ely EW, Gelinas C, Dasta JF, et al. Clinical practice guidelines for the management of pain, agitation, and delirium in adult patients in the intensive care unit. Crit Care Med. 2013;41(1):263-306.
4. D. A. S. Taskforce, Baron R, Binder A, Biniek R, Braune S, Buerkle H, et al. Evidence and consensus based guideline for the management of delirium, analgesia, and sedation in intensive care medicine. Revision 2015 (das-guideline 2015) - short version. Ger Med Sci. 2015;13:Doc19.
5. Wang W, Li HL, Wang DX, Zhu X, Li SL, Yao GQ, et al. Haloperidol prophylaxis decreases delirium incidence in elderly patients after noncardiac surgery: A randomized controlled trial*. Crit Care Med. 2012;40(3):731-9.
6. Page VJ, Ely EW, Gates S, Zhao XB, Alce T, Shintani A, et al. Effect of intravenous haloperidol on the duration of delirium and coma in critically ill patients (hope-icu): A randomised, double-blind, placebo-controlled trial. Lancet Respir Med. 2013;1(7):515-23.
7. Al-Qadheeb NS, Skrobik Y, Schumaker G, Pacheco MN, Roberts RJ, Ruthazer RR, et al. Preventing icu subsyndromal delirium conversion to delirium with low-dose iv haloperidol: A double-blind, placebo-controlled pilot study. Crit Care Med. 2016;44(3):583-91.
8. Joachim M, Daniel FM, Annalisa C, Timothy A, Xiao BZ, Eugene WE, et al. Health evaluation and cost-effectiveness analysis from a randomized trial of haloperidol in the management of delirium in the critically ill (hope-icu trial). C24 sedation, delirium, and neurocognitive dysfunction in critical care. p. A4022-A.
9. van Eijk MM, van den Boogaard M, van Marum RJ, Benner P, Eikelenboom P, Honing ML, et al. Routine use of the confusion assessment method for the intensive care unit a multicenter study. Am J Respir Crit Care Med. 2011;184(3):340-4.

Appendix 5: Drug Accountability

The study drug will be obtained from the hospital pharmacy of each participating ICU.

The research nurse of each participating ICU will record the number of the box with study drug for each patient in the CRF.

The research nurse of each participating ICU is responsible for retrieving the boxes with study drug. The amount of vials in the boxes will be counted for each patient and will be noted in the CRF. The research nurse will return unused drug to the hospital pharmacy. The hospital pharmacy will destroy the vials with study drug and will also record this (double administration).

The pharmacist or another appropriate individual who is designated should maintain records of the product’s delivery to the trial site, the inventory at the site, the use by each patient, problems and irregularities during injection, the maintenance of the blinding, and the return to the pharmacy of unused product(s). These records should include dates, quantities, batch/serial numbers, expiration dates (if applicable), and the unique code numbers assigned to the investigational product(s) and trial patients (if applicable). Investigators should maintain records that document adequately that the patients were provided the doses specified by the protocol and reconcile all investigational product(s).

For peer review only

Appendix 6: Unblinding Procedure

The study treatment will be unblinded after Database Lock. While the safety of patients should always take priority, maintenance of blinding is crucial to the integrity of a double-blind trial. Before this planned unblinding, the blinding for a specific patient should only be broken when information about the patient's protocol treatment is considered necessary to manage Serious Adverse Events (emergency unblinding). Unblinding procedures should preferably be initiated only after consultation of the principal investigator/coordinating investigator or his/her representative. To initiate an emergency unblinding the pharmacy in charge of the randomisation list should be contacted. Breaking the blinding on a patient will be logged and reported to the coordinating Investigator within 24 hours following the unblinding procedure, using the Emergency Unblinding Form. It is considered a major protocol violation, after which the patient goes off protocol treatment (if applicable).

For peer review only - http://bmjopen.bmj.com/site/about/guidelines.xhtml

Reporting checklist for protocol of a clinical trial.

Based on the SPIRIT guidelines.

Instructions to authors

Complete this checklist by entering the page numbers from your manuscript where readers will find each of the items listed below.

Your article may not currently address all the items on the checklist. Please modify your text to include the missing information. If you are certain that an item does not apply, please write "n/a" and provide a short explanation.

Upload your completed checklist as an extra file when you submit to a journal.

In your methods section, say that you used the SPIRITreporting guidelines, and cite them as:

Chan A-W, Tetzlaff JM, Altman DG, Laupacis A, Gøtzsche PC, Krleža-Jerić K, Hróbjartsson A, Mann H, Dickersin K, Berlin J, Doré C, Parulekar W, Summerskill W, Groves T, Schulz K, Sox H, Rockhold FW, Rennie D, Moher D. SPIRIT 2013 Statement: Defining standard protocol items for clinical trials. Ann Intern Med. 2013;158(3):200-207

		Reporting Item	Page Number
**Administrative information**			
Title	#1	Descriptive title identifying the study design, population, interventions, and, if applicable, trial acronym	1
Trial registration	#2a	Trial identifier and registry name. If not yet registered, name of intended registry	3
Trial registration: data set	#2b	All items from the World Health Organization Trial Registration Data Set	NA
Protocol version	#3	Date and version identifier	2
Funding	#4	Sources and types of financial, material, and other support	22

For peer review only

Roles and responsibilities: contributorship	#5a	Names, affiliations, and roles of protocol contributors	1, 20
Roles and responsibilities: sponsor contact information	#5b	Name and contact information for the trial sponsor	1,2
Roles and responsibilities: sponsor and funder	#5c	Role of study sponsor and funders, if any, in study design; collection, management, analysis, and interpretation of data; writing of the report; and the decision to submit the report for publication, including whether they will have ultimate authority over any of these activities	22
Roles and responsibilities: committees	#5d	Composition, roles, and responsibilities of the coordinating centre, steering committee, endpoint adjudication committee, data management team, and other individuals or groups overseeing the trial, if applicable (see Item 21a for data monitoring committee)	14
**Introduction**			
Background and rationale	#6a	Description of research question and justification for undertaking the trial, including summary of relevant studies (published and unpublished) examining benefits and harms for each intervention	3-5
Background and rationale: choice of comparators	#6b	Explanation for choice of comparators	3-5
Objectives	#7	Specific objectives or hypotheses	4
Trial design	#8	Description of trial design including type of trial (eg, parallel group, crossover, factorial, single group), allocation ratio, and framework (eg, superiority, equivalence, non-inferiority, exploratory)	4
**Methods: Participants,**			

For peer review only

**interventions, and outcomes**			
Study setting	#9	Description of study settings (eg, community clinic, academic hospital) and list of countries where data will be collected. Reference to where list of study sites can be obtained	5
Eligibility criteria	#10	Inclusion and exclusion criteria for participants. If applicable, eligibility criteria for study centres and individuals who will perform the interventions (eg, surgeons, psychotherapists)	5-6
Interventions: description	#11a	Interventions for each group with sufficient detail to allow replication, including how and when they will be administered	9-10
Interventions: modifications	#11b	Criteria for discontinuing or modifying allocated interventions for a given trial participant (eg, drug dose change in response to harms, participant request, or improving / worsening disease)	9-12
Interventions: adherance	#11c	Strategies to improve adherence to intervention protocols, and any procedures for monitoring adherence (eg, drug tablet return; laboratory tests)	11
Interventions: concomitant care	#11d	Relevant concomitant care and interventions that are permitted or prohibited during the trial	11-12
Outcomes	#12	Primary, secondary, and other outcomes, including the specific measurement variable (eg, systolic blood pressure), analysis metric (eg, change from baseline, final value, time to event), method of aggregation (eg, median, proportion), and time point for each outcome. Explanation of the clinical relevance of chosen efficacy and harm outcomes is strongly recommended	7-9
Participant timeline	#13	Time schedule of enrolment, interventions (including any run-ins and washouts), assessments, and visits for participants. A schematic diagram is highly recommended (see Figure)	7-9
Sample size	#14	Estimated number of participants needed to achieve study objectives and how it was determined, including	17

For peer review only

		clinical and statistical assumptions supporting any sample size calculations	
Recruitment	#15	Strategies for achieving adequate participant enrolment to reach target sample size	12
**Methods: Assignment of interventions (for controlled trials)**			
Allocation: sequence generation	#16a	Method of generating the allocation sequence (eg, computer-generated random numbers), and list of any factors for stratification. To reduce predictability of a random sequence, details of any planned restriction (eg, blocking) should be provided in a separate document that is unavailable to those who enrol participants or assign interventions	12-13
Allocation concealment mechanism	#16b	Mechanism of implementing the allocation sequence (eg, central telephone; sequentially numbered, opaque, sealed envelopes), describing any steps to conceal the sequence until interventions are assigned	12-13
Allocation: implementation	#16c	Who will generate the allocation sequence, who will enrol participants, and who will assign participants to interventions	12-13
Blinding (masking)	#17a	Who will be blinded after assignment to interventions (eg, trial participants, care providers, outcome assessors, data analysts), and how	12-13
Blinding (masking): emergency unblinding	#17b	If blinded, circumstances under which unblinding is permissible, and procedure for revealing a participant's allocated intervention during the trial	12-13
**Methods: Data collection, management, and analysis**			
Data collection plan	#18a	Plans for assessment and collection of outcome, baseline, and other trial data, including any related processes to promote data quality (eg, duplicate	7-9

For peer review only

		measurements, training of assessors) and a description of study instruments (eg, questionnaires, laboratory tests) along with their reliability and validity, if known. Reference to where data collection forms can be found, if not in the protocol	
Data collection plan: retention	#18b	Plans to promote participant retention and complete follow-up, including list of any outcome data to be collected for participants who discontinue or deviate from intervention protocols	13-14
Data management	#19	Plans for data entry, coding, security, and storage, including any related processes to promote data quality (eg, double data entry; range checks for data values). Reference to where details of data management procedures can be found, if not in the protocol	12, 15
Statistics: outcomes	#20a	Statistical methods for analysing primary and secondary outcomes. Reference to where other details of the statistical analysis plan can be found, if not in the protocol	16
Statistics: additional analyses	#20b	Methods for any additional analyses (eg, subgroup and adjusted analyses)	16-17
Statistics: analysis population and missing data	#20c	Definition of analysis population relating to protocol non-adherence (eg, as randomised analysis), and any statistical methods to handle missing data (eg, multiple imputation)	14
**Methods: Monitoring**			
Data monitoring: formal committee	#21a	Composition of data monitoring committee (DMC); summary of its role and reporting structure; statement of whether it is independent from the sponsor and competing interests; and reference to where further details about its charter can be found, if not in the protocol. Alternatively, an explanation of why a DMC is not needed	14
Data monitoring: interim analysis	#21b	Description of any interim analyses and stopping guidelines, including who will have access to these	16-17

For peer review only

		interim results and make the final decision to terminate the trial	
Harms	#22	Plans for collecting, assessing, reporting, and managing solicited and spontaneously reported adverse events and other unintended effects of trial interventions or trial conduct	15-16
Auditing	#23	Frequency and procedures for auditing trial conduct, if any, and whether the process will be independent from investigators and the sponsor	NA
**Ethics and dissemination**			
Research ethics approval	#24	Plans for seeking research ethics committee / institutional review board (REC / IRB) approval	17
Protocol amendments	#25	Plans for communicating important protocol modifications (eg, changes to eligibility criteria, outcomes, analyses) to relevant parties (eg, investigators, REC / IRBs, trial participants, trial registries, journals, regulators)	17
Consent or assent	#26a	Who will obtain informed consent or assent from potential trial participants or authorised surrogates, and how (see Item 32)	18
Consent or assent: ancillary studies	#26b	Additional consent provisions for collection and use of participant data and biological specimens in ancillary studies, if applicable	NA
Confidentiality	#27	How personal information about potential and enrolled participants will be collected, shared, and maintained in order to protect confidentiality before, during, and after the trial	18
Declaration of interests	#28	Financial and other competing interests for principal investigators for the overall trial and each study site	22
Data access	#29	Statement of who will have access to the final trial dataset, and disclosure of contractual agreements that limit such access for investigators	-

For peer review only

Ancillary and post trial care	#30	Provisions, if any, for ancillary and post-trial care, and for compensation to those who suffer harm from trial participation	NA
Dissemination policy: trial results	#31a	Plans for investigators and sponsor to communicate trial results to participants, healthcare professionals, the public, and other relevant groups (eg, via publication, reporting in results databases, or other data sharing arrangements), including any publication restrictions	3
Dissemination policy: authorship	#31b	Authorship eligibility guidelines and any intended use of professional writers	-
Dissemination policy: reproducible research	#31c	Plans, if any, for granting public access to the full protocol, participant-level dataset, and statistical code	NA
**Appendices**			
Informed consent materials	#32	Model consent form and other related documentation given to participants and authorised surrogates	-
Biological specimens	#33	Plans for collection, laboratory evaluation, and storage of biological specimens for genetic or molecular analysis in the current trial and for future use in ancillary studies, if applicable	NA

None The SPIRIT checklist is distributed under the terms of the Creative Commons Attribution License CC-BY-ND 3.0. This checklist can be completed online using https://www.goodreports.org/, a tool made by the EQUATOR Network in collaboration with Penelope.ai

For peer review only

BMJ Open

Efficacy of halopeRIdol to decrease the burden of Delirium In adult Critically ill patiEnts (EuRIDICE): study protocol for a prospective randomised multi-center double-blind placebo-controlled clinical trial

Journal:	*BMJ Open*
Manuscript ID	bmjopen-2019-036735.R1
Article Type:	Protocol
Date Submitted by the Author:	16-Apr-2020
Complete List of Authors:	Smit, Lisa; Erasmus Medical Center, Department of Intensive Care Adults<br>Trogrlić, Zoran; Erasmus Medical Center, Department of Intensive Care Adults<br>Devlin , John; Northeastern University, Bouve College of Health Sciences; Tufts Medical Center, Division of Pulmonary, Critical Care and Sleep Medicine, Department of Pharmacy and Health Systems Sciences<br>Osse, Robert-Jan; Erasmus Medical Center, Department of Psychiatry<br>Ponssen, Huibert; Albert Schweitzer Hospital Location Dordwijk, Department of Intensive Care<br>Slooter, Arjen ; University Medical Centre Utrecht Brain Centre, Department of Intensive Care Medicine and UMC Utrecht Brain Center<br>Hunfeld, Nicole; Erasmus Medical Center, Department of Pharmacy and Department of Intensive Care<br>Rietdijk, Wim; Erasmus Medical Center, Department of Intensive Care Adults<br>Gommers, Diederik; Erasmus Medical Center, Department of Intensive Care Adults<br>van der Jagt, Mathieu; Erasmus Medical Center, Department of Intensive Care Adults
**Primary Subject Heading**:	Intensive care
Secondary Subject Heading:	Intensive care
Keywords:	Delirium & cognitive disorders < PSYCHIATRY, INTENSIVE & CRITICAL CARE, Adult intensive & critical care < ANAESTHETICS

SCHOLARONE™
Manuscripts

BMJ

I, the Submitting Author has the right to grant and does grant on behalf of all authors of the Work (as defined in the below author licence), an exclusive licence and/or a non-exclusive licence for contributions from authors who are: i) UK Crown employees; ii) where BMJ has agreed a CC-BY licence shall apply, and/or iii) in accordance with the terms applicable for US Federal Government officers or employees acting as part of their official duties; on a worldwide, perpetual, irrevocable, royalty-free basis to BMJ Publishing Group Ltd ("BMJ") its licensees and where the relevant Journal is co-owned by BMJ to the co-owners of the Journal, to publish the Work in this journal and any other BMJ products and to exploit all rights, as set out in our licence.

The Submitting Author accepts and understands that any supply made under these terms is made by BMJ to the Submitting Author unless you are acting as an employee on behalf of your employer or a postgraduate student of an affiliated institution which is paying any applicable article publishing charge ("APC") for Open Access articles. Where the Submitting Author wishes to make the Work available on an Open Access basis (and intends to pay the relevant APC), the terms of reuse of such Open Access shall be governed by a Creative Commons licence – details of these licences and which Creative Commons licence will apply to this Work are set out in our licence referred to above.

Other than as permitted in any relevant BMJ Author's Self Archiving Policies, I confirm this Work has not been accepted for publication elsewhere, is not being considered for publication elsewhere and does not duplicate material already published. I confirm all authors consent to publication of this Work and authorise the granting of this licence.

For peer review only

Efficacy of halopeRIdol to decrease the burden of Delirium In adult Critically ill patiEnts (EuRIDICE): study protocol for a prospective randomised multi-center double-blind placebo-controlled clinical trial

Lisa Smit – l.smit@erasmusmc.nl (1), Zoran Trogrlic – z.trogrlic@erasmusmc.nl (1), John W. Devlin - j.devlin@northeastern.edu (2), Robert Jan Osse - r.osse@erasmusmc.nl (3), Huibert H. Ponssen - H.H.Ponssen@asz.nl (4), Arjen J.C. Slooter - A.Slooter-3@umcutrecht.nl (5), Nicole G.M. Hunfeld - n.hunfeld@erasmusmc.nl (1, 6), Wim J.R. Rietdijk - w.rietdijk@erasmusmc.nl (1), Diederik Gommers - d.gommers@erasmusmc.nl (1), Mathieu van der Jagt – m.vanderjagt@erasmusmc.nl* (1), on behalf of the EuRIDICE study group.

1) Erasmus MC-University Medical Center, Department of Intensive Care Adults, Rotterdam, The Netherlands, Netherlands

2) Bouve College of Health Sciences, Northeastern University; Division of Pulmonary, Critical Care and Sleep Medicine, Department of Pharmacy and Health Systems Sciences, Boston, United States

3) Erasmus MC-University Medical Center, Department of Psychiatry, Rotterdam, The Netherlands, Netherlands

4) Albert Schweitzer Hospital, Department of Intensive Care, Dordrecht, Netherlands

5) University Medical Center Utrecht, Utrecht University, Department of Intensive Care Medicine and UMC Utrecht Brain Center, Utrecht, The Netherlands

6) Erasmus MC-University Medical Center, Departments of Intensive Care Adults and Pharmacy, Rotterdam, The Netherlands, Netherlands

*Corresponding author:

Department of Intensive Care, room Ne-415

Erasmus MC

For peer review only

PO BOX 2040
3000 CA Rotterdam
E-mail: m.vanderjagt@erasmusmc.nl
Telephone number: +31107040704

Protocol version: November 16th 2017, Version 2

Word count: 4326 words

ABSTRACT

Introduction: Delirium in critically ill adults is associated with prolonged hospital stay, increased mortality and greater cognitive and functional decline. Current practice guideline recommendations advocate the use of non-pharmacologic strategies to reduce delirium. The routine use of scheduled haloperidol to treat delirium is not recommended given a lack of evidence regarding its ability to resolve delirium nor improve relevant short and longer-term outcomes. This study aims to evaluate the efficacy and safety of haloperidol for the treatment of delirium in adult critically ill patients to reduce days spent with coma or delirium.

Methods and analysis: EuRIDICE is a prospective, multicentre, randomized, double-blind, placebo-controlled, trial. Study population consists of adult ICU patients without acute neurologic injury who have delirium based on a positive Intensive Care Delirium Screening Checklist (ICDSC) or Confusion Assessment Method for the ICU (CAM-ICU) assessment. Intervention is intravenous haloperidol 2.5 mg (or matching placebo) every 8 hours, titrated daily based on ICDSC or CAM-ICU positivity to a maximum of 5 mg every 8 hours, until delirium resolution or ICU discharge. Main study endpoint is delirium and coma free days (DCFD) up to 14 days after randomisation. Secondary endpoints include 1) 28-day and 1-year mortality; 2) cognitive and functional performance at 3 and 12 months; 3) patient- and family delirium and ICU experience; 4) psychological sequelae during and after ICU stay; 4) safety concerns associated with haloperidol use; and 5) cost-effectiveness. Differences in

For peer review only - http://bmjopen.bmj.com/site/about/guidelines.xhtml

DCFDs between haloperidol and placebo group will be analysed using Poisson regression analysis. Study recruitment started in February 2018 and continues.

Ethics and dissemination: The study has been approved by the Medical Ethics Committee of the Erasmus University Medical Centre Rotterdam (MEC2017-511). Its results will be disseminated via peer-reviewed publication and conference presentations.

Trial registration: ClinicalTrials, NCT03628391. Registered 14 August 2018 - https://clinicaltrials.gov/ct2/show/NCT03628391.

Strengths and limitations of this study

- This study is the first European sufficiently powered randomised multi-center double-blind placebo-controlled clinical trial;
- Extensive neurocognitive testing will be conducted with a valid test battery in order to assess cognitive impairment at 3 and 12 months after ICU admission;
- We will assess patient- and family experiences associated with delirium as a novel outcome;

INTRODUCTION

Delirium occurs in up to 80% of patients admitted to the Intensive Care Unit (ICU) (1, 2) and is associated with greater ICU and post-ICU mortality (2). Cognitive dysfunction and functional decline after critical illness is common, frequently persists for months after ICU discharge, and is worse among patients who experience delirium (2, 3). The symptoms and sequelae of delirium, including fear, anxiety, disrupted sleep, and post-traumatic stress disorder, may persist for months after ICU discharge. The health and societal costs of delirium are estimated to exceed $10 billion per year in the USA alone (4).

Given the burden and costs of delirium in critically ill adults, substantial research efforts have been devoted to identify safe and effective strategies to treat it. Current evidence and practice guideline recommendations advocate the use of non-pharmacologic strategies to reduce delirium, including avoidance of benzodiazepine sedation, early mobilization and

For peer review only - http://bmjopen.bmj.com/site/about/guidelines.xhtml

the use of sleep improvement protocols. The routine use of medication-based interventions to treat delirium, other than treatments to reduce the agitation that sometimes accompanies it, are not recommended (5, 6). The routine use of scheduled haloperidol to treat delirium is not currently recommended given a lack of current evidence regarding its ability to resolve delirium and its symptoms, nor improve relevant short and longer-term outcomes.

At the time this protocol was finalized, two randomized, placebo-controlled trials had evaluated haloperidol for ICU delirium prophylaxis or treatment and found haloperidol use did not affect days spent with delirium, days of mechanical ventilation, nor time spent in the ICU or hospital (7, 8). In one of these randomized controlled trials (RCTs), haloperidol use was associated with less agitation (7). Importantly, both studies were small (a combined total of 212 patients were enrolled), the ABCDEF bundle (a multimodal ICU bundle shown to reduce delirium by 50%)(9) was not routinely used, the effect of haloperidol on delirium-related symptoms was not evaluated, and the post-ICU, longer-term outcomes were not considered. Whether the response to haloperidol was different between patients with hyperactive versus hypoactive delirium was also not evaluated. The impact of haloperidol on patients'- and families' experiences with delirium after ICU discharge remains unknown. Whether long-term mortality is causally related to delirium or simply the persistent cognitive and functional decline associated with critical illness can only be established through a randomised trial (10). Moreover, the use of haloperidol in critically ill adults is not without potential safety concerns given it may prolong the QTc interval, induce extrapyramidal effects and cause oversedation. Despite haloperidol's lack of proven efficacy and the safety concerns associated with its use, haloperidol continues to be widely used in ICUs to treat of delirium (11).

In light of the above evidence gaps that were identified at the time this trial was conceptualized, there is a clear need for a large, multi-center, randomised controlled trial to better define the efficacy and safety of haloperidol to treat delirium in critically ill adults. This report describes the protocol for a large, multicentre, randomized, placebo-controlled,

For peer review only

For peer review only - http://bmjopen.bmj.com/site/about/guidelines.xhtml

haloperidol delirium trial that recently started enrolling patients across multiple ICUs in the Netherlands.

METHODS AND ANALYSIS

Study design

Randomized, double-blind, placebo-controlled trial of haloperidol for the treatment of delirium in patients admitted to one of six participating ICUs in the Rotterdam area in the Netherlands. See Appendix 1 for the participating hospitals.

Study population

Consecutive adults admitted to one of the participating ICUs.

Eligibility criteria

Inclusion criteria for eligibility:

1. Age ≥ 18 years
2. Admitted to the ICU.

Exclusion criteria for eligibility:

1. Admitted to the ICU with an acute neurological diagnosis (including acute stroke, traumatic brain injury, intracranial malignancy, anoxic coma). Prior non-acute stroke or another neurological condition without cognitive deterioration is not an exclusion criterion.
2. Pregnancy or lactation
3. History of ventricular arrhythmia including “torsade de pointes” (TdP)
4. Known allergy to haloperidol
5. History of dementia or an Informant Questionnaire on Cognitive Decline in the Elderly (IQCODE) score ≥ 4 (12)
6. History of malignant neuroleptic syndrome or parkinsonism (either Parkinson’s disease or another hypokinetic rigid syndrome)

For peer review only - http://bmjopen.bmj.com/site/about/guidelines.xhtml

For peer review only

7. Schizophrenia or other psychotic disorder
8. Inability to conduct valid delirium screening assessment (e.g. coma, deaf, blind) or inability to speak the Dutch language
9. Expected to die within 24 hours or leave the ICU within 24 hours

Inclusion criteria for randomisation:

1. Delirium, as assessed with the Intensive Care Delirium Screening Checklist (ICDSC ≥ 4) or the Confusion Assessment Method for the ICU (positive CAM-ICU assessment), at the time of ICU admission or any ICU day after ICU admission.
2. Written informed consent obtained from the patient or their legal representative
3. All eligibility *in*clusion criteria (from above) are still met.

Exclusion criteria for randomisation:

1. Prolonged QT-interval (QTc > 500ms)
2. (recent) “Torsade de pointes” (TdP)
3. (recent) Neuroleptic malignant syndrome or parkinsonism
4. Evidence of acute alcohol (or substance) withdrawal requiring pharmacological intervention (e.g. benzodiazepines or alpha-2 agonist) to treat
5. The patient is expected to die within 24 hours or expected to leave the ICU within 24 hours.
6. No (previously) signed informed consent by patient or representative
7. Current participation in another intervention trial that is evaluating a medication, device or behavioural intervention

Study outcomes

Main study outcome:

ICU delirium- and coma free days (DCFDs) (up to 14 days after randomisation).

Secondary study outcomes:

For peer review only

For peer review only - http://bmjopen.bmj.com/site/about/guidelines.xhtml

During ICU stay

- Richmond Agitation Sedation Scale (RASS)
- Maximum ICU Mobility Scale (IMS (13)) and day of max IMS.
- Quality of sleep (Richards-Campbell Sleep Questionnaire [RCSQ] (14) and with a visual analogue scale between 1-7 assessing the sleep quality according to the nurse).
- Use of “escape medication” for hallucinations and/or agitation (including atypical antipsychotics, alpha-2 agonists, GABA-agonists, opiates and “open-label” haloperidol).
- Daily study drug dose corrected for body weight (mg/kg).
- Self-extubation rate, removal of invasive devices (intravenous/-arterial catheters, drains and tubes).
- Adverse drug associated events (prolonged QTc by EKG, muscle rigidity and other associated movements disorders [Simpson Angus Scale (15)] and ventricular arrhythmia’s including torsade de pointes).
- Blood pressure will be recorded previous to and 1 hour after the first study drug dose (2.5mg equivalent) and 1 hour after the first 5mg equivalent.
- Daily respiratory status (regarding endotracheal intubation and mechanical ventilation)
- Time from randomisation to first resolution of delirium
- Time to “readiness for discharge from the ICU”

Hospital discharge

- Patient and family-member well-being and experiences associated with delirium during and after ICU stay with the ICU Memory Tool (ICU-MT (16)) and Delirium Experience Questionnaire (DEQ (17)).

28 days after randomization

- Mortality rate

For peer review only

3 months after randomization

- Cognitive outcomes with a detailed cognitive assessment battery of validated and repeatable measures of general cognition, memory, language, processing speed, attention and executive functioning and mood (Montreal Cognitive Assessment [MOCA](18), Rey Auditory Verbal Learning Test(19), Semantic fluency(20), Digit Span [WAIS-IV](21), Trail making tests A and B(22), Boston naming Test [short version](23), Hospital Anxiety and Depression Scale [HADS](24)).
- Functional outcomes and quality of life (Short Form-36 [SF-36](25)).
- Patient and family-member well-being and experiences associated with delirium during and after ICU stay with the ICU Memory Tool (ICU-MT (16)), Delirium Experience Questionnaire (DEQ (17)) and Caregiver Strain Index (CSI (26)).
- Posttraumatic stress syndrome (PTSS) in participants and family-members with the Impact of Event Scale – Revised (IES-R)(27).

12 months after randomization

- Cognitive outcomes with a detailed cognitive assessment battery of validated and repeatable measures of general cognition, memory, language, processing speed, attention and executive functioning and mood (Montreal Cognitive Assessment [MOCA](18), Rey Auditory Verbal Learning Test(19), Semantic fluency(20), Digit Span [WAIS-IV](21), Trail making tests A and B(22), Boston naming Test [short version](23), Hospital Anxiety and Depression Scale [HADS](24)).
- Functional outcomes and quality of life (Short Form-36 [SF-36](25)).
- Mortality rate

A cost-effectiveness analysis will be performed in collaboration with the Department of Health Policy and Management of Erasmus University Rotterdam (see Appendix 2 for more detailed explanation).The tools for the secondary outcomes are mentioned in Table 1 with overview of timing of assessments.

For peer review only - http://bmjopen.bmj.com/site/about/guidelines.xhtml

Treatment of subjects

Investigational product:

Name: Haldol (haloperidol)

Mechanism: butyrophenone-derived anti-psychotic with mainly dopamine-2 receptor antagonistic properties

Placebo consists of sodium chloride for injection. Medical staff, patients and family will be blinded to the product containing haloperidol/placebo.

Summary of findings from clinical studies and of known and potential risks and benefits:

See: Summary of Product Characteristics (SPC) in Appendix 3 and Systematic Review (Appendix 4).

Dosages, dosage modifications and method of administration:

The following dosing scheme will be used: start with haloperidol/placebo (further called: “study drug”) 2.5mg IV q8h (because of delirium screening once every 8-hour shift) and increase to a maximum dose of 5mg IV q8h when delirium persists during the next 8-hour shift. Doses will be reduced (50% of dose) in the very old elderly (age ≥ 80 years). The study drug dose will be decreased (when dosage is 5mg IV q8h) or stopped (when dose is 2.5mg IV q8h) when delirium has resolved (or is un-assessable due to coma) for the next 24 hours (implying: three consecutive delirium assessments during three shifts). Dosages can be lowered also at the discretion of the treating physician in case of evident rigidity, which is in line with current routine practice. Standard clinical practice for the administration of haloperidol will be followed.

Description and justification of route of administration and dosage:

Administration of the study intervention via the IV (versus the oral or enteral) route is the most feasible in critically ill patients – a population where gastrointestinal dysfunction is

For peer review only

For peer review only - http://bmjopen.bmj.com/site/about/guidelines.xhtml

prevalent and haloperidol absorption (i.e., bioavailability) could be compromised. The dose of haloperidol or placebo equivalent to be used in the study is based on the following consideration: 1. PK/PD; 2. Efficacy and 3. Safety. A (pilot) study in Erasmus Medical Center (n=14 critically ill patients, abstract presented at European Society of Intensive Care Medicine 2016) showed no adverse events (e.g. no QTc > 500ms), low serum levels (1.5-2.2μg/L) and no clear relation between serum level and delirium resolution with haloperidol dosages up to 2mg IV q8h (or: 3 x 2mg IV). A feasibility trial of haloperidol for ICU delirium (MIND-trial (8)) that used an average total daily dosage of 15 mg orally found higher serum levels (interquartile range 2.85-5.8 μg/L). No differences were found in QTc prolongation between treatment groups and placebo in this trial. None of these trials demonstrated clinically important safety concerns associated with haloperidol administration. Finally, a recently published trial of haloperidol for ICU delirium using haloperidol/placebo 10mg IV q12h, did not report any safety issues, using a QTc cut-off for safety of 550ms, which may be regarded an indirect signal that such dosages are feasible and safe (28).The maximum dose of haloperidol of up to 5mg IV q8h was further chosen because a previous Dutch guideline advocating the use of haloperidol recommended an IV haloperidol treatment dose of up to 20 mg/24h period (29). In our protocol, we chose q8h dosing (titrated up to 15mg daily) given the greater potential susceptibility of critically ill adults to the side effects of haloperidol, and the fact that this dosage is in line with existing haloperidol delirium protocols in several of the participating ICUs.

<u>Patient assessments:</u>

Rigidity will be monitored with the Simpson-Angus scale (15) and the Barnes Akathisia Rating Scale (30) (see "Secondary study endpoints") for study purposes only. The QTc interval will be measured daily before the administration of the second daily (afternoon) dose using a 12-lead EKG. When the QTc interval is found to be prolonged (> 500ms or an increase from baseline (=at randomisation) of ≥ 60ms (31, 32)), all non-study medications having the potential to prolong the QTc will be held if clinically feasible. A Standard Operating

For peer review only - http://bmjopen.bmj.com/site/about/guidelines.xhtml

Procedure (S.O.P.) lists the drugs known to prolong the QTc. Eight hours later, if QTc prolongation persists, study medication will be held or tapered according to the S.O.P. and only resumed when the EKGs (evaluation frequency increased to q8h in this situation) reveal QTc prolongation to have dissipated.

<u>General medical management at participating ICUs:</u>

In the six original participating ICUs, institutional delirium guidelines, based on the 2013 PAD guidelines and a Dutch ICU delirium guideline, were rigorously implemented over a three-year period (2012 to 2015) (6, 33, 34). During the inclusion period of the current trial, spot-checks will be performed by members of the investigative team at each center to confirm delirium screening accuracy, as a quality-of-assessments measure and these will be documented in a qualitative manner.

<u>Preparation and labelling of Investigational Medicinal Product:</u>

Preparation and labelling will be done by the trial pharmacist ("Apotheek A15") according to GMP guidelines. Apotheek A15 is certified for these procedures. Trial medication will be dispensed to the pharmacies of the trial sites by the Hospital Pharmacy of Erasmus MC. See Appendix 5 for a description of the drug accountability.

<u>Escape medication:</u>

Knowing that half the subjects will be administered placebo, we anticipate two issues may affect the clinical management of enrolled patients: 1) agitation and 2) hallucinations.

Agitation management will be based on the following principles: a) treat pain first with opioids; b) use alpha-2 agonist for agitation that either persists or is not caused by pain; c) GABA agonists (e.g. benzodiazepines or propofol) are discouraged, but can be used on a short-term basis for the treatment of severe agitation (RASS ≥ 2) that cannot be effectively managed by other means.

For peer review only - http://bmjopen.bmj.com/site/about/guidelines.xhtml

Hallucination management will be based on the following principles: a) pharmacological treatment may be withheld if the patient indicates they are not in distress; b) for a patient in distress, a low-dose atypical antipsychotic (e.g., quetiapine 12.5mg q8h) may be administered on a short-term basis until the distress resolves.

Because of the pragmatic design of this trial, within these boundaries, the treatment and dose of escape medication is left to the treating physician, since these are part of routine practice. However, before start of randomisation these management principles for agitation and hallucination will be thoroughly implemented first with the help of detailed S.O.P.'s to enhance uniformity in participating centres. Adherence to escape medication regimens will be closely monitored. Open-label haloperidol administration is strongly discouraged during the trial but can be used if the ICU team considers it necessary for acute breakthrough delirium symptoms that cannot be managed within the management boundaries outlined above. Open-label haloperidol will be documented.

Randomisation, blinding and treatment allocation

Legal representatives of eligible patients (when the patient is sedated or otherwise temporarily unable to consent) or the patient him-/herself will be asked for informed consent shortly after admission when the patient has no delirium, or as soon as possible after admission when the patient already has delirium. In this study the presence of delirium will be considered to be confirmed when the beside nurse deemed the patient to have delirium based on assessment with the ICDSC or CAM-ICU, given the previous large-scale implementation project (33).

Delirious patients who fulfil all inclusion but no exclusion criteria, and for whom written informed consent has been obtained (as recorded in medical file), will be randomised. Randomisation coordination and start of a new Case Record Form (CRF) will be guided by the Electronic Data Capture (EDC) system of ALEA, constructed by the Clinical Trial Center (CTC) of the Erasmus Medical Center and calibrated with the coordinating (Erasmus MC) and local pharmacies. We will randomise the recruited patients using a block design of 8

For peer review only - http://bmjopen.bmj.com/site/about/guidelines.xhtml

patients in one block, and one block is assigned to a center. We will have 8 batches (numbered 1 through 8) of treatment and placebo, with 4 batches of placebo and 4 treatment (haloperidol). Each block will have a random assignment of 8 batch numbers, having four placebo and four haloperidol patients included (a combination of 1 to 4 and 5 to 8 in random order). After 8 patients are included in the study (i.e., a block is full), a new block will be assigned to a center.

Upon randomisation, the study drug with the corresponding randomisation kit number 1-8 (based on 8 medication batches consisting of either haloperidol or placebo) will be obtained from the hospital pharmacy of each participating ICU. Each box from a batch/kit contains 10 ampules (5mg/1ml) of haloperidol or placebo. If all ampules are used, a new box from the same medication kit number with 10 ampules will be used. Study drugs are administered on prescription in the electronic patient data management system (PDMS) and are double-checked by ICU nurses before administration, which is similar to regular practice. Furthermore, the kit number was noted upon randomisation in the medical file and the kit number could be retrieved at any time from the PDMS after first prescription upon randomisation.

Blinding of the medication will be performed by the pharmacy, based on a randomisation list that will be generated electronically through a randomisation module in the EDC system of ALEA. Randomisation will be stratified per study center (i.e. equal number of patients in both study groups, see “statistical analysis” paragraph). Only the involved pharmacists and the trial statistician are aware of the contents of each medication kit. Only the local (site) pharmacists are able to unblind study treatment of a patient in case of an emergency. Except for the hospital’s pharmacist responsible for the randomisation list, all other involved personnel with the study, caregivers, patients or their representatives will remain unaware of the treatment groups until the time of Database Lock. The Unblinding procedure is specified in Appendix 6.

For peer review only - http://bmjopen.bmj.com/site/about/guidelines.xhtml

Follow-up procedures will be performed according to designated S.O.P.'s. When possible and preferred by patients or families, questionnaires will be sent or visits planned at home when possible, e.g. for incapacitated participants.

Withdrawal of individual subjects

Subjects can leave the study at any time for any reason if they wish to do so without any consequences. The investigator can decide to withdraw a subject from the study for urgent medical reasons.

Follow-up of subjects withdrawn from treatment

Data of withdrawn patients will remain in the database for statistical analysis purposes but will not be subject to follow-up. When patients specifically withdraw their consent for usage of their data, these data will be removed from the database and excluded from all analyses.

Premature termination of the study

The sponsor may decide to terminate the study prematurely based on the following criteria:

- There is evidence of an unacceptable risk for study patients (i.e. safety issue)
- There is reason to conclude that continuation of the study cannot serve a scientific purpose following confirmation of the Data Safety Monitoring Board (DSMB)
- The DSMB recommends to end the trial based on viable arguments other than described above.

The following stopping rules have been determined by the DSMB and have been laid down in a DSMB charter:

- Early stopping of one individual participant, for example, to clear benefit or harm of a treatment or the occurrence of serious adverse reactions or events in one patient. In this case de-blinding of this single patient may be necessary.

For peer review only

For peer review only - http://bmjopen.bmj.com/site/about/guidelines.xhtml

- Stopping of the trial as a whole to clear benefit or harm of a treatment or the occurrence of serious adverse reactions or events. As a result, further patient enrolment will be stopped. Deblinding may be necessary for all patients.

Reasons to stop the study include:

- Advice to do so from DSMB
- Interim analysis shows a significant benefit difference between the treatment groups which will not be expected to change after inclusion of all subject as per the power analysis.

SUSAR's are not expected due to the vast experience in clinical practice with the study drug (haloperidol).

If the study is terminated the Medical Ethics Committees of all participating hospital and the CCMO will be notified.

Safety reporting

<u>AEs, SAEs and SUSARs:</u>

Adverse events (AEs)

Adverse events are defined as any undesirable experience occurring to a subject during the study, whether or not considered related to the investigational product. Since patients admitted to an ICU are critically ill and present with many AEs, only possible adverse drug related events (on days of study drug administration: prolonged QTc by EKG, muscle rigidity and associated movements disorders [Simpson Angus Scale]) as indicated by the subject or observed by the investigator or his staff occurring from the date of randomisation until 14 days later or discharge from ICU or death (whichever comes first), will be recorded in the CRF. In addition, the following AEs will be assessed daily during 14 days after randomisation: epilepsy, tachycardia, hypotension (not explained otherwise), hepatic

For peer review only - http://bmjopen.bmj.com/site/about/guidelines.xhtml

For peer review only

dysfunction (not explained otherwise), leucopenia (not explained otherwise), bronchospasms (not explained otherwise).

Serious adverse events (SAEs)

A SAE is any untoward medical occurrence or effect, occurring during the 14-day study period at the ICU, that (the SAEs for the purpose of the study are shown in *Italics* per item)

- results in death;
    - *death will always be reported as an SAE*
- is life threatening (at the time of the event);
    - *ventricular arrhythmia or malignant neuroleptic syndrome*
- requires hospitalisation or prolongation of existing inpatients' hospitalisation;
    - *Not to be expected; only applicable when the site investigator is able to explicitly show a relationship*
- results in persistent or significant disability or incapacity;
    - *Not to be expected; only applicable when the site investigator is able to explicitly show a relationship*
- is a congenital anomaly or birth defect; (*Not applicable*) or
- any other important medical event that did not result in any of the outcomes listed above due to medical or surgical intervention but could have been based upon appropriate judgement by the investigator.

For peer review only

Statistical analysis

<u>Primary and secondary study parameter(s):</u>

Statistical analysis will be done according to intention-to-treat-principle. All randomised participants will be included. The primary outcome is DCFDs, defined as the number of days in the first 14 days after randomisation during which the patient is alive without delirium and not in coma from any cause (7). Patients who are discharged before the 14 day study period has ended, will be recorded as delirium and coma-free after discharge (8, 35). Additionally,

we will assume all patients who died within 14 days after randomisation to have 0 delirium and coma free days (7). Differences between DCFDs between the haloperidol group and placebo group will be analyzed using Poisson regression analysis, with adjustment for differences in baseline characteristics between treatment groups (when present) and for the different centers. We will collect data with regards to baseline demographics: age, sex, admission diagnosis category, APACHE II and APACHE IV, SOFA, ICU days before study entry and pre-admission delirium duration in participants with delirium on admission. Pre-defined sub-analyses will include efficacy stratified by 1) agitated, mixed-type or hypoactive delirium; 2) the presence of hallucinations or delusions; 3) delirium severity (based on ICDSC score); and 4) sedation-related, hypoxic, metabolic or septic delirium. For cognitive and functional outcomes assessed with designated test-batteries, non-parametric or parametric tests will be used depending on normality of scaled test-results. Mortality risk will be assessed as a binary end-point. A more detailed statistical analysis plan, to be drawn up before Data Base Lock, will be drafted for publication separately.

Interim analysis:

Pre-planned interim analyses will be performed at 1/3 and 2/3 of the trial's course (first analysis ideally estimated at 6 months after start of trial), as determined by the DSMB charter or otherwise when the DSMB requests it.

Sample size calculation

To achieve statistically significant results with ($p<.05$) with a power of 90% and a true treatment difference of one day for the primary outcome (from 3.2 DCFDs in the placebo group to 4.2 in the haloperidol group, SD in both groups is equal to 4.2), 371 patients are needed in each group (n=742). These estimates are derived from the previous implementation study, which included 4727 patients in three 4-month periods in the same six participating ICUs and found delirium incidence of 27% (and increase of DCFDs from 60% to 70%)(33). Consequently, presuming an informed consent rate of 40%, we need 18-months to

For peer review only - http://bmjopen.bmj.com/site/about/guidelines.xhtml

For peer review only

encounter 1900 patients with a newly diagnosed delirium to include the required 742 patients. Because of estimated work-load due to follow-up visits, including e.g. neurocognitive testing, we propose to select a convenience sample of 2/3 of ICU survivors (estimated around 400 of 575 survivors) as a random sample for the cognitive, functional and secondary outcome variables.

Patient and Public Involvement

During the design and conduct of the study we involved two ex-ICU patients as patient-perspective representatives. The primary research question, its outcome measures, and the burden of the intervention have been assessed and found relevant by these patient-representatives. The role and tasks of the patient-representatives for the study have been detailed as: 1) to help select meaningful assessment-tools of patient and family experiences during and after ICU stay, 2) act as liaison between the study management team and the Dutch foundation “Family and patient Centered Intensive Care” (FCIC; one representative is a formal representative for FCIC), 3) act as members of the Stakeholders group to provide advice on the study contents, execution and course at on a regular basis to ensure the patient and family perspective, 4) advise on the contents of the Patient Information Form (PIF) and the informed consent procedure, 5) advise on ways to minimise loss to follow-up for the functional and cognitive outcome assessments, 6) advise on contents and organisation of symposia during the study on delirium and its consequences with the aim to better inform participants of the study and their family members and maximize their involvement, 7) advise on the contents of the supporting website of the trial. Study participants will be informed about the most important results of the trial, either by post or symposium, when they indicate this on the informed consent letter.

ETHICS AND DISSEMINATION

The study has been approved by the Medical Ethics Committee of the Erasmus University Medical Centre Rotterdam (MEC2017-511) and the Institutional Review Boards of

For peer review only - http://bmjopen.bmj.com/site/about/guidelines.xhtml

participating sites. The study will be conducted according to the principles of the Declaration of Helsinki (version, date, see for the most recent version: www.wma.net) and in accordance with the Medical Research Involving Human Subjects Act (WMO) and other guidelines, regulations and Acts.

Recruitment and consent

Recruitment of eligible patients will be done upon admission. Informed consent for *possible* participation (i.e. only when participants develop delirium at the ICU) will be obtained from subjects who are not expected to leave the ICU within the first 24 hours after admission and are not yet delirious. The informed consent will be obtained from the patient or (if the patient is unable to consent) from patient's representative. This procedure of prior request for informed consent will facilitate randomisation when the patient indeed develops delirium, because randomisation can then be performed 24/7 since informed consent is already obtained and delirium often surfaces during the evening and night when obtaining informed consent is difficult. The informed consent procedure will be clearly delineated from the randomisation procedure. Importantly, when a patient with prior informed consent develops delirium and can thus be randomised, still a pre-randomisation check with regard to in- and exclusion criteria will be performed to confirm that the patient fulfils the inclusion, and not the exclusion criteria (because this may change over time). A team of dedicated research and ICU nurses and physicians (local PI, PhD student, PI, post-doc) will be trained to perform the informed consent procedures and help with the randomisations. Moreover, a 24/7 study consultation telephone number will be opened to help with problems or question during the study. A second type of randomisation concerns patients who are delirious upon admission to ICU. These patients' next-of-kin will be asked to grant permission to participate by means of informed consent when they are legally representative for the patients and the patient has no contraindications. After informed consent is obtained, the patient can be randomised.

REFERENCES

For peer review only

For peer review only - http://bmjopen.bmj.com/site/about/guidelines.xhtml

1. Salluh JI, Soares M, Teles JM, Ceraso D, Raimondi N, Nava VS, et al. Delirium epidemiology in critical care (DECCA): an international study. Crit Care. 2010;14(6):R210.

2. Ely EW, Shintani A, Truman B, Speroff T, Gordon SM, Harrell FE, Jr., et al. Delirium as a predictor of mortality in mechanically ventilated patients in the intensive care unit. JAMA. 2004;291(14):1753-62.

3. Pandharipande PP, Girard TD, Ely EW. Long-term cognitive impairment after critical illness. N Engl J Med. 2014;370(2):185-6.

4. Milbrandt EB, Deppen S, Harrison PL, Shintani AK, Speroff T, Stiles RA, et al. Costs associated with delirium in mechanically ventilated patients. Crit Care Med. 2004;32(4):955-62.

5. Reade MC, Eastwood GM, Bellomo R, Bailey M, Bersten A, Cheung B, et al. Effect of Dexmedetomidine Added to Standard Care on Ventilator-Free Time in Patients With Agitated Delirium: A Randomized Clinical Trial. JAMA. 2016;315(14):1460-8.

6. Devlin JW, Skrobik Y, Gelinas C, Needham DM, Slooter AJC, Pandharipande PP, et al. Clinical Practice Guidelines for the Prevention and Management of Pain, Agitation/Sedation, Delirium, Immobility, and Sleep Disruption in Adult Patients in the ICU. Crit Care Med. 2018;46(9):e825-e73.

7. Page VJ, Ely EW, Gates S, Zhao XB, Alce T, Shintani A, et al. Effect of intravenous haloperidol on the duration of delirium and coma in critically ill patients (Hope-ICU): a randomised, double-blind, placebo-controlled trial. Lancet Respir Med. 2013;1(7):515-23.

8. Girard TD, Pandharipande PP, Carson SS, Schmidt GA, Wright PE, Canonico AE, et al. Feasibility, efficacy, and safety of antipsychotics for intensive care unit delirium: the MIND randomized, placebo-controlled trial. Crit Care Med. 2010;38(2):428-37.

9. Barnes-Daly MA, Phillips G, Ely EW. Improving Hospital Survival and Reducing Brain Dysfunction at Seven California Community Hospitals: Implementing PAD Guidelines Via the ABCDEF Bundle in 6,064 Patients. Crit Care Med. 2017;45(2):171-8.

10. Al-Qadheeb NS, Balk EM, Fraser GL, Skrobik Y, Riker RR, Kress JP, et al. Randomized ICU trials do not demonstrate an association between interventions that reduce delirium duration and short-term mortality: a systematic review and meta-analysis. Crit Care Med. 2014;42(6):1442-54.

11. Collet MO, Caballero J, Sonneville R, Bozza FA, Nydahl P, Schandl A, et al. Prevalence and risk factors related to haloperidol use for delirium in adult intensive care patients: the multinational AID-ICU inception cohort study. Intensive Care Med. 2018;44(7):1081-9.

12. Jorm AF, Jacomb PA. The Informant Questionnaire on Cognitive Decline in the Elderly (IQCODE): socio-demographic correlates, reliability, validity and some norms. Psychol Med. 1989;19(4):1015-22.

13. Hodgson C, Needham D, Haines K, Bailey M, Ward A, Harrold M, et al. Feasibility and inter-rater reliability of the ICU Mobility Scale. Heart Lung. 2014;43(1):19-24.

14. Richards KC, O'Sullivan PS, Phillips RL. Measurement of sleep in critically ill patients. J Nurs Meas. 2000;8(2):131-44.

15. Simpson GM, Angus JW. A rating scale for extrapyramidal side effects. Acta Psychiatr Scand Suppl. 1970;212:11-9.

16. Jones C, Griffiths RD, Humphris G, Skirrow PM. Memory, delusions, and the development of acute posttraumatic stress disorder-related symptoms after intensive care. Crit Care Med. 2001;29(3):573-80.

17. Breitbart W, Gibson C, Tremblay A. The delirium experience: delirium recall and delirium-related distress in hospitalized patients with cancer, their spouses/caregivers, and their nurses. Psychosomatics. 2002;43(3):183-94.

18. Nasreddine ZS, Phillips NA, Bedirian V, Charbonneau S, Whitehead V, Collin I, et al. The Montreal Cognitive Assessment, MoCA: a brief screening tool for mild cognitive impairment. J Am Geriatr Soc. 2005;53(4):695-9.

19. Bean J. Rey Auditory Verbal Learning Test, Rey AVLT. In: Kreutzer JS, DeLuca J, Caplan B, editors. Encyclopedia of Clinical Neuropsychology. New York, NY: Springer New York; 2011. p. 2174-5.

For peer review only

For peer review only - http://bmjopen.bmj.com/site/about/guidelines.xhtml

20. Ledoux K, Vannorsdall TD, Pickett EJ, Bosley LV, Gordon B, Schretlen DJ. Capturing additional information about the organization of entries in the lexicon from verbal fluency productions. J Clin Exp Neuropsychol. 2014;36(2):205-20.
21. Wechsler D. Wechsler adult intelligence scale–Fourth Edition (WAIS–IV). San Antonio, TX: NCS Pearson. 2008;22:498.
22. Reitan RM, Wolfson D. The Halstead–Reitan neuropsychological test battery for adults—Theoretical, methodological, and validational bases. Neuropsychological assessment of neuropsychiatric and neuromedical disorders. 2009;1:36.
23. Roth C. Boston Naming Test. In: Kreutzer JS, DeLuca J, Caplan B, editors. Encyclopedia of Clinical Neuropsychology. New York, NY: Springer New York; 2011. p. 430-3.
24. Zigmond AS, Snaith RP. The hospital anxiety and depression scale. Acta Psychiatr Scand. 1983;67(6):361-70.
25. Ware JE, Jr., Sherbourne CD. The MOS 36-item short-form health survey (SF-36). I. Conceptual framework and item selection. Med Care. 1992;30(6):473-83.
26. Robinson BC. Validation of a Caregiver Strain Index. J Gerontol. 1983;38(3):344-8.
27. Weiss DS, Marmar CR, Wilson JP, Keane TM. Assessing psychological trauma and PTSD. The Impact of Events Scale—Revised. 1997;19:399-411.
28. Girard TD, Exline MC, Carson SS, Hough CL, Rock P, Gong MN, et al. Haloperidol and Ziprasidone for Treatment of Delirium in Critical Illness. The New England journal of medicine. 2018;379(26):2506-16.
29. Nederlandse Vereniging voor Psychiatrie. Richtlijn delirium. Amsterdam: Dries van Ingen, Uitgeverij Boom; 2004. p. 1-127.
30. Barnes TR. The Barnes Akathisia Rating Scale--revisited. J Psychopharmacol. 2003;17(4):365-70.
31. Fongemie JM, Al-Qadheeb NS, Estes NA, 3rd, Roberts RJ, Temtanakitpaisan Y, Ruthazer R, et al. Agreement between ICU clinicians and electrophysiology cardiologists on the decision to initiate a QTc-interval prolonging medication in critically ill patients with potential risk factors for torsade de pointes: a comparative, case-based evaluation. Pharmacotherapy. 2013;33(6):589-97.
32. Tisdale JE, Jaynes HA, Kingery JR, Overholser BR, Mourad NA, Trujillo TN, et al. Effectiveness of a clinical decision support system for reducing the risk of QT interval prolongation in hospitalized patients. Circ Cardiovasc Qual Outcomes. 2014;7(3):381-90.
33. Trogrlic Z, van der Jagt M, Lingsma H, Gommers D, Ponssen HH, Schoonderbeek JFJ, et al. Improved Guideline Adherence and Reduced Brain Dysfunction After a Multicenter Multifaceted Implementation of ICU Delirium Guidelines in 3,930 Patients. Crit Care Med. 2019.
34. Barr J, Fraser GL, Puntillo K, Ely EW, Gelinas C, Dasta JF, et al. Clinical practice guidelines for the management of pain, agitation, and delirium in adult patients in the intensive care unit. Crit Care Med. 2013;41(1):263-306.
35. van den Boogaard M, Slooter AJC, Bruggemann RJM, Schoonhoven L, Beishuizen A, Vermeijden JW, et al. Effect of Haloperidol on Survival Among Critically Ill Adults With a High Risk of Delirium: The REDUCE Randomized Clinical Trial. JAMA. 2018;319(7):680-90.

AUTHORS' CONTRIBUTIONS

MJ developed the study protocol, edited and approved the final version, and was responsible for funding and supervising the study coordination. ZT contributed to the protocol development and study implementation. LS is responsible for study coordination. WR assisted with statistical analysis and NH assisted with pharmacological coordination. RO, AS,

For peer review only - http://bmjopen.bmj.com/site/about/guidelines.xhtml

HP and JD were involved in the study design and protocol development. All authors contributed to the development and refinement of this study protocol. They have read and approved the final version of the protocol.

Acknowledgements

EuRIDICE study group authors:

Local principal investigators: M. van den Boogaard (15), A.J.B.W. Brouwers (7), J.A. Lens (9), B.J.M. van der Meer (8) , H. Ponssen (4), F.J. Schoonderbeek (10), K.S. Simons (16).

Local research nurses: E. Berger (7), A. Bouman (16), M. Campo (8), D. van Duijn (1), H. Embden – van Donk (10), D. van de Graaf (9), E. Hoogendoorn (4), P. Ormskerk (1), N. Roovers (15), E. Toscano (8), A. Vileito (1), T. van Zuylen (16).

All involved local principal investigators (MB, AB, BM, JL, EK, FS, KS) and research nurses (EB, AB, DD, HE, DG, PO, NR, AV, MC, ET, EH, TZ) have facilitated visits at their site, will be involved in executing the protocol at their sites and will be responsible for patient recruitment and data collection in their hospitals, along with follow up of study patients.

Other members involved: C. Exler (11), E. van den Berg (12), J. van Meeteren (13), M. Koopmanschap (14).

Patient representatives: I. Nutma and E. Kuijper.

1) Erasmus MC-University Medical Center, Department of Intensive Care Adults, Rotterdam, The Netherlands, Netherlands

4) Albert Schweitzer Hospital, Department of Intensive Care, Dordrecht, Netherlands

7) Franciscus Gasthuis, Department of Intensive Care, Rotterdam, Netherlands

8) Maasstad Hospital, Department of Intensive Care, Rotterdam, Netherlands

9) IJsselland Hospital, Department of Intensive Care, Capelle aan den IJssel, Netherlands

10) Ikazia Hospital, Department of Intensive Care, Rotterdam, Netherlands

For peer review only

11) Erasmus MC-University Medical Center, Department of Pharmacy, Rotterdam, The Netherlands, Netherlands

12) Erasmus MC-University Medical Center, Department of Neuropsychology, Rotterdam, The Netherlands, Netherlands

13) Erasmus MC-University Medical Center, Department of Rehabilitation Medicine, Rotterdam, The Netherlands, Netherlands

14) Erasmus School of Health Policy & Management, Department of Health Economics and HTA, Rotterdam, The Netherlands, Netherlands

15) Radboud University Medical Center, Department of Intensive Care Medicine, Nijmegen, The Netherlands

16) Jeroen Bosch Hospital, Department of Intensive Care Medicine, 's-Hertogenbosch, The Netherlands.

For peer review only

FUNDING STATEMENT

A grant has been provided by ZonMw – The Netherlands Organisation for Health Research and Organisation. ZonMw project number: 848041001. This funding source had no role in the design of this study and will not have any role during its execution, analyses, interpretation of the data, or decision to submit results.

COMPETING INTERESTS STATEMENT

The authors declare that they have no competing interests.

LIST OF ABBREVIATIONS

**ABR**	**ABR form, General Assessment and Registration form, is the application form that is required for submission to the accredited Ethics Committee (In Dutch, ABR = Algemene Beoordeling en Registratie)**
**AE**	**Adverse Event**
**AR**	**Adverse Reaction**
**CA**	**Competent Authority**

For peer review only - http://bmjopen.bmj.com/site/about/guidelines.xhtml

**CAM-ICU**	**Confusion Assessment Method for the ICU**
**CCMO**	**Central Committee on Research Involving Human Subjects; in Dutch: Centrale Commissie Mensgebonden Onderzoek**
**CV**	**Curriculum Vitae**
**DSMB**	**Data Safety Monitoring Board**
**EU**	**European Union**
**EudraCT**	**European drug regulatory affairs Clinical Trials**
**EKG**	**Electrocardiography**
**GCP**	**Good Clinical Practice**
**IB**	**Investigator's Brochure**
**IC**	**Informed Consent**
**ICDSC**	**Intensive Care Delirium Screening Checklist**
**ICU**	**Intensive Care Unit**
**IMP**	**Investigational Medicinal Product**
**IMPD**	**Investigational Medicinal Product Dossier**
**METC**	**Medical research ethics committee (MREC); in Dutch: medisch ethische toetsing commissie (METC)**
**PAD**	**Pain, agitation and delirium**
**RCT**	**Randomized Controlled Trial**
**(S)AE**	**(Serious) Adverse Event**
**S.O.P.**	**Standard Operating Procedure**
**SPC**	**Summary of Product Characteristics (in Dutch: officiële productinfomatie IB1-tekst)**
**Sponsor**	**The sponsor is the party that commissions the organisation or performance of the research, for example a pharmaceutical company, academic hospital, scientific organisation or investigator. A party that provides funding for a study but does not commission it is not regarded as the sponsor, but referred to as a subsidising party.**
**SUSAR**	**Suspected Unexpected Serious Adverse Reaction**
**TdP**	**Torsade de Pointes**
**Wbp**	**Personal Data Protection Act (in Dutch: Wet Bescherming Persoonsgevens)**
**WMO**	**Medical Research Involving Human Subjects Act (in Dutch: Wet Medisch-wetenschappelijk Onderzoek met Mensen)**

For peer review only

Table 1. Overview of timing of assessments, including required time investment per visit/questionnaire.

Moment (months)	Neurocognitive tests	Patient and family experiences (time in min.)	Functional outcomes (SF-36)	Cost effectivity questionnaires (EQ-5D-5L, iMTA MCQ, iMTA PCQ)	Other
**Enrolment**					Informed consent, IQCODE-N, pregnancy test (if applicable), EKG.
**ICU study period (3x / day)**					CAM-ICU / ICDSC, RASS
**ICU study period (once daily)**					IMS, RCSQ. Only when on study medication: EKG, Simpson Angus Scale.
**0 (discharge from hospital)**		Patient: ICU-MT (15) + DEQ (15) Family: DEQ (2)			
**1**				30 min.	

For peer review only

For peer review only - http://bmjopen.bmj.com/site/about/guidelines.xhtml

**3**	45-60 min.	Patient: IES-R (5) + ICU-MT (15) + DEQ (15)<br>Family: IES-R (5) + CSI (5) + DEQ (2)	10 min.	30 min.	
**6**				30 min.	
**12**	45-60 min.		10 min.	30 min.	

IQCODE-N = Informant Questionnaire on Cognitive Decline in the Elderly – Dutch version

EKG = Electrocardiography

Neurocognitive tests: Montreal Cognitive Assessment (MOCA), Rey Auditory Verbal Learning Test, Semantic fluency, Digit Span (WAIS-IV), Trailmaking tests A and B, Boston naming Test (short version), Hospital Anxiety and Depression Scale (HADS)

IMS = ICU Mobility Scale, measures mobility during ICU admission

RCSQ = Richards-Campbell Sleep Questionnaire, measures quality of sleep

Simpson Angus Scale = measures muscle rigidity and other associated movements disorders

ICU-MT= ICU-Memory Tool, assesses the experience and memories of ICU admission

DEQ= Delirium Experience Questionnaire, measures experiences linked to delirium

IES-R = Impact of Event Scale Revised, assesses distress linked to a traumatic experience (i.e. experiencing delirium)

CSI = Caregiver Strain Index, assesses the strain experienced by the caregiver

SF-36 = Short Form-36, measures the health-related quality of life

EQ-5D-5L = assesses the general health status

iMTA MCQ = instituut Beleid & Management Gezondheidszorg Medical Consumption Questionnaire (health care use)

iMTA PCQ = instituut Beleid & Management Gezondheidszorg Productivity Cost Questionnaire (productivity costs)

With the exception of the neurocognitive tests, all above mentioned tools are questionnaires that can be administered at home. Real life visits only need to be paid in order to perform the neurocognitive tests.

For peer review only - http://bmjopen.bmj.com/site/about/guidelines.xhtml

For peer review only

Appendix 1: Participating hospitals

Erasmus MC Rotterdam

Albert Schweizer Hospital Dordrecht

Maasstad Hospital Rotterdam

IJsselland Hospital Capelle aan den IJssel

Ikazia Hospital Rotterdam

Franciscus Gasthuis Rotterdam

As of July 2019 two additional ICUs have started recruitment:

Jeroen Bosch Hospital, 's-Hertogenbosch

Radboud University Medical Center, Nijmegen

For peer review only

Appendix 2: Economic evaluation

ECONOMIC EVALUATION

General considerations

The primary economic analysis will be a trial-based **cost-utility analysis** from a societal and a healthcare perspective. This analysis will be performed according to the Dutch guidelines (1, 2). The time horizon will be 12 months after randomisation, in order to take all relevant costs and effects regarding the treatment procedure into account. Additionally, a **cost-effectiveness analysis** performed from a societal and health care perspective will be conducted, using delirium-free and coma-free days as outcomes.

If a difference in quality of life is observed at the end of the follow-up period, we will also perform a **model-based extrapolation** of costs and health benefits up to 5 years, exploring the following scenarios: (1) health benefit remain constant after the follow-up period, (2) health benefits are gradually phased out over the course of the modelling time, (3) health benefits are gradually phased out over the course of the modelling time over the first year after follow-up, (4) health benefits abruptly disappear after the follow-up period, but costs remain until the end of the modelling period.

If treatment with haloperidol leads to better health outcomes at higher costs, or if it leads to worse health outcomes and cost savings, incremental cost-utility and incremental cost-effectiveness ratios will be calculated. These express the additional costs per unit of health gain (QALYs, delirium-free days, coma-free days) or the savings per unit of health forgone. The uncertainty around the estimates will be addressed using bootstrapping for the analysis of costs and effects in the first 12 months, and using probabilistic sensitivity analysis in the extrapolation model.

Cost analysis

Healthcare costs will be calculated based on patient-level data on health-care utilization, which will be collected from hospital databases and questionnaires, to be filled out at regular intervals by patients and/or informal caregivers. Cost categories include medication, screenings, inpatient days, contacts with healthcare providers (GP, outpatient visits, and therapists). The questionnaire will also contain questions about absence from paid work by the patient and informal caregivers.

Costs will be calculated by multiplying resource utilization with the cost per unit of resource. Some unit costs will be taken from the 2016 Dutch Manual for Costing Studies(3), but the costs of inpatient days will be assessed following the micro-costing method, which is based on comprehensive ‘bottom-up’ analyses of the activities of staff and other resources that are used

during those days. Medication prices will be based on the official list prices, including value added tax and increased by a standard prescription reimbursement for the pharmacist. The cost of production loss will be calculated according to the Friction Cost Approach.

Patient outcome analysis

The primary outcome measure in the economic evaluation is the difference in QALYs. The secondary effects are the delirium-free and coma-free days after treatment with haloperidol or placebo. As measuring QALYs in adult critically ill patients is not feasible at baseline, it is not possible to estimate the average number of QALYs for each treatment group. However, assuming that there is no difference at randomisation, it is possible to analyse the difference in quality of life at subsequent measurements in a multilevel regression model. This will enable us to calculate a difference in QALYs between the treatment groups over the total follow-up period, using linear intrapolation. HRQoL will be measured on t=1, 3, 6 and 12 months after randomization using the EQ-5D-5L instrument.

References:

1. Hakkaart-van Roijen L., Tan SS., Bouwmans CAM. Handleiding voor kostenonderzoek, methoden en standaard kostprijzen voor economische evaluaties in de gezondheidszorg. College voor zorgverzekeringen, Geactualiseerde versie 2010.
2. Zorginstituut Nederland. Richtlijn voor het uitvoeren van economische evaluaties in de gezondheidszorg. 2015.
3. Hakkaart-van Roijen L., van der Linden N., Bouwmans CAM., Kanters T., Tan SS. Costing manual: Methodology of costing research and reference prices for economic evaluations in healthcare. 2015.

For peer review only

Appendix 3: Haloperidol SPC

See this weblink: https://db.cbg-meb.nl/IB-teksten/h03185.pdf

For peer review only

Appendix 4: (Semi-) structured review of literature on efficacy and adverse events of haloperidol for delirium in adult critically ill patients

1. Haloperidol as a treatment for ICU delirium

Systematic review of randomised placebo-controlled trials assessing haloperidol for treatment of ICU delirium

Method: A biomedical Information Specialist (BIS) of the Erasmus Medical Center library performed a systematic search aimed at controlled studies on haloperidol for ICU delirium combining the subjects: delirium, ICU and haloperidol, or equivalent terms (see: Appendix for details). No distinction was made in the search between treatment or prevention trials.

Review: Since focus of the EuRIDICE study is on a haloperidol versus placebo comparison, the study selection for this summary is also focused on placebo-controlled haloperidol trials for the treatment of ICU delirium. Systematic reviews from the systematic search are used as a crosscheck to confirm completeness or provide additional insights. The search (total of yielded only 1 study. The MIND trial (2010) was a randomised placebo controlled feasibility, efficacy and safety trial of antipsychotics for ICU delirium in adult mechanically ventilated medical and surgical patients (1). It included three treatment arms (haloperidol, n=35; ziprasidone, n=30 and placebo, n=36) and used a well thought out design (excluding demented patients with a validated tool for cognitive dysfunction, using CAM-ICU as a validated screening tool, a clear protocol with regard to QTc prolongation and study drug dosing, measuring extrapyramidal symptoms with a validated scale and with number of days alive without delirium and coma as the primary outcome (indicating total burden of brain dysfunction, since only assessing delirium days may result in increased coma days and less delirium days being regarded as a – false – improvement). The study used oral haloperidol, no clear sedation protocol aimed at light sedation and crossover antipsychotics were allowed but discouraged. No clear differences were found in the three groups with regard to the primary outcome. Mean haloperidol dose was 15 mg a day but QTc prolongation and extrapyramidal symptoms did not differ between treatment groups. Other medications in this small trial did not differ between groups (propofol, opiates, benzodiazepines). It was concluded that a larger trial would be safe and feasible.

Overview of most recent guidelines' statements on haloperidol as treatment for ICU delirium

Method: Pubmed search on published guidelines including ICU delirium and containing information on

For peer review only - http://bmjopen.bmj.com/site/about/guidelines.xhtml

haloperidol. Search terms: guideline, delirium, ICU.

Review: Three recent guidelines were retrieved (2-4). In a Danish guideline (2015) no evidence is stated for pharmacological management(2). A German guideline (2015) advocates symptom-based therapy when delirium screening is positive with haloperidol as a first choice in case of delirium associated with psychotic symptoms only. The “Clinical practice guidelines for the management of pain, agitation, and delirium in adult patients in the intensive care unit” (2013)(3) advocate avoiding ‘antipsychotics’ when risk of torsades de pointes or is present or either baseline QT prolongation or concomitant QT prolonging medication is used. It states that there is no evidence that haloperidol decrease delirium duration, which was perceived as the most relevant issue to address with regard to haloperidol treatment of ICU delirium.

Cochrane review(s)

Method: Search on Cochrane (http://www.cochranelibrary.com) for reviews with search term: ‘delirium’, does not elicit any results pertaining to pharmacological treatment of delirium nor haloperidol.

Review: no Cochrane reviews exist on (ICU) delirium and it’s pharmacological management.

On-going trials

Method: A search for ‘haloperidol’ and ‘delirium’ in the following online trial databases (and including ICU patients); www.trialregister.nl (0 trials); www.clinicaltrials.gov (4 trials).

Review: Four trials were retrieved from www.clinicaltrials.gov. One trial (‘Haloquet’) was not a truly placebo controlled trial because haloperidol was allowed (‘as needed’) in the placebo group and was last updated in 2013 but not published. It consisted of three treatments arms (also quetiapine) and aimed to include a total of 45 patients (and should thus be considered a pilot trial and not an efficacy trial). A second trial enrolled 40 patients and was completed in 2011 but not published. A third trial was a phase-2 safety/efficacy study enrolling 20 patients, last updated in 2007 and not published. The fourth trial (‘The modifying the impact of ICU-associated neurological dysfunction-USA [MIND-USA] study’) is currently recruiting (last verified May 2016 on September 14th). It is a multi-center double blind placebo-controlled trial aiming to enrol 561 patients in three treatment arms: haloperidol, ziprasidone and placebo, by the same research group that did the MIND trial. It includes cognitive and

For peer review only

psychological follow-up at 12 months and is estimated to be completed July 2019. Maximum dose of haloperidol amounts to 10 mg IV q12 hours. Trial design is similar to the EuRIDICE trial, except for the patient experiences and perspective, and the fact that only patients on mechanical ventilation or in shock are included (i.e. the sickest ICU patients). The study protocol has not been published in a peerreviewed journal.

2. Haloperidol to prevent ICU delirium

Systematic review of randomised placebo-controlled trials assessing haloperidol for prevention of ICU delirium; including information from guidelines and Cochrane reviews

Method: A biomedical Information Specialist (BIS) of the Erasmus Medical Center library performed a systematic search aimed at controlled studies on haloperidol for ICU delirium combining the subjects: delirium, ICU and haloperidol, or equivalent terms (see: Appendix for details).

Review: the focus of this section is on randomised placebo-controlled prevention trials of haloperidol for ICU delirium. Three trials were retrieved. One trial included post-operative generally non-critically ill patients (5) and was not further considered for this review. The Hope-ICU trial (2013)(6) was a prophylactic study of haloperidol (2.5mg IV q8h, n=71) versus placebo (n=70) in adult mechanically ventilated ICU patients. The primary end-point of delirium (assessed with CAM-ICU) and coma free days did not differ between groups (5 days in both), but there was a 21% crossover rate with haloperidol in the placebo group. Secondary clinical endpoints such as length of stay at ICU or mortality did not differ but the trial was not powered on these outcomes. Another trial (2016)(7) including mechanically ventilated patients (n=68) with ‘subsyndromal’ delirium (=an Intensive Care Delirium Screening Checklist [ICDSC] score of 1-3 on a scale of 8, where 4 or more is compatible with delirium) used haloperidol 1mg IV q6h but did not find lower rate of progression to full delirium.

3. Haloperidol: adverse events versus treatment effects in the few available trials

The adverse events associated with haloperidol in the three aforementioned (small) trials (one treatment and two prevention trials) did not include QTc prolongation (with a threshold of >500 ms). In the Hope-ICU trial more opiates and sedatives were administered in the placebo-group but alfa-2

For peer review only

agonists were not clearly protocolled, more agitation was present and 26% versus 11% antipsychotics' use in the placebo group. The subsyndromal delirium trial similarly found more agitation in the placebo group.

4. Healthcare perspective

A cost-effectiveness analysis of the Hope-ICU trial found that delirium increased cognitive dysfunction at 6 months and reduced quality of life, suggestive of potential cost-effectiveness of haloperidol (8).

Acknowledgements:

Gerdien B. de Jonge (MSc), biomedical information specialist, Medical Library, Erasmus MC, is kindly acknowledged for her help in assembling the databases for the systematic review.

References

1. Girard TD, Pandharipande PP, Carson SS, Schmidt GA, Wright PE, Canonico AE, et al. Feasibility, efficacy, and safety of antipsychotics for intensive care unit delirium: The mind randomized, placebo-controlled trial. Crit Care Med. 2010;38(2):428-37.
2. Fonsmark L, Hein L, Nibroe H, Bundgaard H, de Haas I, Iversen S, et al. Danish national sedation strategy. Targeted therapy of discomfort associated with critical illness. Danish society of intensive care medicine (dsit) and the danish society of anesthesiology and intensive care medicine (dasaim). Dan Med J. 2015;62(4):C5052.
3. Barr J, Fraser GL, Puntillo K, Ely EW, Gelinas C, Dasta JF, et al. Clinical practice guidelines for the management of pain, agitation, and delirium in adult patients in the intensive care unit. Crit Care Med. 2013;41(1):263-306.
4. D. A. S. Taskforce, Baron R, Binder A, Biniek R, Braune S, Buerkle H, et al. Evidence and consensus based guideline for the management of delirium, analgesia, and sedation in intensive care medicine. Revision 2015 (das-guideline 2015) - short version. Ger Med Sci. 2015;13:Doc19.
5. Wang W, Li HL, Wang DX, Zhu X, Li SL, Yao GQ, et al. Haloperidol prophylaxis decreases delirium incidence in elderly patients after noncardiac surgery: A randomized controlled trial*. Crit Care Med. 2012;40(3):731-9.
6. Page VJ, Ely EW, Gates S, Zhao XB, Alce T, Shintani A, et al. Effect of intravenous haloperidol on the duration of delirium and coma in critically ill patients (hope-icu): A randomised, double-blind, placebo-controlled trial. Lancet Respir Med. 2013;1(7):515-23.
7. Al-Qadheeb NS, Skrobik Y, Schumaker G, Pacheco MN, Roberts RJ, Ruthazer RR, et al. Preventing icu subsyndromal delirium conversion to delirium with low-dose iv haloperidol: A double-blind, placebo-controlled pilot study. Crit Care Med. 2016;44(3):583-91.
8. Joachim M, Daniel FM, Annalisa C, Timothy A, Xiao BZ, Eugene WE, et al. Health evaluation and cost-effectiveness analysis from a randomized trial of haloperidol in the management of delirium in the critically ill (hope-icu trial). C24 sedation, delirium, and neurocognitive dysfunction in critical care. p. A4022-A.

For peer review only

Appendix 5: Drug Accountability

Upon randomisation, the study drug with the corresponding randomisation kit number 1-8 (based on 8 medication batches consisting of either haloperidol or placebo) will be obtained from the hospital pharmacy of each participating ICU. Each box from a batch/kit contains 10 ampules (5mg/1ml) of haloperidol or placebo. If all ampules are used, a new box from the same medication kit number with 10 ampules will be used. Study drugs are administered on prescription in the electronic patient data management system (PDMS) and are double-checked by ICU nurses before administration, which is similar to regular practice. Furthermore, the kit number was noted upon randomisation in the medical file and the kit number could be retrieved at any time from the PDMS after first prescription upon randomisation.

The research nurse of each participating ICU will record the number of the box with study drug for each patient in the CRF.

The research nurse of each participating ICU is responsible for retrieving the boxes with study drug. The amount of vials in the boxes will be counted for each patient and will be noted in the CRF. The research nurse will return unused drug to the hospital pharmacy. The hospital pharmacy will destroy the vials with study drug and will also record this (double administration).

The pharmacist or another appropriate individual who is designated should maintain records of the product's delivery to the trial site, the inventory at the site, the use by each patient, problems and irregularities during injection, the maintenance of the blinding, and the return to the pharmacy of unused product(s). These records should include dates, quantities, batch/serial numbers, expiration dates (if applicable), and the unique code numbers assigned to the investigational product(s) and trial patients (if applicable). Investigators should maintain records that document adequately that the patients were provided the doses specified by the protocol and reconcile all investigational product(s).

For peer review only

Appendix 6: Unblinding Procedure

The study treatment will be unblinded after Database Lock. While the safety of patients should always take priority, maintenance of blinding is crucial to the integrity of a double-blind trial. Before this planned unblinding, the blinding for a specific patient should only be broken when information about the patient's protocol treatment is considered necessary to manage Serious Adverse Events (emergency unblinding). Unblinding procedures should preferably be initiated only after consultation of the principal investigator/coordinating investigator or his/her representative. To initiate an emergency unblinding the pharmacy in charge of the randomisation list should be contacted. Breaking the blinding on a patient will be logged and reported to the coordinating Investigator within 24 hours following the unblinding procedure, using the Emergency Unblinding Form. It is considered a major protocol violation, after which the patient goes off protocol treatment (if applicable).

For peer review only - http://bmjopen.bmj.com/site/about/guidelines.xhtml

Reporting checklist for protocol of a clinical trial.

Based on the SPIRIT guidelines.

Instructions to authors

Complete this checklist by entering the page numbers from your manuscript where readers will find each of the items listed below.

Your article may not currently address all the items on the checklist. Please modify your text to include the missing information. If you are certain that an item does not apply, please write "n/a" and provide a short explanation.

Upload your completed checklist as an extra file when you submit to a journal.

In your methods section, say that you used the SPIRITreporting guidelines, and cite them as:

Chan A-W, Tetzlaff JM, Altman DG, Laupacis A, Gøtzsche PC, Krleža-Jerić K, Hróbjartsson A, Mann H, Dickersin K, Berlin J, Doré C, Parulekar W, Summerskill W, Groves T, Schulz K, Sox H, Rockhold FW, Rennie D, Moher D. SPIRIT 2013 Statement: Defining standard protocol items for clinical trials. Ann Intern Med. 2013;158(3):200-207

		Reporting Item	Page Number
**Administrative information**			
Title	#1	Descriptive title identifying the study design, population, interventions, and, if applicable, trial acronym	1
Trial registration	#2a	Trial identifier and registry name. If not yet registered, name of intended registry	3
Trial registration: data set	#2b	All items from the World Health Organization Trial Registration Data Set	NA
Protocol version	#3	Date and version identifier	2
Funding	#4	Sources and types of financial, material, and other support	22

For peer review only

Roles and responsibilities: contributorship	#5a	Names, affiliations, and roles of protocol contributors	1, 20
Roles and responsibilities: sponsor contact information	#5b	Name and contact information for the trial sponsor	1,2
Roles and responsibilities: sponsor and funder	#5c	Role of study sponsor and funders, if any, in study design; collection, management, analysis, and interpretation of data; writing of the report; and the decision to submit the report for publication, including whether they will have ultimate authority over any of these activities	22
Roles and responsibilities: committees	#5d	Composition, roles, and responsibilities of the coordinating centre, steering committee, endpoint adjudication committee, data management team, and other individuals or groups overseeing the trial, if applicable (see Item 21a for data monitoring committee)	14
**Introduction**			
Background and rationale	#6a	Description of research question and justification for undertaking the trial, including summary of relevant studies (published and unpublished) examining benefits and harms for each intervention	3-5
Background and rationale: choice of comparators	#6b	Explanation for choice of comparators	3-5
Objectives	#7	Specific objectives or hypotheses	4
Trial design	#8	Description of trial design including type of trial (eg, parallel group, crossover, factorial, single group), allocation ratio, and framework (eg, superiority, equivalence, non-inferiority, exploratory)	4
**Methods: Participants,**			

For peer review only

**interventions, and outcomes**			
Study setting	#9	Description of study settings (eg, community clinic, academic hospital) and list of countries where data will be collected. Reference to where list of study sites can be obtained	5
Eligibility criteria	#10	Inclusion and exclusion criteria for participants. If applicable, eligibility criteria for study centres and individuals who will perform the interventions (eg, surgeons, psychotherapists)	5-6
Interventions: description	#11a	Interventions for each group with sufficient detail to allow replication, including how and when they will be administered	9-10
Interventions: modifications	#11b	Criteria for discontinuing or modifying allocated interventions for a given trial participant (eg, drug dose change in response to harms, participant request, or improving / worsening disease)	9-12
Interventions: adherance	#11c	Strategies to improve adherence to intervention protocols, and any procedures for monitoring adherence (eg, drug tablet return; laboratory tests)	11
Interventions: concomitant care	#11d	Relevant concomitant care and interventions that are permitted or prohibited during the trial	11-12
Outcomes	#12	Primary, secondary, and other outcomes, including the specific measurement variable (eg, systolic blood pressure), analysis metric (eg, change from baseline, final value, time to event), method of aggregation (eg, median, proportion), and time point for each outcome. Explanation of the clinical relevance of chosen efficacy and harm outcomes is strongly recommended	7-9
Participant timeline	#13	Time schedule of enrolment, interventions (including any run-ins and washouts), assessments, and visits for participants. A schematic diagram is highly recommended (see Figure)	7-9
Sample size	#14	Estimated number of participants needed to achieve study objectives and how it was determined, including	17

For peer review only

		clinical and statistical assumptions supporting any sample size calculations	
Recruitment	#15	Strategies for achieving adequate participant enrolment to reach target sample size	12
**Methods: Assignment of interventions (for controlled trials)**			
Allocation: sequence generation	#16a	Method of generating the allocation sequence (eg, computer-generated random numbers), and list of any factors for stratification. To reduce predictability of a random sequence, details of any planned restriction (eg, blocking) should be provided in a separate document that is unavailable to those who enrol participants or assign interventions	12-13
Allocation concealment mechanism	#16b	Mechanism of implementing the allocation sequence (eg, central telephone; sequentially numbered, opaque, sealed envelopes), describing any steps to conceal the sequence until interventions are assigned	12-13
Allocation: implementation	#16c	Who will generate the allocation sequence, who will enrol participants, and who will assign participants to interventions	12-13
Blinding (masking)	#17a	Who will be blinded after assignment to interventions (eg, trial participants, care providers, outcome assessors, data analysts), and how	12-13
Blinding (masking): emergency unblinding	#17b	If blinded, circumstances under which unblinding is permissible, and procedure for revealing a participant's allocated intervention during the trial	12-13
**Methods: Data collection, management, and analysis**			
Data collection plan	#18a	Plans for assessment and collection of outcome, baseline, and other trial data, including any related processes to promote data quality (eg, duplicate	7-9

For peer review only

		measurements, training of assessors) and a description of study instruments (eg, questionnaires, laboratory tests) along with their reliability and validity, if known. Reference to where data collection forms can be found, if not in the protocol	
Data collection plan: retention	#18b	Plans to promote participant retention and complete follow-up, including list of any outcome data to be collected for participants who discontinue or deviate from intervention protocols	13-14
Data management	#19	Plans for data entry, coding, security, and storage, including any related processes to promote data quality (eg, double data entry; range checks for data values). Reference to where details of data management procedures can be found, if not in the protocol	12, 15
Statistics: outcomes	#20a	Statistical methods for analysing primary and secondary outcomes. Reference to where other details of the statistical analysis plan can be found, if not in the protocol	16
Statistics: additional analyses	#20b	Methods for any additional analyses (eg, subgroup and adjusted analyses)	16-17
Statistics: analysis population and missing data	#20c	Definition of analysis population relating to protocol non-adherence (eg, as randomised analysis), and any statistical methods to handle missing data (eg, multiple imputation)	14
**Methods: Monitoring**			
Data monitoring: formal committee	#21a	Composition of data monitoring committee (DMC); summary of its role and reporting structure; statement of whether it is independent from the sponsor and competing interests; and reference to where further details about its charter can be found, if not in the protocol. Alternatively, an explanation of why a DMC is not needed	14
Data monitoring: interim analysis	#21b	Description of any interim analyses and stopping guidelines, including who will have access to these	16-17

For peer review only

		interim results and make the final decision to terminate the trial	
Harms	#22	Plans for collecting, assessing, reporting, and managing solicited and spontaneously reported adverse events and other unintended effects of trial interventions or trial conduct	15-16
Auditing	#23	Frequency and procedures for auditing trial conduct, if any, and whether the process will be independent from investigators and the sponsor	NA
**Ethics and dissemination**			
Research ethics approval	#24	Plans for seeking research ethics committee / institutional review board (REC / IRB) approval	17
Protocol amendments	#25	Plans for communicating important protocol modifications (eg, changes to eligibility criteria, outcomes, analyses) to relevant parties (eg, investigators, REC / IRBs, trial participants, trial registries, journals, regulators)	17
Consent or assent	#26a	Who will obtain informed consent or assent from potential trial participants or authorised surrogates, and how (see Item 32)	18
Consent or assent: ancillary studies	#26b	Additional consent provisions for collection and use of participant data and biological specimens in ancillary studies, if applicable	NA
Confidentiality	#27	How personal information about potential and enrolled participants will be collected, shared, and maintained in order to protect confidentiality before, during, and after the trial	18
Declaration of interests	#28	Financial and other competing interests for principal investigators for the overall trial and each study site	22
Data access	#29	Statement of who will have access to the final trial dataset, and disclosure of contractual agreements that limit such access for investigators	-

For peer review only

Ancillary and post trial care	#30	Provisions, if any, for ancillary and post-trial care, and for compensation to those who suffer harm from trial participation	NA
Dissemination policy: trial results	#31a	Plans for investigators and sponsor to communicate trial results to participants, healthcare professionals, the public, and other relevant groups (eg, via publication, reporting in results databases, or other data sharing arrangements), including any publication restrictions	3
Dissemination policy: authorship	#31b	Authorship eligibility guidelines and any intended use of professional writers	-
Dissemination policy: reproducible research	#31c	Plans, if any, for granting public access to the full protocol, participant-level dataset, and statistical code	NA
**Appendices**			
Informed consent materials	#32	Model consent form and other related documentation given to participants and authorised surrogates	-
Biological specimens	#33	Plans for collection, laboratory evaluation, and storage of biological specimens for genetic or molecular analysis in the current trial and for future use in ancillary studies, if applicable	NA

None The SPIRIT checklist is distributed under the terms of the Creative Commons Attribution License CC-BY-ND 3.0. This checklist can be completed online using https://www.goodreports.org/, a tool made by the EQUATOR Network in collaboration with Penelope.ai

For peer review only

BMJ Open

Efficacy of halopeRIdol to decrease the burden of Delirium In adult Critically ill patiEnts (EuRIDICE): study protocol for a prospective randomised multi-center double-blind placebo-controlled clinical trial in the Netherlands

Journal:	*BMJ Open*
Manuscript ID	bmjopen-2019-036735.R2
Article Type:	Protocol
Date Submitted by the Author:	16-Jul-2020

Complete List of Authors:	Smit, Lisa; Erasmus Medical Center, Department of Intensive Care Adults<br>Trogrlić, Zoran; Erasmus Medical Center, Department of Intensive Care Adults<br>Devlin, John; Northeastern University Bouve College of Health Sciences, Department of Pharmacy and Health Systems Sciences; Tufts Medical Center, Division of Pulmonary, Critical Care and Sleep Medicine<br>Osse, Robert-Jan; Erasmus Medical Center, Department of Psychiatry<br>Ponssen, Huibert; Albert Schweitzer Hospital Location Dordwijk, Department of Intensive Care<br>Slooter, Arjen ; University Medical Centre Utrecht Brain Centre, Department of Intensive Care Medicine and UMC Utrecht Brain Center<br>Hunfeld, Nicole; Erasmus Medical Center, Department of Pharmacy and Department of Intensive Care<br>Rietdijk, Wim; Erasmus Medical Center, Department of Intensive Care Adults<br>Gommers, Diederik; Erasmus Medical Center, Department of Intensive Care Adults<br>van der Jagt, Mathieu; Erasmus Medical Center, Department of Intensive Care Adults

**Primary Subject Heading**:	Intensive care
Secondary Subject Heading:	Intensive care, Neurology, Mental health, Pharmacology and therapeutics
Keywords:	Delirium & cognitive disorders < PSYCHIATRY, INTENSIVE & CRITICAL CARE, Adult intensive & critical care < ANAESTHETICS, MENTAL HEALTH, Adult neurology < NEUROLOGY, THERAPEUTICS

SCHOLARONE™
Manuscripts

I, the Submitting Author has the right to grant and does grant on behalf of all authors of the Work (as defined in the below author licence), an exclusive licence and/or a non-exclusive licence for contributions from authors who are: i) UK Crown employees; ii) where BMJ has agreed a CC-BY licence shall apply, and/or iii) in accordance with the terms applicable for US Federal Government officers or employees acting as part of their official duties; on a worldwide, perpetual, irrevocable, royalty-free basis to BMJ Publishing Group Ltd ("BMJ") its licensees and where the relevant Journal is co-owned by BMJ to the co-owners of the Journal, to publish the Work in this journal and any other BMJ products and to exploit all rights, as set out in our licence.

The Submitting Author accepts and understands that any supply made under these terms is made by BMJ to the Submitting Author unless you are acting as an employee on behalf of your employer or a postgraduate student of an affiliated institution which is paying any applicable article publishing charge ("APC") for Open Access articles. Where the Submitting Author wishes to make the Work available on an Open Access basis (and intends to pay the relevant APC), the terms of reuse of such Open Access shall be governed by a Creative Commons licence – details of these licences and which Creative Commons licence will apply to this Work are set out in our licence referred to above.

Other than as permitted in any relevant BMJ Author's Self Archiving Policies, I confirm this Work has not been accepted for publication elsewhere, is not being considered for publication elsewhere and does not duplicate material already published. I confirm all authors consent to publication of this Work and authorise the granting of this licence.

For peer review only

Efficacy of halopeRIdol to decrease the burden of Delirium In adult Critically ill patiEnts (EuRIDICE): study protocol for a prospective randomised multi-center double-blind placebo-controlled clinical trial in the Netherlands

Lisa Smit – l.smit@erasmusmc.nl (1), Zoran Trogrlic – z.trogrlic@erasmusmc.nl (1), John W. Devlin - j.devlin@northeastern.edu (2), Robert Jan Osse - r.osse@erasmusmc.nl (3), Huibert H. Ponssen - H.H.Ponssen@asz.nl (4), Arjen J.C. Slooter - A.Slooter-3@umcutrecht.nl (5), Nicole G.M. Hunfeld - n.hunfeld@erasmusmc.nl (1, 6), Wim J.R. Rietdijk - w.rietdijk@erasmusmc.nl (1), Diederik Gommers - d.gommers@erasmusmc.nl (1), Mathieu van der Jagt – m.vanderjagt@erasmusmc.nl* (1), on behalf of the EuRIDICE study group.

1) Erasmus MC-University Medical Center, Department of Intensive Care Adults, Rotterdam, The Netherlands, Netherlands

2) Bouve College of Health Sciences, Northeastern University; Division of Pulmonary, Critical Care and Sleep Medicine, Department of Pharmacy and Health Systems Sciences, Boston, United States

3) Erasmus MC-University Medical Center, Department of Psychiatry, Rotterdam, The Netherlands, Netherlands

4) Albert Schweitzer Hospital, Department of Intensive Care, Dordrecht, Netherlands

5) University Medical Center Utrecht, Utrecht University, Department of Intensive Care Medicine and UMC Utrecht Brain Center, Utrecht, The Netherlands

6) Erasmus MC-University Medical Center, Departments of Intensive Care Adults and Pharmacy, Rotterdam, The Netherlands, Netherlands

*Corresponding author:

Department of Intensive Care, room Ne-415

Erasmus MC

For peer review only

For peer review only - http://bmjopen.bmj.com/site/about/guidelines.xhtml

PO BOX 2040
3000 CA Rotterdam
E-mail: m.vanderjagt@erasmusmc.nl
Telephone number: +31107040704

Protocol version: November 16th 2017, Version 2

Word count: 4629 words

For peer review only

ABSTRACT

Introduction: Delirium in critically ill adults is associated with prolonged hospital stay, increased mortality and greater cognitive and functional decline. Current practice guideline recommendations advocate the use of non-pharmacologic strategies to reduce delirium. The routine use of scheduled haloperidol to treat delirium is not recommended given a lack of evidence regarding its ability to resolve delirium nor improve relevant short and longer-term outcomes. This study aims to evaluate the efficacy and safety of haloperidol for the treatment of delirium in adult critically ill patients to reduce days spent with coma or delirium.

Methods and analysis: EuRIDICE is a prospective, multicentre, randomized, double-blind, placebo-controlled, trial. Study population consists of adult ICU patients without acute neurologic injury who have delirium based on a positive Intensive Care Delirium Screening Checklist (ICDSC) or Confusion Assessment Method for the ICU (CAM-ICU) assessment. Intervention is intravenous haloperidol 2.5 mg (or matching placebo) every 8 hours, titrated daily based on ICDSC or CAM-ICU positivity to a maximum of 5 mg every 8 hours, until delirium resolution or ICU discharge. Main study endpoint is delirium and coma free days (DCFD) up to 14 days after randomisation. Secondary endpoints include 1) 28-day and 1-year mortality; 2) cognitive and functional performance at 3 and 12 months; 3) patient- and family delirium and ICU experience; 4) psychological sequelae during and after ICU stay; 4) safety concerns associated with haloperidol use; and 5) cost-effectiveness. Differences in

For peer review only - http://bmjopen.bmj.com/site/about/guidelines.xhtml

DCFDs between haloperidol and placebo group will be analysed using Poisson regression analysis. Study recruitment started in February 2018 and continues.

Ethics and dissemination: The study has been approved by the Medical Ethics Committee of the Erasmus University Medical Centre Rotterdam (MEC2017-511) and by the Institutional Review Boards of the participating sites. Its results will be disseminated via peer-reviewed publication and conference presentations.

Trial registration: ClinicalTrials, NCT03628391. Registered 14 August 2018 - https://clinicaltrials.gov/ct2/show/NCT03628391. Netherlands Trial Registry, NTR6725. Registered 29 September 2017 https://www.trialregister.nl/trial/6537.

Strengths and limitations of this study

- This study is the first sufficiently powered randomised multi-center double-blind placebo-controlled clinical trial in Europe;
- Extensive neurocognitive testing will be conducted with a valid test battery in order to assess cognitive impairment at 3 and 12 months after ICU admission;
- We will assess patient- and family experiences associated with delirium as a novel outcome;
- There are little data on the optimal haloperidol regimen in ICU patients; the maximum haloperidol dose of 15mg/day in our study may still be subtherapeutic.
- Lack of true clinical equipoise among nurses and physicians regarding the use of haloperidol may hamper motivation for the study.

INTRODUCTION

Delirium occurs in up to 80% of patients admitted to the Intensive Care Unit (ICU) (1, 2) and is associated with greater ICU and post-ICU mortality (2). Cognitive dysfunction and functional decline after critical illness is common, frequently persists for months after ICU discharge, and is worse among patients who experience delirium (2, 3). The symptoms and sequelae of delirium, including fear, anxiety, disrupted sleep, and post-traumatic stress

For peer review only - http://bmjopen.bmj.com/site/about/guidelines.xhtml

disorder, may persist for months after ICU discharge. The health and societal costs of delirium are estimated to exceed $10 billion per year in the USA alone (4).

Given the burden and costs of delirium in critically ill adults, substantial research efforts have been devoted to identify safe and effective strategies to treat it. Current evidence and practice guideline recommendations advocate the use of non-pharmacologic strategies to reduce delirium, including avoidance of benzodiazepine sedation, early mobilization and the use of sleep improvement protocols. The routine use of medication-based interventions to treat delirium, other than treatments to reduce the agitation that sometimes accompanies it, are not recommended (5, 6). The routine use of scheduled haloperidol to treat delirium is not currently recommended given a lack of current evidence regarding its ability to resolve delirium and its symptoms, nor improve relevant short and longer-term outcomes.

At the time this protocol was finalized, two randomized, placebo-controlled trials had evaluated haloperidol for ICU delirium prophylaxis or treatment and found haloperidol use did not affect days spent with delirium, days of mechanical ventilation, nor time spent in the ICU or hospital (7, 8). In one of these randomized controlled trials (RCTs), haloperidol use was associated with less agitation (7). Importantly, both studies were small (a combined total of 212 patients were enrolled), the ABCDEF bundle (a multimodal ICU bundle shown to reduce delirium by 50%)(9) was not routinely used, the effect of haloperidol on delirium-related symptoms was not evaluated, and the post-ICU, longer-term outcomes were not considered. Whether the response to haloperidol was different between patients with hyperactive versus hypoactive delirium was also not evaluated. The impact of haloperidol on patients'- and families' experiences with delirium after ICU discharge remains unknown. Whether long-term mortality is causally related to delirium or simply the persistent cognitive and functional decline associated with critical illness can only be established through a randomised trial (10). Moreover, the use of haloperidol in critically ill adults is not without potential safety concerns given it may prolong the QTc interval, induce extrapyramidal effects and cause oversedation. Despite haloperidol's lack of proven efficacy and the safety concerns

For peer review only - http://bmjopen.bmj.com/site/about/guidelines.xhtml

associated with its use, haloperidol continues to be widely used in ICUs to treat of delirium (11).

In light of the above evidence gaps that were identified at the time this trial was conceptualized, there is a clear need for a large, multi-center, randomised controlled trial to better define the efficacy and safety of haloperidol to treat delirium in critically ill adults. This report describes the protocol for a large, multicentre, randomized, placebo-controlled, haloperidol delirium trial that recently started enrolling patients across multiple ICUs in the Netherlands.

METHODS AND ANALYSIS

Study design

Randomized, double-blind, placebo-controlled trial of haloperidol for the treatment of delirium in patients admitted to one of six participating ICUs in the Rotterdam area in the Netherlands. See Appendix 1 for the participating hospitals.

Study population

Consecutive adults admitted to one of the participating ICUs.

Eligibility criteria

Inclusion criteria for eligibility:

1. Age ≥ 18 years
2. Admitted to the ICU.

Exclusion criteria for eligibility:

1. Admitted to the ICU with an acute neurological diagnosis (including acute stroke, traumatic brain injury, intracranial malignancy, anoxic coma). Prior non-acute stroke or another neurological condition without cognitive deterioration is not an exclusion criterion.
2. Pregnancy or lactation

For peer review only - http://bmjopen.bmj.com/site/about/guidelines.xhtml

3. History of ventricular arrhythmia including "torsade de pointes" (TdP)
4. Known allergy to haloperidol
5. History of dementia or an Informant Questionnaire on Cognitive Decline in the Elderly (IQCODE) score $\geq$ 4 (12)
6. History of malignant neuroleptic syndrome or parkinsonism (either Parkinson's disease or another hypokinetic rigid syndrome)
7. Schizophrenia or other psychotic disorder
8. Inability to conduct valid delirium screening assessment (e.g. coma, deaf, blind) or inability to speak the Dutch language
9. Expected to die within 24 hours or leave the ICU within 24 hours

<u>Inclusion criteria for randomisation:</u>

1. Delirium, as assessed with the Intensive Care Delirium Screening Checklist (ICDSC $\geq$ 4) or the Confusion Assessment Method for the ICU (positive CAM-ICU assessment), at the time of ICU admission or any ICU day after ICU admission.
2. Written informed consent obtained from the patient or their legal representative
3. All eligibility *in*clusion criteria (from above) are still met.

<u>Exclusion criteria for randomisation:</u>

1. Prolonged QT-interval (QTc > 500ms)
2. (recent) "Torsade de pointes" (TdP)
3. (recent) Neuroleptic malignant syndrome or parkinsonism
4. Evidence of acute alcohol (or substance) withdrawal requiring pharmacological intervention (e.g. benzodiazepines or alpha-2 agonist) to treat
5. The patient is expected to die within 24 hours or expected to leave the ICU within 24 hours.
6. No (previously) signed informed consent by patient or representative
7. Current participation in another intervention trial that is evaluating a medication, device or behavioural intervention

For peer review only

Study outcomes

<u>Main study outcome:</u>

ICU delirium- and coma free days (DCFDs) (up to 14 days after randomisation).

<u>Secondary study outcomes:</u>

During ICU stay

- Richmond Agitation Sedation Scale (RASS)
- Maximum ICU Mobility Scale (IMS (13)) and day of max IMS.
- Quality of sleep (Richards-Campbell Sleep Questionnaire [RCSQ] (14) and with a visual analogue scale between 1-7 assessing the sleep quality according to the nurse).
- Use of “escape medication” for hallucinations and/or agitation (including atypical antipsychotics, alpha-2 agonists, GABA-agonists, opiates and “open-label” haloperidol).
- Daily study drug dose corrected for body weight (mg/kg).
- Self-extubation rate, removal of invasive devices (intravenous/-arterial catheters, drains and tubes).
- Adverse drug associated events (prolonged QTc by EKG, muscle rigidity and other associated movements disorders [Simpson Angus Scale (15)] and ventricular arrhythmia’s including torsade de pointes).
- Blood pressure will be recorded previous to and 1 hour after the first study drug dose (2.5mg equivalent) and 1 hour after the first 5mg equivalent.
- Daily respiratory status (regarding endotracheal intubation and mechanical ventilation)
- Time from randomisation to first resolution of delirium
- Time to “readiness for discharge from the ICU”

For peer review only - http://bmjopen.bmj.com/site/about/guidelines.xhtml

Hospital discharge

- Patient and family-member well-being and experiences associated with delirium during and after ICU stay with the ICU Memory Tool (ICU-MT (16)) and Delirium Experience Questionnaire (DEQ (17)).

28 days after randomization

- Mortality rate

3 months after randomization

- Cognitive outcomes with a detailed cognitive assessment battery of validated and repeatable measures of general cognition, memory, language, processing speed, attention and executive functioning and mood (Montreal Cognitive Assessment [MOCA](18), Rey Auditory Verbal Learning Test(19), Semantic fluency(20), Digit Span [WAIS-IV](21), Trail making tests A and B(22), Boston naming Test [short version](23), Hospital Anxiety and Depression Scale [HADS](24)).
- Functional outcomes and quality of life (Short Form-36 [SF-36](25)).
- Patient and family-member well-being and experiences associated with delirium during and after ICU stay with the ICU Memory Tool (ICU-MT (16)), Delirium Experience Questionnaire (DEQ (17)) and Caregiver Strain Index (CSI (26)).
- Posttraumatic stress syndrome (PTSS) in participants and family-members with the Impact of Event Scale – Revised (IES-R)(27).

12 months after randomization

- Cognitive outcomes with a detailed cognitive assessment battery of validated and repeatable measures of general cognition, memory, language, processing speed, attention and executive functioning and mood (Montreal Cognitive Assessment [MOCA](18), Rey Auditory Verbal Learning Test(19), Semantic fluency(20), Digit Span [WAIS-IV](21), Trail making tests A and B(22), Boston naming Test [short version](23), Hospital Anxiety and Depression Scale [HADS](24)).
- Functional outcomes and quality of life (Short Form-36 [SF-36](25)).

For peer review only

- Mortality rate

A cost-effectiveness analysis will be performed in collaboration with the Department of Health Policy and Management of Erasmus University Rotterdam (see Appendix 2 for more detailed explanation).The tools for the secondary outcomes are mentioned in Table 1 with overview of timing of assessments.

Treatment of subjects

<u>Investigational product:</u>

Name: Haldol (haloperidol)

Mechanism: butyrophenone-derived anti-psychotic with mainly dopamine-2 receptor antagonistic properties

Placebo consists of sodium chloride for injection. Medical staff, patients and family will be blinded to the product containing haloperidol/placebo.

<u>Summary of findings from clinical studies and of known and potential risks and benefits</u>*:*

See: Summary of Product Characteristics (SPC) in Appendix 3 and Systematic Review (Appendix 4).

<u>Dosages, dosage modifications and method of administration</u>*:*

The following dosing scheme will be used: start with haloperidol/placebo (further called: “study drug”) 2.5mg IV q8h (because of delirium screening once every 8-hour shift) and increase to a maximum dose of 5mg IV q8h when delirium persists during the next 8-hour shift. Doses will be reduced (50% of dose) in the very old elderly (age ≥ 80 years). The study drug dose will be decreased (when dosage is 5mg IV q8h) or stopped (when dose is 2.5mg IV q8h) when delirium has resolved (or is un-assessable due to coma) for the next 24 hours (implying: three consecutive delirium assessments during three shifts). Dosages can be lowered also at the discretion of the treating physician in case of evident rigidity, which is in

For peer review only

line with current routine practice. Standard clinical practice for the administration of haloperidol will be followed.

<u>Description and justification of route of administration and dosage</u>*:*

Administration of the study intervention via the IV (versus the oral or enteral) route is the most feasible in critically ill patients – a population where gastrointestinal dysfunction is prevalent and haloperidol absorption (i.e., bioavailability) could be compromised. The dose of haloperidol or placebo equivalent to be used in the study is based on the following consideration: 1. PK/PD; 2. Efficacy and 3. Safety. A (pilot) study in Erasmus Medical Center (n=14 critically ill patients, abstract presented at European Society of Intensive Care Medicine 2016) showed no adverse events (e.g. no QTc > 500ms), low serum levels (1.5-2.2μg/L) and no clear relation between serum level and delirium resolution with haloperidol dosages up to 2mg IV q8h (or: 3 x 2mg IV). A feasibility trial of haloperidol for ICU delirium (MIND-trial (8)) that used an average total daily dosage of 15 mg orally found higher serum levels (interquartile range 2.85-5.8 μg/L). No differences were found in QTc prolongation between treatment groups and placebo in this trial. None of these trials demonstrated clinically important safety concerns associated with haloperidol administration. Finally, a recently published trial of haloperidol for ICU delirium using haloperidol/placebo 10mg IV q12h, did not report any safety issues, using a QTc cut-off for safety of 550ms, which may be regarded an indirect signal that such dosages are feasible and safe (28).The maximum dose of haloperidol of up to 5mg IV q8h was further chosen because a previous Dutch guideline advocating the use of haloperidol recommended an IV haloperidol treatment dose of up to 20 mg/24h period (29). In our protocol, we chose q8h dosing (titrated up to 15mg daily) given the greater potential susceptibility of critically ill adults to the side effects of haloperidol, and the fact that this dosage is in line with existing haloperidol delirium protocols in several of the participating ICUs.

<u>Patient assessments:</u>

For peer review only

For peer review only - http://bmjopen.bmj.com/site/about/guidelines.xhtml

Rigidity will be monitored with the Simpson-Angus scale (15) and the Barnes Akathisia Rating Scale (30) (see “Secondary study endpoints”) for study purposes only. The QTc interval will be measured daily before the administration of the second daily (afternoon) dose using a 12-lead EKG. When the QTc interval is found to be prolonged (> 500ms or an increase from baseline (=at randomisation) of ≥ 60ms (31, 32)), all non-study medications having the potential to prolong the QTc will be held if clinically feasible. A Standard Operating Procedure (S.O.P.) lists the drugs known to prolong the QTc. Eight hours later, if QTc prolongation persists, study medication will be held or tapered according to the S.O.P. and only resumed when the EKGs (evaluation frequency increased to q8h in this situation) reveal QTc prolongation to have dissipated.

General medical management at participating ICUs:

In the six original participating ICUs, institutional delirium guidelines, based on the 2013 PAD guidelines and a Dutch ICU delirium guideline, were rigorously implemented over a three-year period (2012 to 2015) (6, 33, 34). During the inclusion period of the current trial, spot-checks will be performed by members of the investigative team at each center to confirm delirium screening accuracy, as a quality-of-assessments measure and these will be documented in a qualitative manner.

Preparation and labelling of Investigational Medicinal Product:

Preparation and labelling will be done by the trial pharmacist (“Apotheek A15”) according to GMP guidelines. Apotheek A15 is certified for these procedures. Trial medication will be dispensed to the pharmacies of the trial sites by the Hospital Pharmacy of Erasmus MC. See Appendix 5 for a description of the drug accountability.

Escape medication:

Knowing that half the subjects will be administered placebo, we anticipate two issues may affect the clinical management of enrolled patients: 1) agitation and 2) hallucinations.

For peer review only - http://bmjopen.bmj.com/site/about/guidelines.xhtml

Agitation management will be based on the following principles: a) treat pain first with opioids; b) use alpha-2 agonist for agitation that either persists or is not caused by pain; c) GABA agonists (e.g. benzodiazepines or propofol) are discouraged, but can be used on a short-term basis for the treatment of severe agitation (RASS ≥ 2) that cannot be effectively managed by other means.

Hallucination management will be based on the following principles: a) pharmacological treatment may be withheld if the patient indicates they are not in distress; b) for a patient in distress, a low-dose atypical antipsychotic (e.g., quetiapine 12.5mg q8h) may be administered on a short-term basis until the distress resolves.

Because of the pragmatic design of this trial, within these boundaries, the treatment and dose of escape medication is left to the treating physician, since these are part of routine practice. However, before start of randomisation these management principles for agitation and hallucination will be thoroughly implemented first with the help of detailed S.O.P.'s to enhance uniformity in participating centres. Adherence to escape medication regimens will be closely monitored. Open-label haloperidol administration is strongly discouraged during the trial but can be used if the ICU team considers it necessary for acute breakthrough delirium symptoms that cannot be managed within the management boundaries outlined above. Open-label haloperidol will be documented.

Randomisation, blinding and treatment allocation

Legal representatives of eligible patients (when the patient is sedated or otherwise temporarily unable to consent) or the patient him-/herself will be asked for informed consent shortly after admission when the patient has no delirium, or as soon as possible after admission when the patient already has delirium. Appendix 6 contains an example of the patient consent form. In this study the presence of delirium will be considered to be confirmed when the beside nurse deemed the patient to have delirium based on assessment with the ICDSC or CAM-ICU, given the previous large-scale implementation project (33).

For peer review only

Delirious patients who fulfil all inclusion but no exclusion criteria, and for whom written informed consent has been obtained (as recorded in medical file), will be randomised. Randomisation coordination and start of a new Case Record Form (CRF) will be guided by the Electronic Data Capture (EDC) system of ALEA, constructed by the Clinical Trial Center (CTC) of the Erasmus Medical Center and calibrated with the coordinating (Erasmus MC) and local pharmacies. We will randomise the recruited patients using a block design of 8 patients in one block, and one block is assigned to a center. We will have 8 batches (numbered 1 through 8) of treatment and placebo, with 4 batches of placebo and 4 treatment (haloperidol). Each block will have a random assignment of 8 batch numbers, having four placebo and four haloperidol patients included (a combination of 1 to 4 and 5 to 8 in random order). After 8 patients are included in the study (i.e., a block is full), a new block will be assigned to a center.

Upon randomisation, the study drug with the corresponding randomisation kit number 1-8 (based on 8 medication batches consisting of either haloperidol or placebo) will be obtained from the hospital pharmacy of each participating ICU. Each box from a batch/kit contains 10 ampules (5mg/1ml) of haloperidol or placebo. If all ampules are used, a new box from the same medication kit number with 10 ampules will be used. Study drugs are administered on prescription in the electronic patient data management system (PDMS) and are double-checked by ICU nurses before administration, which is similar to regular practice. Furthermore, the kit number was noted upon randomisation in the medical file and the kit number could be retrieved at any time from the PDMS after first prescription upon randomisation.

Blinding of the medication will be performed by the pharmacy, based on a randomisation list that will be generated electronically through a randomisation module in the EDC system of ALEA. Randomisation will be stratified per study center (i.e. equal number of patients in both study groups, see “statistical analysis” paragraph). Only the involved pharmacists and the trial statistician are aware of the contents of each medication kit. Only the local (site) pharmacists are able to unblind study treatment of a patient in case of an

For peer review only - http://bmjopen.bmj.com/site/about/guidelines.xhtml

emergency. Except for the hospital's pharmacist responsible for the randomisation list, all other involved personnel with the study, caregivers, patients or their representatives will remain unaware of the treatment groups until the time of Database Lock. The Unblinding procedure is specified in Appendix 7.

Follow-up procedures will be performed according to designated S.O.P.'s. When possible and preferred by patients or families, questionnaires will be sent or visits planned at home when possible, e.g. for incapacitated participants.

Withdrawal of individual subjects

Subjects can leave the study at any time for any reason if they wish to do so without any consequences. The investigator can decide to withdraw a subject from the study for urgent medical reasons.

Follow-up of subjects withdrawn from treatment

Data of withdrawn patients will remain in the database for statistical analysis purposes but will not be subject to follow-up. When patients specifically withdraw their consent for usage of their data, these data will be removed from the database and excluded from all analyses.

Premature termination of the study

The sponsor may decide to terminate the study prematurely based on the following criteria:

- There is evidence of an unacceptable risk for study patients (i.e. safety issue)
- There is reason to conclude that continuation of the study cannot serve a scientific purpose following confirmation of the Data Safety Monitoring Board (DSMB)
- The DSMB recommends to end the trial based on viable arguments other than described above.

The following stopping rules have been determined by the DSMB and have been laid down in a DSMB charter:

For peer review only - http://bmjopen.bmj.com/site/about/guidelines.xhtml

- Early stopping of one individual participant, for example, to clear benefit or harm of a treatment or the occurrence of serious adverse reactions or events in one patient. In this case de-blinding of this single patient may be necessary.
- Stopping of the trial as a whole to clear benefit or harm of a treatment or the occurrence of serious adverse reactions or events. As a result, further patient enrolment will be stopped. Deblinding may be necessary for all patients.

Reasons to stop the study include:

- Advice to do so from DSMB
- Interim analysis shows a significant benefit difference between the treatment groups which will not be expected to change after inclusion of all subject as per the power analysis.

SUSAR's are not expected due to the vast experience in clinical practice with the study drug (haloperidol).

If the study is terminated the Medical Ethics Committees of all participating hospital and the CCMO will be notified.

Safety reporting

AEs, SAEs and SUSARs:

Adverse events (AEs)

Adverse events are defined as any undesirable experience occurring to a subject during the study, whether or not considered related to the investigational product. Since patients admitted to an ICU are critically ill and present with many AEs, only possible adverse drug related events (on days of study drug administration: prolonged QTc by EKG, muscle rigidity and associated movements disorders [Simpson Angus Scale]) as indicated by the subject or observed by the investigator or his staff occurring from the date of randomisation until 14

For peer review only

For peer review only - http://bmjopen.bmj.com/site/about/guidelines.xhtml

days later or discharge from ICU or death (whichever comes first), will be recorded in the CRF. In addition, the following AEs will be assessed daily during 14 days after randomisation: epilepsy, tachycardia, hypotension (not explained otherwise), hepatic dysfunction (not explained otherwise), leucopenia (not explained otherwise), bronchospasms (not explained otherwise).

Serious adverse events (SAEs)

A SAE is any untoward medical occurrence or effect, occurring during the 14-day study period at the ICU, that (the SAEs for the purpose of the study are shown in *Italics* per item)

- results in death;
  - *death will always be reported as an SAE*
- is life threatening (at the time of the event);
  - *ventricular arrhythmia or malignant neuroleptic syndrome*
- requires hospitalisation or prolongation of existing inpatients' hospitalisation;
  - *Not to be expected; only applicable when the site investigator is able to explicitly show a relationship*
- results in persistent or significant disability or incapacity;
  - *Not to be expected; only applicable when the site investigator is able to explicitly show a relationship*
- is a congenital anomaly or birth defect; (*Not applicable*) or
- any other important medical event that did not result in any of the outcomes listed above due to medical or surgical intervention but could have been based upon appropriate judgement by the investigator.

Statistical analysis

Primary and secondary study parameter(s):

Statistical analysis will be done according to intention-to-treat-principle. All randomised participants will be included. The primary outcome is DCFDs, defined as the number of days

For peer review only

in the first 14 days after randomisation during which the patient is alive without delirium and not in coma from any cause (7). Patients who are discharged before the 14 day study period has ended, will be recorded as delirium and coma-free after discharge (8, 35). Additionally, we will assume all patients who died within 14 days after randomisation to have 0 delirium and coma free days (7). Differences between DCFDs between the haloperidol group and placebo group will be analyzed using Poisson regression analysis, with adjustment for differences in baseline characteristics between treatment groups (when present) and for the different centers. We will collect data with regards to baseline demographics: age, sex, admission diagnosis category, APACHE II and APACHE IV, SOFA, ICU days before study entry and pre-admission delirium duration in participants with delirium on admission. Pre-defined sub-analyses will include efficacy stratified by 1) agitated, mixed-type or hypoactive delirium; 2) the presence of hallucinations or delusions; 3) delirium severity (based on ICDSC score: low delirium severity = mean ICDSC score of 4 to 5; medium delirium severity = mean ICDSC score 5 to 7; or high delirium severity = ICDSC score 7 to 8); and 4) sedation-related, hypoxic, metabolic or septic delirium. For cognitive and functional outcomes assessed with designated test-batteries, non-parametric or parametric tests will be used depending on normality of scaled test-results. Mortality risk will be assessed as a binary end-point. A more detailed statistical analysis plan, to be drawn up before Data Base Lock, will be drafted for publication separately.

For peer review only

Interim analysis:

Pre-planned interim analyses will be performed at 1/3 and 2/3 of the trial's course (first analysis ideally estimated at 6 months after start of trial), as determined by the DSMB charter or otherwise when the DSMB requests it.

Sample size calculation

To achieve statistically significant results with ($p<.05$) with a power of 90% and a true treatment difference of one day for the primary outcome (from 3.2 DCFDs in the placebo

For peer review only - http://bmjopen.bmj.com/site/about/guidelines.xhtml

group to 4.2 in the haloperidol group, SD in both groups is equal to 4.2), 371 patients are needed in each group (n=742). These estimates are derived from the previous implementation study, which included 4727 patients in three 4-month periods in the same six participating ICUs and found delirium incidence of 27% (and increase of DCFDs from 60% to 70%)(33). Consequently, presuming an informed consent rate of 40%, we need 18-months to encounter 1900 patients with a newly diagnosed delirium to include the required 742 patients. Because of estimated work-load due to follow-up visits, including e.g. neurocognitive testing, we propose to select a convenience sample of 2/3 of ICU survivors (estimated around 400 of 575 survivors) as a random sample for the cognitive, functional and secondary outcome variables.

Patient and Public Involvement

During the design and conduct of the study we involved two ex-ICU patients as patient-perspective representatives. The primary research question, its outcome measures, and the burden of the intervention have been assessed and found relevant by these patient-representatives. The role and tasks of the patient-representatives for the study have been detailed as: 1) to help select meaningful assessment-tools of patient and family experiences during and after ICU stay, 2) act as liaison between the study management team and the Dutch foundation “Family and patient Centered Intensive Care” (FCIC; one representative is a formal representative for FCIC), 3) act as members of the Stakeholders group to provide advice on the study contents, execution and course at on a regular basis to ensure the patient and family perspective, 4) advise on the contents of the Patient Information Form (PIF) and the informed consent procedure, 5) advise on ways to minimise loss to follow-up for the functional and cognitive outcome assessments, 6) advise on contents and organisation of symposia during the study on delirium and its consequences with the aim to better inform participants of the study and their family members and maximize their involvement, 7) advise on the contents of the supporting website of the trial. Study

For peer review only

For peer review only - http://bmjopen.bmj.com/site/about/guidelines.xhtml

participants will be informed about the most important results of the trial, either by post or symposium, when they indicate this on the informed consent letter.

ETHICS AND DISSEMINATION

The study has been approved by the Medical Ethics Committee of the Erasmus University Medical Centre Rotterdam (MEC2017-511) and the Institutional Review Boards of participating sites. The study will be conducted according to the principles of the Declaration of Helsinki (version, date, see for the most recent version: www.wma.net) and in accordance with the Medical Research Involving Human Subjects Act (WMO) and other guidelines, regulations and Acts.

For peer review only

Recruitment and consent

Recruitment of eligible patients will be done upon admission. Informed consent for *possible* participation (i.e. only when participants develop delirium at the ICU) will be obtained from subjects who are not expected to leave the ICU within the first 24 hours after admission and are not yet delirious. The informed consent will be obtained from the patient or (if the patient is unable to consent) from patient’s representative. This procedure of prior request for informed consent will facilitate randomisation when the patient indeed develops delirium, because randomisation can then be performed 24/7 since informed consent is already obtained and delirium often surfaces during the evening and night when obtaining informed consent is difficult. The informed consent procedure will be clearly delineated from the randomisation procedure. Importantly, when a patient with prior informed consent develops delirium and can thus be randomised, still a pre-randomisation check with regard to in- and exclusion criteria will be performed to confirm that the patient fulfils the inclusion, and not the exclusion criteria (because this may change over time). A team of dedicated research and ICU nurses and physicians (local PI, PhD student, PI, post-doc) will be trained to perform the informed consent procedures and help with the randomisations. Moreover, a 24/7 study consultation telephone number will be opened to help with problems or question during the

For peer review only - http://bmjopen.bmj.com/site/about/guidelines.xhtml

study. A second type of randomisation concerns patients who are delirious upon admission to ICU. These patients' next-of-kin will be asked to grant permission to participate by means of informed consent when they are legally representative for the patients and the patient has no contraindications. After informed consent is obtained, the patient can be randomised.

REFERENCES

1. Salluh JI, Soares M, Teles JM, Ceraso D, Raimondi N, Nava VS, et al. Delirium epidemiology in critical care (DECCA): an international study. Crit Care. 2010;14(6):R210.
2. Ely EW, Shintani A, Truman B, Speroff T, Gordon SM, Harrell FE, Jr., et al. Delirium as a predictor of mortality in mechanically ventilated patients in the intensive care unit. JAMA. 2004;291(14):1753-62.
3. Pandharipande PP, Girard TD, Ely EW. Long-term cognitive impairment after critical illness. N Engl J Med. 2014;370(2):185-6.
4. Milbrandt EB, Deppen S, Harrison PL, Shintani AK, Speroff T, Stiles RA, et al. Costs associated with delirium in mechanically ventilated patients. Crit Care Med. 2004;32(4):955-62.
5. Reade MC, Eastwood GM, Bellomo R, Bailey M, Bersten A, Cheung B, et al. Effect of Dexmedetomidine Added to Standard Care on Ventilator-Free Time in Patients With Agitated Delirium: A Randomized Clinical Trial. JAMA. 2016;315(14):1460-8.
6. Devlin JW, Skrobik Y, Gelinas C, Needham DM, Slooter AJC, Pandharipande PP, et al. Clinical Practice Guidelines for the Prevention and Management of Pain, Agitation/Sedation, Delirium, Immobility, and Sleep Disruption in Adult Patients in the ICU. Crit Care Med. 2018;46(9):e825-e73.
7. Page VJ, Ely EW, Gates S, Zhao XB, Alce T, Shintani A, et al. Effect of intravenous haloperidol on the duration of delirium and coma in critically ill patients (Hope-ICU): a randomised, double-blind, placebo-controlled trial. Lancet Respir Med. 2013;1(7):515-23.
8. Girard TD, Pandharipande PP, Carson SS, Schmidt GA, Wright PE, Canonico AE, et al. Feasibility, efficacy, and safety of antipsychotics for intensive care unit delirium: the MIND randomized, placebo-controlled trial. Crit Care Med. 2010;38(2):428-37.
9. Barnes-Daly MA, Phillips G, Ely EW. Improving Hospital Survival and Reducing Brain Dysfunction at Seven California Community Hospitals: Implementing PAD Guidelines Via the ABCDEF Bundle in 6,064 Patients. Crit Care Med. 2017;45(2):171-8.
10. Al-Qadheeb NS, Balk EM, Fraser GL, Skrobik Y, Riker RR, Kress JP, et al. Randomized ICU trials do not demonstrate an association between interventions that reduce delirium duration and short-term mortality: a systematic review and meta-analysis. Crit Care Med. 2014;42(6):1442-54.
11. Collet MO, Caballero J, Sonneville R, Bozza FA, Nydahl P, Schandl A, et al. Prevalence and risk factors related to haloperidol use for delirium in adult intensive care patients: the multinational AID-ICU inception cohort study. Intensive Care Med. 2018;44(7):1081-9.
12. Jorm AF, Jacomb PA. The Informant Questionnaire on Cognitive Decline in the Elderly (IQCODE): socio-demographic correlates, reliability, validity and some norms. Psychol Med. 1989;19(4):1015-22.
13. Hodgson C, Needham D, Haines K, Bailey M, Ward A, Harrold M, et al. Feasibility and inter-rater reliability of the ICU Mobility Scale. Heart Lung. 2014;43(1):19-24.
14. Richards KC, O'Sullivan PS, Phillips RL. Measurement of sleep in critically ill patients. J Nurs Meas. 2000;8(2):131-44.
15. Simpson GM, Angus JW. A rating scale for extrapyramidal side effects. Acta Psychiatr Scand Suppl. 1970;212:11-9.

For peer review only

For peer review only - http://bmjopen.bmj.com/site/about/guidelines.xhtml

16. Jones C, Griffiths RD, Humphris G, Skirrow PM. Memory, delusions, and the development of acute posttraumatic stress disorder-related symptoms after intensive care. Crit Care Med. 2001;29(3):573-80.
17. Breitbart W, Gibson C, Tremblay A. The delirium experience: delirium recall and delirium-related distress in hospitalized patients with cancer, their spouses/caregivers, and their nurses. Psychosomatics. 2002;43(3):183-94.
18. Nasreddine ZS, Phillips NA, Bedirian V, Charbonneau S, Whitehead V, Collin I, et al. The Montreal Cognitive Assessment, MoCA: a brief screening tool for mild cognitive impairment. J Am Geriatr Soc. 2005;53(4):695-9.
19. Bean J. Rey Auditory Verbal Learning Test, Rey AVLT. In: Kreutzer JS, DeLuca J, Caplan B, editors. Encyclopedia of Clinical Neuropsychology. New York, NY: Springer New York; 2011. p. 2174-5.
20. Ledoux K, Vannorsdall TD, Pickett EJ, Bosley LV, Gordon B, Schretlen DJ. Capturing additional information about the organization of entries in the lexicon from verbal fluency productions. J Clin Exp Neuropsychol. 2014;36(2):205-20.
21. Wechsler D. Wechsler adult intelligence scale–Fourth Edition (WAIS–IV). San Antonio, TX: NCS Pearson. 2008;22:498.
22. Reitan RM, Wolfson D. The Halstead–Reitan neuropsychological test battery for adults—Theoretical, methodological, and validational bases. Neuropsychological assessment of neuropsychiatric and neuromedical disorders. 2009;1:36.
23. Roth C. Boston Naming Test. In: Kreutzer JS, DeLuca J, Caplan B, editors. Encyclopedia of Clinical Neuropsychology. New York, NY: Springer New York; 2011. p. 430-3.
24. Zigmond AS, Snaith RP. The hospital anxiety and depression scale. Acta Psychiatr Scand. 1983;67(6):361-70.
25. Ware JE, Jr., Sherbourne CD. The MOS 36-item short-form health survey (SF-36). I. Conceptual framework and item selection. Med Care. 1992;30(6):473-83.
26. Robinson BC. Validation of a Caregiver Strain Index. J Gerontol. 1983;38(3):344-8.
27. Weiss DS, Marmar CR, Wilson JP, Keane TM. Assessing psychological trauma and PTSD. The Impact of Events Scale—Revised. 1997;19:399-411.
28. Girard TD, Exline MC, Carson SS, Hough CL, Rock P, Gong MN, et al. Haloperidol and Ziprasidone for Treatment of Delirium in Critical Illness. The New England journal of medicine. 2018;379(26):2506-16.
29. Nederlandse Vereniging voor Psychiatrie. Richtlijn delirium. Amsterdam: Dries van Ingen, Uitgeverij Boom; 2004. p. 1-127.
30. Barnes TR. The Barnes Akathisia Rating Scale--revisited. J Psychopharmacol. 2003;17(4):365-70.
31. Fongemie JM, Al-Qadheeb NS, Estes NA, 3rd, Roberts RJ, Temtanakitpaisan Y, Ruthazer R, et al. Agreement between ICU clinicians and electrophysiology cardiologists on the decision to initiate a QTc-interval prolonging medication in critically ill patients with potential risk factors for torsade de pointes: a comparative, case-based evaluation. Pharmacotherapy. 2013;33(6):589-97.
32. Tisdale JE, Jaynes HA, Kingery JR, Overholser BR, Mourad NA, Trujillo TN, et al. Effectiveness of a clinical decision support system for reducing the risk of QT interval prolongation in hospitalized patients. Circ Cardiovasc Qual Outcomes. 2014;7(3):381-90.
33. Trogrlic Z, van der Jagt M, Lingsma H, Gommers D, Ponssen HH, Schoonderbeek JFJ, et al. Improved Guideline Adherence and Reduced Brain Dysfunction After a Multicenter Multifaceted Implementation of ICU Delirium Guidelines in 3,930 Patients. Crit Care Med. 2019.
34. Barr J, Fraser GL, Puntillo K, Ely EW, Gelinas C, Dasta JF, et al. Clinical practice guidelines for the management of pain, agitation, and delirium in adult patients in the intensive care unit. Crit Care Med. 2013;41(1):263-306.
35. van den Boogaard M, Slooter AJC, Bruggemann RJM, Schoonhoven L, Beishuizen A, Vermeijden JW, et al. Effect of Haloperidol on Survival Among Critically Ill Adults With a High Risk of Delirium: The REDUCE Randomized Clinical Trial. JAMA. 2018;319(7):680-90.

For peer review only - http://bmjopen.bmj.com/site/about/guidelines.xhtml

AUTHORS' CONTRIBUTIONS

MJ developed the study protocol, edited and approved the final version, and was responsible for funding and supervising the study coordination. ZT contributed to the protocol development and study implementation. LS is responsible for study coordination. WR assisted with statistical analysis and NH assisted with pharmacological coordination. RO, AS, HP and JD were involved in the study design and protocol development. All authors (LS, ZT, JD, RO, HP, AS, NH, WR, DG, MJ) contributed to the development and refinement of this study protocol. They have read and approved the final version of the protocol.

Acknowledgements

EuRIDICE study group authors:

Local principal investigators: M. van den Boogaard (15), A.J.B.W. Brouwers (7), J.A. Lens (9), B.J.M. van der Meer (8) , H. Ponssen (4), F.J. Schoonderbeek (10), K.S. Simons (16).

Local research nurses: E. Berger (7), A. Bouman (16), M. Campo (8), D. van Duijn (1), H. Embden – van Donk (10), D. van de Graaf (9), E. Hoogendoorn (4), P. Ormskerk (1), N. Roovers (15), E. Toscano (8), A. Vileito (1), T. van Zuylen (16).

All involved local principal investigators (MB, AB, BM, JL, EK, FS, KS) and research nurses (EB, AB, DD, HE, DG, PO, NR, AV, MC, ET, EH, TZ) have facilitated visits at their site, will be involved in executing the protocol at their sites and will be responsible for patient recruitment and data collection in their hospitals, along with follow up of study patients.

Other members involved: C. Exler (11), E. van den Berg (12), J. van Meeteren (13), M. Koopmanschap (14).

Patient representatives: I. Nutma and E. Kuijper.

For peer review only

1) Erasmus MC-University Medical Center, Department of Intensive Care Adults, Rotterdam, The Netherlands, Netherlands

4) Albert Schweitzer Hospital, Department of Intensive Care, Dordrecht, Netherlands

7) Franciscus Gasthuis, Department of Intensive Care, Rotterdam, Netherlands

8) Maasstad Hospital, Department of Intensive Care, Rotterdam, Netherlands

9) IJsselland Hospital, Department of Intensive Care, Capelle aan den IJssel, Netherlands

10) Ikazia Hospital, Department of Intensive Care, Rotterdam, Netherlands

11) Erasmus MC-University Medical Center, Department of Pharmacy, Rotterdam, The Netherlands, Netherlands

12) Erasmus MC-University Medical Center, Department of Neuropsychology, Rotterdam, The Netherlands, Netherlands

13) Erasmus MC-University Medical Center, Department of Rehabilitation Medicine, Rotterdam, The Netherlands, Netherlands

14) Erasmus School of Health Policy & Management, Department of Health Economics and HTA, Rotterdam, The Netherlands, Netherlands

15) Radboud University Medical Center, Department of Intensive Care Medicine, Nijmegen, The Netherlands

16) Jeroen Bosch Hospital, Department of Intensive Care Medicine, 's-Hertogenbosch, The Netherlands.

FUNDING STATEMENT

A grant has been provided by ZonMw – The Netherlands Organisation for Health Research and Organisation. ZonMw project number: 848041001. This funding source had no role in the design of this study and will not have any role during its execution, analyses, interpretation of the data, or decision to submit results.

COMPETING INTERESTS STATEMENT

The authors declare that they have no competing interests.

For peer review only - http://bmjopen.bmj.com/site/about/guidelines.xhtml

For peer review only

LIST OF ABBREVIATIONS

**ABR**	**ABR form, General Assessment and Registration form, is the application form that is required for submission to the accredited Ethics Committee (In Dutch, ABR = Algemene Beoordeling en Registratie)**
**AE**	**Adverse Event**
**AR**	**Adverse Reaction**
**CA**	**Competent Authority**
**CAM-ICU**	**Confusion Assessment Method for the ICU**
**CCMO**	**Central Committee on Research Involving Human Subjects; in Dutch: Centrale Commissie Mensgebonden Onderzoek**
**CV**	**Curriculum Vitae**
**DSMB**	**Data Safety Monitoring Board**
**EU**	**European Union**
**EudraCT**	**European drug regulatory affairs Clinical Trials**
**EKG**	**Electrocardiography**
**GCP**	**Good Clinical Practice**
**IB**	**Investigator's Brochure**
**IC**	**Informed Consent**
**ICDSC**	**Intensive Care Delirium Screening Checklist**
**ICU**	**Intensive Care Unit**
**IMP**	**Investigational Medicinal Product**
**IMPD**	**Investigational Medicinal Product Dossier**
**METC**	**Medical research ethics committee (MREC); in Dutch: medisch ethische toetsing commissie (METC)**
**PAD**	**Pain, agitation and delirium**
**RCT**	**Randomized Controlled Trial**
**(S)AE**	**(Serious) Adverse Event**
**S.O.P.**	**Standard Operating Procedure**
**SPC**	**Summary of Product Characteristics (in Dutch: officiële productinfomatie IB1-tekst)**
**Sponsor**	**The sponsor is the party that commissions the organisation or performance of the research, for example a pharmaceutical**

For peer review only - http://bmjopen.bmj.com/site/about/guidelines.xhtml

	**company, academic hospital, scientific organisation or investigator. A party that provides funding for a study but does not commission it is not regarded as the sponsor, but referred to as a subsidising party.**
**SUSAR**	**Suspected Unexpected Serious Adverse Reaction**
**TdP**	**Torsade de Pointes**
**Wbp**	**Personal Data Protection Act (in Dutch: Wet Bescherming Persoonsgevens)**
**WMO**	**Medical Research Involving Human Subjects Act (in Dutch: Wet Medisch-wetenschappelijk Onderzoek met Mensen)**

682

683 **Table 1. Overview of timing of assessments, including required time investment per**
684 **visit/questionnaire.**

**Moment (months)**	**Neurocognitive tests**	**Patient and family experiences (time in min.)**	**Functional outcomes (SF-36)**	**Cost effectivity questionnaires (EQ-5D-5L, iMTA MCQ, iMTA PCQ)**	**Other**
**Enrolment**					Informed consent, IQCODE-N, pregnancy test (if applicable), EKG.
**ICU study period (3x / day)**					CAM-ICU / ICDSC, RASS
**ICU study period (once daily)**					IMS, RCSQ. Only when on study medication:

For peer review only - http://bmjopen.bmj.com/site/about/guidelines.xhtml

					EKG,<br>Simpson<br>Angus Scale.
**0**<br>**(discharge from hospital)**		Patient: ICU-MT (15) +<br>DEQ (15)<br>Family: DEQ (2)			
**1**				30 min.	
**3**	45-60 min.	Patient: IES-R (5) + ICU-MT (15) + DEQ (15)<br>Family: IES-R (5) + CSI (5) + DEQ (2)	10 min.	30 min.	
**6**				30 min.	
**12**	45-60 min.		10 min.	30 min.	

IQCODE-N = Informant Questionnaire on Cognitive Decline in the Elderly – Dutch version

EKG = Electrocardiography

Neurocognitive tests: Montreal Cognitive Assessment (MOCA), Rey Auditory Verbal Learning Test, Semantic fluency, Digit Span (WAIS-IV), Trailmaking tests A and B, Boston naming Test (short version), Hospital Anxiety and Depression Scale (HADS)

IMS = ICU Mobility Scale, measures mobility during ICU admission

RCSQ = Richards-Campbell Sleep Questionnaire, measures quality of sleep

Simpson Angus Scale = measures muscle rigidity and other associated movements disorders

ICU-MT= ICU-Memory Tool, assesses the experience and memories of ICU admission

DEQ= Delirium Experience Questionnaire, measures experiences linked to delirium

IES-R = Impact of Event Scale Revised, assesses distress linked to a traumatic experience (i.e. experiencing delirium)

For peer review only - http://bmjopen.bmj.com/site/about/guidelines.xhtml

For peer review only

CSI = Caregiver Strain Index, assesses the strain experienced by the caregiver

SF-36 = Short Form-36, measures the health-related quality of life

EQ-5D-5L = assesses the general health status

iMTA MCQ = instituut Beleid & Management Gezondheidszorg Medical Consumption Questionnaire (health care use)

iMTA PCQ = instituut Beleid & Management Gezondheidszorg Productivity Cost Questionnaire (productivity costs)

With the exception of the neurocognitive tests, all above mentioned tools are questionnaires that can be administered at home. Real life visits only need to be paid in order to perform the neurocognitive tests.

For peer review only

For peer review only - http://bmjopen.bmj.com/site/about/guidelines.xhtml

Appendix 1: Participating hospitals

Erasmus MC Rotterdam

Albert Schweizer Hospital Dordrecht

Maasstad Hospital Rotterdam

IJsselland Hospital Capelle aan den IJssel

Ikazia Hospital Rotterdam

Franciscus Gasthuis Rotterdam

As of July 2019 two additional ICUs have started recruitment:

Jeroen Bosch Hospital, 's-Hertogenbosch

Radboud University Medical Center, Nijmegen

For peer review only

Appendix 2: Economic evaluation

ECONOMIC EVALUATION

General considerations

The primary economic analysis will be a trial-based **cost-utility analysis** from a societal and a healthcare perspective. This analysis will be performed according to the Dutch guidelines (1, 2). The time horizon will be 12 months after randomisation, in order to take all relevant costs and effects regarding the treatment procedure into account. Additionally, a **cost-effectiveness analysis** performed from a societal and health care perspective will be conducted, using delirium-free and coma-free days as outcomes.

If a difference in quality of life is observed at the end of the follow-up period, we will also perform a **model-based extrapolation** of costs and health benefits up to 5 years, exploring the following scenarios: (1) health benefit remain constant after the follow-up period, (2) health benefits are gradually phased out over the course of the modelling time, (3) health benefits are gradually phased out over the course of the modelling time over the first year after follow-up, (4) health benefits abruptly disappear after the follow-up period, but costs remain until the end of the modelling period.

If treatment with haloperidol leads to better health outcomes at higher costs, or if it leads to worse health outcomes and cost savings, incremental cost-utility and incremental cost-effectiveness ratios will be calculated. These express the additional costs per unit of health gain (QALYs, delirium-free days, coma-free days) or the savings per unit of health forgone. The uncertainty around the estimates will be addressed using bootstrapping for the analysis of costs and effects in the first 12 months, and using probabilistic sensitivity analysis in the extrapolation model.

Cost analysis

Healthcare costs will be calculated based on patient-level data on health-care utilization, which will be collected from hospital databases and questionnaires, to be filled out at regular intervals by patients and/or informal caregivers. Cost categories include medication, screenings, inpatient days, contacts with healthcare providers (GP, outpatient visits, and therapists). The questionnaire will also contain questions about absence from paid work by the patient and informal caregivers.

Costs will be calculated by multiplying resource utilization with the cost per unit of resource. Some unit costs will be taken from the 2016 Dutch Manual for Costing Studies(3), but the costs of inpatient days will be assessed following the micro-costing method, which is based on comprehensive ‘bottom-up’ analyses of the activities of staff and other resources that are used

during those days. Medication prices will be based on the official list prices, including value added tax and increased by a standard prescription reimbursement for the pharmacist. The cost of production loss will be calculated according to the Friction Cost Approach.

Patient outcome analysis

The primary outcome measure in the economic evaluation is the difference in QALYs. The secondary effects are the delirium-free and coma-free days after treatment with haloperidol or placebo. As measuring QALYs in adult critically ill patients is not feasible at baseline, it is not possible to estimate the average number of QALYs for each treatment group. However, assuming that there is no difference at randomisation, it is possible to analyse the difference in quality of life at subsequent measurements in a multilevel regression model. This will enable us to calculate a difference in QALYs between the treatment groups over the total follow-up period, using linear intrapolation. HRQoL will be measured on t=1, 3, 6 and 12 months after randomization using the EQ-5D-5L instrument.

References:

1. Hakkaart-van Roijen L., Tan SS., Bouwmans CAM. Handleiding voor kostenonderzoek, methoden en standaard kostprijzen voor economische evaluaties in de gezondheidszorg. College voor zorgverzekeringen, Geactualiseerde versie 2010.
2. Zorginstituut Nederland. Richtlijn voor het uitvoeren van economische evaluaties in de gezondheidszorg. 2015.
3. Hakkaart-van Roijen L., van der Linden N., Bouwmans CAM., Kanters T., Tan SS. Costing manual: Methodology of costing research and reference prices for economic evaluations in healthcare. 2015.

For peer review only

Appendix 3: Haloperidol SPC

See this weblink: https://db.cbg-meb.nl/IB-teksten/h03185.pdf

For peer review only

Appendix 4: (Semi-) structured review of literature on efficacy and adverse events of haloperidol for delirium in adult critically ill patients

1. Haloperidol as a treatment for ICU delirium

Systematic review of randomised placebo-controlled trials assessing haloperidol for treatment of ICU delirium

Method: A biomedical Information Specialist (BIS) of the Erasmus Medical Center library performed a systematic search aimed at controlled studies on haloperidol for ICU delirium combining the subjects: delirium, ICU and haloperidol, or equivalent terms (see: Appendix for details). No distinction was made in the search between treatment or prevention trials.

Review: Since focus of the EuRIDICE study is on a haloperidol versus placebo comparison, the study selection for this summary is also focused on placebo-controlled haloperidol trials for the treatment of ICU delirium. Systematic reviews from the systematic search are used as a crosscheck to confirm completeness or provide additional insights. The search (total of yielded only 1 study. The MIND trial (2010) was a randomised placebo controlled feasibility, efficacy and safety trial of antipsychotics for ICU delirium in adult mechanically ventilated medical and surgical patients (1). It included three treatment arms (haloperidol, n=35; ziprasidone, n=30 and placebo, n=36) and used a well thought out design (excluding demented patients with a validated tool for cognitive dysfunction, using CAM-ICU as a validated screening tool, a clear protocol with regard to QTc prolongation and study drug dosing, measuring extrapyramidal symptoms with a validated scale and with number of days alive without delirium and coma as the primary outcome (indicating total burden of brain dysfunction, since only assessing delirium days may result in increased coma days and less delirium days being regarded as a – false – improvement). The study used oral haloperidol, no clear sedation protocol aimed at light sedation and crossover antipsychotics were allowed but discouraged. No clear differences were found in the three groups with regard to the primary outcome. Mean haloperidol dose was 15 mg a day but QTc prolongation and extrapyramidal symptoms did not differ between treatment groups. Other medications in this small trial did not differ between groups (propofol, opiates, benzodiazepines). It was concluded that a larger trial would be safe and feasible.

Overview of most recent guidelines' statements on haloperidol as treatment for ICU delirium

Method: Pubmed search on published guidelines including ICU delirium and containing information on

For peer review only

haloperidol. Search terms: guideline, delirium, ICU.

Review: Three recent guidelines were retrieved (2-4). In a Danish guideline (2015) no evidence is stated for pharmacological management(2). A German guideline (2015) advocates symptom-based therapy when delirium screening is positive with haloperidol as a first choice in case of delirium associated with psychotic symptoms only. The “Clinical practice guidelines for the management of pain, agitation, and delirium in adult patients in the intensive care unit” (2013)(3) advocate avoiding ‘antipsychotics’ when risk of torsades de pointes or is present or either baseline QT prolongation or concomitant QT prolonging medication is used. It states that there is no evidence that haloperidol decrease delirium duration, which was perceived as the most relevant issue to address with regard to haloperidol treatment of ICU delirium.

Cochrane review(s)

Method: Search on Cochrane (http://www.cochranelibrary.com) for reviews with search term: ‘delirium’, does not elicit any results pertaining to pharmacological treatment of delirium nor haloperidol.

Review: no Cochrane reviews exist on (ICU) delirium and it’s pharmacological management.

On-going trials

Method: A search for ‘haloperidol’ and ‘delirium’ in the following online trial databases (and including ICU patients); www.trialregister.nl (0 trials); www.clinicaltrials.gov (4 trials).

Review: Four trials were retrieved from www.clinicaltrials.gov. One trial (‘Haloquet’) was not a truly placebo controlled trial because haloperidol was allowed (‘as needed’) in the placebo group and was last updated in 2013 but not published. It consisted of three treatments arms (also quetiapine) and aimed to include a total of 45 patients (and should thus be considered a pilot trial and not an efficacy trial). A second trial enrolled 40 patients and was completed in 2011 but not published. A third trial was a phase-2 safety/efficacy study enrolling 20 patients, last updated in 2007 and not published. The fourth trial (‘The modifying the impact of ICU-associated neurological dysfunction-USA [MIND-USA] study’) is currently recruiting (last verified May 2016 on September 14th). It is a multi-center double blind placebo-controlled trial aiming to enrol 561 patients in three treatment arms: haloperidol, ziprasidone and placebo, by the same research group that did the MIND trial. It includes cognitive and

For peer review only

psychological follow-up at 12 months and is estimated to be completed July 2019. Maximum dose of haloperidol amounts to 10 mg IV q12 hours. Trial design is similar to the EuRIDICE trial, except for the patient experiences and perspective, and the fact that only patients on mechanical ventilation or in shock are included (i.e. the sickest ICU patients). The study protocol has not been published in a peerreviewed journal.

2. Haloperidol to prevent ICU delirium

Systematic review of randomised placebo-controlled trials assessing haloperidol for prevention of ICU delirium; including information from guidelines and Cochrane reviews

Method: A biomedical Information Specialist (BIS) of the Erasmus Medical Center library performed a systematic search aimed at controlled studies on haloperidol for ICU delirium combining the subjects: delirium, ICU and haloperidol, or equivalent terms (see: Appendix for details).

Review: the focus of this section is on randomised placebo-controlled prevention trials of haloperidol for ICU delirium. Three trials were retrieved. One trial included post-operative generally non-critically ill patients (5) and was not further considered for this review. The Hope-ICU trial (2013)(6) was a prophylactic study of haloperidol (2.5mg IV q8h, n=71) versus placebo (n=70) in adult mechanically ventilated ICU patients. The primary end-point of delirium (assessed with CAM-ICU) and coma free days did not differ between groups (5 days in both), but there was a 21% crossover rate with haloperidol in the placebo group. Secondary clinical endpoints such as length of stay at ICU or mortality did not differ but the trial was not powered on these outcomes. Another trial (2016)(7) including mechanically ventilated patients (n=68) with ‘subsyndromal’ delirium (=an Intensive Care Delirium Screening Checklist [ICDSC] score of 1-3 on a scale of 8, where 4 or more is compatible with delirium) used haloperidol 1mg IV q6h but did not find lower rate of progression to full delirium.

3. Haloperidol: adverse events versus treatment effects in the few available trials

The adverse events associated with haloperidol in the three aforementioned (small) trials (one treatment and two prevention trials) did not include QTc prolongation (with a threshold of >500 ms). In the Hope-ICU trial more opiates and sedatives were administered in the placebo-group but alfa-2

For peer review only

agonists were not clearly protocolled, more agitation was present and 26% versus 11% antipsychotics' use in the placebo group. The subsyndromal delirium trial similarly found more agitation in the placebo group.

4. Healthcare perspective

A cost-effectiveness analysis of the Hope-ICU trial found that delirium increased cognitive dysfunction at 6 months and reduced quality of life, suggestive of potential cost-effectiveness of haloperidol (8).

5. Added value of the EuRIDICE trial

Based on this review of available pertinent literature after a thorough BIS-supported systematic search, the proposed trial in this grant application is expected to have important potential additional value:

The indication of haloperidol for ICU delirium will be delineated more clearly by this trial: does it decrease ICU brain dysfunction, associated long-term cognitive, functional and psychological outcomes? Is the intervention cost-effective? Are adverse events associated with haloperidol indeed concerning or actually negligible? Or: has haloperidol become obsolete, now that alternatives have been incorporated into clinical practice, mainly the atypical antipsychotics and alpha-2 agonists (dexmedetomidine and clonidine)? The EuRIDICE trial has a very strong potential to answers all of these questions.

A similar trial as EuRIDICE in the United States is on-going. However, US-based delirium research may not necessarily translate to European/Dutch settings as has been shown before (9), which justifies performing a second large multicentre clinical trial. Moreover, evidence on the pharmacological treatment of delirium is needed because of the lack of trials to date, and the level of evidence and generalizability of the efficacy findings for haloperidol will increase with a second trial. Third, cost-effectiveness of the intervention will be assessed from a healthcare and societal perspective and family and patient experiences will be investigated as important secondary outcomes. Further, we aim to include all critically ill patients, and not just the sickest, i.e. those on mechanical ventilation or in shock.

Existing guidelines and systematic reviews will have to be adapted on the basis of the results this proposed trial.

For peer review only

Acknowledgements:

Gerdien B. de Jonge (MSc), biomedical information specialist, Medical Library, Erasmus MC, is kindly acknowledged for her help in assembling the databases for the systematic review.

References

1. Girard TD, Pandharipande PP, Carson SS, Schmidt GA, Wright PE, Canonico AE, et al. Feasibility, efficacy, and safety of antipsychotics for intensive care unit delirium: The mind randomized, placebo-controlled trial. Crit Care Med. 2010;38(2):428-37.
2. Fonsmark L, Hein L, Nibroe H, Bundgaard H, de Haas I, Iversen S, et al. Danish national sedation strategy. Targeted therapy of discomfort associated with critical illness. Danish society of intensive care medicine (dsit) and the danish society of anesthesiology and intensive care medicine (dasaim). Dan Med J. 2015;62(4):C5052.
3. Barr J, Fraser GL, Puntillo K, Ely EW, Gelinas C, Dasta JF, et al. Clinical practice guidelines for the management of pain, agitation, and delirium in adult patients in the intensive care unit. Crit Care Med. 2013;41(1):263-306.
4. D. A. S. Taskforce, Baron R, Binder A, Biniek R, Braune S, Buerkle H, et al. Evidence and consensus based guideline for the management of delirium, analgesia, and sedation in intensive care medicine. Revision 2015 (das-guideline 2015) - short version. Ger Med Sci. 2015;13:Doc19.
5. Wang W, Li HL, Wang DX, Zhu X, Li SL, Yao GQ, et al. Haloperidol prophylaxis decreases delirium incidence in elderly patients after noncardiac surgery: A randomized controlled trial*. Crit Care Med. 2012;40(3):731-9.
6. Page VJ, Ely EW, Gates S, Zhao XB, Alce T, Shintani A, et al. Effect of intravenous haloperidol on the duration of delirium and coma in critically ill patients (hope-icu): A randomised, double-blind, placebo-controlled trial. Lancet Respir Med. 2013;1(7):515-23.
7. Al-Qadheeb NS, Skrobik Y, Schumaker G, Pacheco MN, Roberts RJ, Ruthazer RR, et al. Preventing icu subsyndromal delirium conversion to delirium with low-dose iv haloperidol: A double-blind, placebo-controlled pilot study. Crit Care Med. 2016;44(3):583-91.
8. Joachim M, Daniel FM, Annalisa C, Timothy A, Xiao BZ, Eugene WE, et al. Health evaluation and cost-effectiveness analysis from a randomized trial of haloperidol in the management of delirium in the critically ill (hope-icu trial). C24 sedation, delirium, and neurocognitive dysfunction in critical care. p. A4022-A.
9. van Eijk MM, van den Boogaard M, van Marum RJ, Benner P, Eikelenboom P, Honing ML, et al. Routine use of the confusion assessment method for the intensive care unit a multicenter study. Am J Respir Crit Care Med. 2011;184(3):340-4.

For peer review only

Appendix 5: Drug Accountability

The study drug will be obtained from the hospital pharmacy of each participating ICU.

The research nurse of each participating ICU will record the number of the box with study drug for each patient in the CRF.

The research nurse of each participating ICU is responsible for retrieving the boxes with study drug. The amount of vials in the boxes will be counted for each patient and will be noted in the CRF. The research nurse will return unused drug to the hospital pharmacy. The hospital pharmacy will destroy the vials with study drug and will also record this (double administration).

The pharmacist or another appropriate individual who is designated should maintain records of the product's delivery to the trial site, the inventory at the site, the use by each patient, problems and irregularities during injection, the maintenance of the blinding, and the return to the pharmacy of unused product(s). These records should include dates, quantities, batch/serial numbers, expiration dates (if applicable), and the unique code numbers assigned to the investigational product(s) and trial patients (if applicable). Investigators should maintain records that document adequately that the patients were provided the doses specified by the protocol and reconcile all investigational product(s).

For peer review only

Appendix 6: Example of the patient consent form

Subject information and consent form for participation in medical scientific research

Effectiveness of haloperidol for the treatment of acute confusion (delirium) in critically ill patients

"Efficacy of haloperidol to decrease the burden of Delirium In adult Critically ill patiEnts (EuRIDICE): a prospective randomised multi-center double-blind placebo-controlled clinical trial"

(note: this Patient Information Letter is a translated version of the original Dutch document. It was Google translated and checked for readability by the study PI and coordinator)

Introduction

Dear Sir / Madam,

You receive this letter because you have been admitted to the Intensive Care Unit and have a chance (about 30%) of developing a delirium (sudden confusion) during admission. We ask you to participate in a medical-scientific study. Participation is voluntary. Your written permission is required to participate. Before you decide whether you want to participate in this study, you will receive an explanation of what the study entails. Please read this information carefully and ask the researcher if you have any questions. You can also ask the independent expert mentioned at the end of this letter for additional information. You can also discuss it with your partner, family or friends.
Further information about participating can be found in the attached brochure "Medical scientific research: general information for the test subject".

1. General information

This research was set up by Erasmus MC Rotterdam and is carried out by doctors and nurses in various hospitals in the Rotterdam region. This study requires a total of 742 subjects from different hospitals in the Rotterdam region. Erasmus MC's medical ethics review committee has approved this study. General information about the approval of research can be found in the brochure "Medical scientific research: general information for the test subject".

2. Purpose of the study

The aim of this study is to examine how safe and effective the drug haloperidol is for the treatment of acute confusion (delirium) in patients admitted to the Intensive Care Unit (ICU).

For peer review only - http://bmjopen.bmj.com/site/about/guidelines.xhtml

Haloperidol has been widely used for many years to treat delirium in ICU patients. However, whether haloperidol can reduce delirium once it has occurred has never been properly investigated. We therefore compare the effects of haloperidol with a placebo. A placebo is a drug without an active substance, a "fake" drug.

3. Background of the study

Delirium (sudden confusion) is a common problem in patients on an ICU. Delirium is associated with an increased risk of death, memory and thinking disorders and a reduced general condition in patients who leave the ICU. A common drug used to treat delirium in ICU patients is haloperidol. This medicine can have a beneficial effect on sudden anxiety and delusions (hallucinations), which often occur with delirium, but can also have side effects. The advantages and disadvantages of treatment with haloperidol have never been properly investigated in a so-called randomized-controlled study.

4. What it means to participate

Examination of eligibility

First we determine whether you can participate. We ask you or your close family about possible memory complaints indicating cognitive dysfunction before your admission to the ICU. When a pregnancy is possible, a pregnancy test is done. If you have memory problems that require further investigation or if you are pregnant, we will tell you and you cannot participate in the study. If you do not want to know if you are pregnant, you cannot participate in this study.

Sometimes during the examination of eligibility or follow-up study we find memory complaints or anxiety or depression complaints that require further medical examination. We will always share these test results with you. Further management of any test results indicating memory issues, anxiety etc, will be done through your own GP. The costs are covered by your own insurance.

Treatment

If you give permission to participate in this study and develop a delirium during admission to the ICU, study medication will be started. We will treat you with study medication for a maximum of 2 weeks. Half of the subjects receive the active agent (haloperidol), the other half the fake agent (placebo). Random selection determines whether you will receive haloperidol or placebo. You, your close relative or family member and all caregivers, such as nurses and the researcher, do not know which group you are in. If it is necessary for your health, it can be looked up.

General information can be found in the brochure "Medical scientific research: general information for the test subject".

Visits and measurements

Data will be collected for the study in the first two weeks. A description of the measurements made for this can be found in Appendix C.

For peer review only - http://bmjopen.bmj.com/site/about/guidelines.xhtml

For the examination, you will need to come to the hospital twice in 12 months after discharge to accurately test for memory and thinking disorders. A visit takes about 1.5 hours. If it is difficult for you to visit the hospital, we will try as much as possible to visit you at your home for the tests. You will also be sent questionnaires by post after discharge from the hospital, after 1, 3, 6 and 12 months after the ICU admission. The questions are about your experiences with and memories of the delirium and ICU admission, how fit you are and what physical limitations there may be. We also send questionnaires to your family member that record how they experienced your delirium and how they experience caring for you.
See appendix C for a schedule with an overview and explanation of the visits and measurements.

Different from usual care
If you decide to participate, you will be randomized and you will receive either haloperidol ór no haloperidol, but will be treated with other drugs to decrease delirium symptoms. These agents other than haloperidol are already used as standard care in the ICU and are also effective against the complaints associated with delirium (such as severe anxiety or delusions or hallucinations - i.e. seeing things that are not there, which is sometimes frightening). The other treatments are the same between both groups. After ICU admission, the follow-up is more extensive compared with usual care because the tests of memory and fitness are not routine. If you do not participate in the study, you will receive routine medication and this is usually the treatment with haloperidol in this hospital.

5. What is expected of you
In order for the research to run smoothly, it is important that you adhere to the following agreements.

The agreements are that you:
- do not participate in any other medical scientific research in which a treatment is tested.
- show up at appointments for follow-up visits.

It is important that you contact the researcher:
- if you no longer wish to participate in the study.
- if your contact details change.

Pregnancy
Women who are pregnant or breastfeeding cannot participate in this study. Treatment with haloperidol can have consequences for an unborn child. This mainly concerns movement disorders, such as muscle stiffness at birth, but it is currently insufficiently known whether haloperidol is entirely safe. A pregnancy test will be performed, so that it can be established with certainty that you are not pregnant and can safely participate in the study.

For peer review only - http://bmjopen.bmj.com/site/about/guidelines.xhtml

6. Possible side effects / complications

Haloperidol may cause side effects / adverse effects.
The *most common disadvantages* of haloperidol:

- slowing of nerve conduction in the heart, which can lead to arrhythmias
- muscle stiffness
- some drowsiness
- mild drop in blood pressure

Rare side effects are:

- muscle breakdown and high fever (so-called “neuroleptic malignant syndrome”)
- serious heart rhythm disorders in which the heart can (temporarily) stop.

The researchers consider the chance of unknown adverse effects / side effects of haloperidol to be virtually nihil, since haloperidol has been used for a long time and all side effects of this drug are well known.

Not receiving haloperidol (placebo) could also have adverse effects.
The possible disadvantages of not giving haloperidol are:

- more restlessness / agitation
- more delusions

When you become restless or suffer from delusions in delirium, other drugs can be given that also work well against anxiety and delusions. The side effects of these other medications are known and usually mild, including slowing of the heart rhythm and drop in blood pressure.

Measurements

The management during admission to the ICU are in accordance with normal practice and do not place an additional burden on you. After discharge, you will be asked to complete questionnaires and tests will be taken during a hospital visit or at home. These tests are not painful.

7. Possible advantages and disadvantages

It is important that you carefully weigh the possible pros and cons before you decide to participate.

Haloperidol can reduce the symptoms of delirium and shorten its duration, and reduce the long-term adverse effects (cognitive complaints and your general functional status), but this is not certain. At any time during treatment with study medication, delirium symptoms may recur or worsen. This does not directly mean that you are in the placebo group, because delirium can also persist despite treatment with haloperidol.

Disadvantages of participating in the study may include:

- Possible side effects of the study medicine (haloperidol) or not receiving the study medicine (placebo)

For peer review only - http://bmjopen.bmj.com/site/about/guidelines.xhtml

- Possible side effects of other medications given during the study period to reduce anxiety and hallucinations
- Possibly confronting questions about your functioning during and after discharge from the hospital.
- Visits to the hospital or home visits, telephone interviews or questionnaires sent can be a burden.

Participation in the study also means:
- That you will have to spend extra time;
- That (extra) tests are done;
- That you have to adhere to agreements for the best result of the research;

All these matters are described above under points 4, 5 and 6.

8. If you do not want to participate or want to quit the study

You should decide for yourself whether you want to participate in the study. Participation is voluntary. If you do not want to participate, you will be treated for delirium in the usual way according to the applicable procedures and protocols. The researcher can tell you more about the treatment options available and their advantages and disadvantages. There is a website with information about the treatment of delirium and the study: https://icudelirium.nl. If you do participate, you can always change your mind and stop, even during the study. You will then be treated for delirium in the usual way. You don't have to say why you quit. However, you must report this immediately to the researcher. The data collected up to that point will be used for the investigation.
If there is new information about the study that is important to you, the researcher will let you know. You will then be asked if you want to continue to participate.

9. End of the investigation

Your participation in the study stops if:
- all visits are over (according to the schedule / as described under point 4)
- you choose to quit
- the researcher thinks it is better for you to quit
- Erasmus MC, the government or the assessing medical ethics review committee decides to stop the research.

The whole study is finished when all participants have been treated and have been followed for the procedures for this study.
After processing all data, the researcher will inform you about the main results of the research. The investigator can also tell you whether you have had haloperidol or the placebo. If you do not want this, you can tell the investigator. The researcher then is not allowed to tell you.

For peer review only - http://bmjopen.bmj.com/site/about/guidelines.xhtml
For peer review only

10. Use and storage of your data

For this research it is necessary that your medical and personal data are collected and used. Each test subject is given a code that will appear on the data. Your name and other personal data that can directly identify you are omitted.

Your data

All your data remains confidential. Only the researchers working in your hospital know which code you have. We will send the data on to the coordinating investigator of the study, but only with that code, never by name. The key for the code remains with the local investigator. Also in reports about the research only that code is used.

Some people are allowed to view your medical and personal data. This is to check whether the research has been carried out properly and reliably. General information can be found in the brochure "Medical scientific research: general information for the test subject". People who can view your medical data are: members of the research team, the safety committee that monitors the investigation, an inspector who works for the client (Erasmus MC), the Health Care Inspectorate. The privacy of your personal data is always maintained. By signing the declaration of consent, you consent to the collection, storage and access of your medical and personal data.

Use of data on a later point

The researcher will keep your data for 15 years. We may use the data to do additional analyses for the study. This concerns research on delirium. You can indicate whether you agree with this on the consent form. You can always withdraw this permission. If no permission is given, you cannot participate in the study.

This research is also included in a public overview of medical scientific research, namely www.trialregister.nl. This website does not contain information that can be traced back to you as a person. However, the website can show a summary of the results. You can find this research under “EuRIDICE trial”. General information about the registration of studies can be found in the brochure "Medical scientific research: general information for the subject".

11. Insurance for test subjects

Everyone participating in this study is insured. The insurance covers damage from the investigation. Not all damage is covered. Appendix B provides more information about the insurance. It also states who can report the damage.

12. Inform GP

We always send your doctor a letter to let them know that you are participating in the study. The details of the tests taken after admission to the Intensive Care Unit are secret. If you wish, or if the researcher deems it necessary, these data can be shared with the GP.

For peer review only - http://bmjopen.bmj.com/site/about/guidelines.xhtml

13. Fee for Participation

The additional tests for the study cost you nothing. You will not be paid for participating in this study. You will receive a compensation of € 22 per visit for your (extra) travel costs and a € 5 lunch allowance per visit. You will not be reimbursed for a home visit.

14. Do you have any questions?

If you have any questions, please contact Dr. M. van der Jagt, principal investigator, or L. Smit, physician researcher. For independent advice on participation in this study, please contact the independent physician Dr. Dinis Dos Reis Miranda, anesthetist intensivist. He knows about this research, but is not involved in its implementation.
It is best to contact the complaints committee of Erasmus MC in case of complaints. All information can be found in Appendix A: Contact details.

15. Signing of consent form

When you have had enough time to consider participation, you will be asked to decide whether to participate in this study. If you give permission, we will ask you to confirm this in writing on the accompanying declaration of consent. By your written permission, you indicate that you have understood the information and that you agree to participate in the study.
The signature sheet is kept by the investigator. You will receive a copy or a second copy of this declaration of consent.

Thank you for your attention.

For peer review only

For peer review only - http://bmjopen.bmj.com/site/about/guidelines.xhtml

16. Annexes to this information

A. Contact details

B. Insurance information

C. Schedule of investigative actions

D. Consent form test subject

E. Brochure "Medical scientific research. General information for the subject (version March 2017) (to be supplied separately)

For peer review only

For peer review only - http://bmjopen.bmj.com/site/about/guidelines.xhtml

Appendix A: Contact details Erasmus Medical Center

Principal Investigator Erasmus Medisch Centrum

Dr. M. van der Jagt, neurologist-intensivist

Via the general telephone number Erasmus MC : 010- 704 07 04

Researcher Erasmus Medisch Centrum

Lisa Smit : physician researcher

Via the general telephone number Erasmus MC : 010- 704 07 04

Or via the research team

Independent physician Erasmus Medisch Centrum

Dr. Dinis Dos Reis Miranda, anesthetist-intensivist

Via the general telephone number Erasmus MC : 010- 704 07 04

Research team Erasmus Medisch Centrum

Ditty van Duijn : Research Coördinator Intensive Care

Patricia Ormskerk : Research Coördinator intensive Care

Alicija Vileito : Research Coördinator Intensive Care

Can be reached during office hours on: : 010-703 51 42

Complaints Committee

Erasmus Medisch Centrum

Can be reached on: : 010-703 31 98

For peer review only

For peer review only - http://bmjopen.bmj.com/site/about/guidelines.xhtml

Appendix B: Information about the insurance

The sponsor insures everyone who participates in this study. The insurance covers damage due to participation in the study. This applies to damage during the investigation or within four years after its end. You must report damage to the insurer within those four years.

The insurance does not cover all damage. At the bottom of this text is briefly mentioned what damage is not covered.
These provisions are set out in the Decree on compulsory insurance for medical research involving human subjects. This decision can be found on www.ccmo.nl, the website of the Central Commission for Human Research (see 'Library' and then 'Laws and regulations').

In the event of damage, you can contact the insurer directly.

The insurer of the study is:	
Name	: CNA Insurance Company Limited
Address	: Strawinskylaan 703
	: 1077 XX Amsterdam
Phone number	: 020 – 573 72 74
E-mail	: Esther.vanherk@cnahardy.com
Polis-number	: HCCD0416C
Contactperson	: Esther van Herk

The insurance covers

- € 650,000 per subject
- € 5,000,000 for the entire study
- € 7,500,000 per insurance year

The insurance does not cover the following damage:

- damage due to a risk about which you have been informed in the written information. This does not apply if the risk is more serious than anticipated or if the risk was very unlikely;
- damage to your health that would have occurred even if you had not participated in the study;
- damage caused by not (fully) following directions or instructions;
- damage to your offspring, as a result of a negative effect of the research on you or your offspring;
- damage caused by an existing treatment method when investigating existing treatment methods.

For peer review only - http://bmjopen.bmj.com/site/about/guidelines.xhtml

Appendix C - Measurements overview

During treatment with the study medication, a heart film will be made daily to determine whether the conduction in the heart is good, and muscle stiffness will be examined. You will also be asked every morning how you slept. In addition, delirium will be assessed daily. The attention is examined and the general condition is examined a few months after the IC admission.

The table below shows which normal care and which extra care you receive in the context of the ICU examination. The table also shows the moments you will be asked to visit the hospital (if desired and feasible for you and us, we will strive for home visits instead of hospital visits) or to complete questionnaires. The tests for memory and thinking take about 1 hour. The table shows the approximate minutes to complete the questionnaires (In brackets).

**Moment (months)**	**Usual care on the ICU**	**Extra care during the study**	**Cognitive tests**	**Experiences related to delirium**	**General condition**
**Once before participation**		- Questionnaire about your memory<br>- Pregnancy test (if applicable)			
**During study at the ICU**	- Delirium assessment (3x/day)	- EKG (1x/day)<br>- Test muscle stifness (1x/day)<br>- Sleep quality (1x/day)			
**0 (discharge hospital)**				*Participant*: Questionnaires (30)<br>*Proxy/family*: Questionnaire (2)	
**1**					Questionnaires (30)
**3**			Hospital or home visit	*Participant*: Questionnaires (35)<br>*Proxy/family*: Questionnaire (12)	Questionnaires (40)
**6**					Questionnaires (30)
**12**			Hospital or home visit		Questionnaires (40)

For peer review only - http://bmjopen.bmj.com/site/about/guidelines.xhtml

For peer review only

For peer review only - http://bmjopen.bmj.com/site/about/guidelines.xhtml

Appendix D: Consent form test subject

Effectiveness of haloperidol for the treatment of acute confusion (delirium) in critically ill patients

- I have read the information letter. I was also able to ask questions. My questions have been answered sufficiently. I had enough time to decide whether to participate.
- I know that participating is voluntary. I also know that I can decide at any time not to participate or to stop the research. I don't have to give a reason for that.
- I give permission to inform my GP that I am participating in this study and to inform them about test results of memory and thinking ability and possible anxiety or depression complaints, if the researcher deems this necessary.
- I know that some people can access my data. Those people are listed in this information letter.
- I consent to the collection and use of my data in the manner and for the purposes stated in the information letter.
- I give permission to keep my data at the research location for 15 years after this research.
- I know I should not be pregnant during the study (if applicable)
- I declare to □ **give**
  □ **not give** permission to contact me again after this investigation for a follow-up investigation
- I □ **do**
  □ **do not** want to be informed about which treatment I had or in which group I was.
- I want to participate in this study.

Name:

Signature: Date: __ / __ / __

For peer review only - http://bmjopen.bmj.com/site/about/guidelines.xhtml

I declare that I have fully informed this subject about the said study.

If during the research information becomes known that could influence the consent of the subject, I will inform him / her in good time.

Name researcher (or its representative):
Signature: Date: __ / __ / __

Additional information is provided by (if applicable):
Name:
Function:
Signature: Date: __ / __ / __

----
* Strike out what does not apply.

The subject will receive a full information letter, along with a copy of the signed consent form.

For peer review only - http://bmjopen.bmj.com/site/about/guidelines.xhtml

Appendix 7: Unblinding Procedure

The study treatment will be unblinded after Database Lock. While the safety of patients should always take priority, maintenance of blinding is crucial to the integrity of a double-blind trial. Before this planned unblinding, the blinding for a specific patient should only be broken when information about the patient's protocol treatment is considered necessary to manage Serious Adverse Events (emergency unblinding). Unblinding procedures should preferably be initiated only after consultation of the principal investigator/coordinating investigator or his/her representative. To initiate an emergency unblinding the pharmacy in charge of the randomisation list should be contacted. Breaking the blinding on a patient will be logged and reported to the coordinating Investigator within 24 hours following the unblinding procedure, using the Emergency Unblinding Form. It is considered a major protocol violation, after which the patient goes off protocol treatment (if applicable).

For peer review only - http://bmjopen.bmj.com/site/about/guidelines.xhtml

Reporting checklist for protocol of a clinical trial.

Based on the SPIRIT guidelines.

Instructions to authors

Complete this checklist by entering the page numbers from your manuscript where readers will find each of the items listed below.

Your article may not currently address all the items on the checklist. Please modify your text to include the missing information. If you are certain that an item does not apply, please write "n/a" and provide a short explanation.

Upload your completed checklist as an extra file when you submit to a journal.

In your methods section, say that you used the SPIRITreporting guidelines, and cite them as:

Chan A-W, Tetzlaff JM, Altman DG, Laupacis A, Gøtzsche PC, Krleža-Jerić K, Hróbjartsson A, Mann H, Dickersin K, Berlin J, Doré C, Parulekar W, Summerskill W, Groves T, Schulz K, Sox H, Rockhold FW, Rennie D, Moher D. SPIRIT 2013 Statement: Defining standard protocol items for clinical trials. Ann Intern Med. 2013;158(3):200-207

		Reporting Item	Page Number
**Administrative information**			
Title	#1	Descriptive title identifying the study design, population, interventions, and, if applicable, trial acronym	1
Trial registration	#2a	Trial identifier and registry name. If not yet registered, name of intended registry	3
Trial registration: data set	#2b	All items from the World Health Organization Trial Registration Data Set	NA
Protocol version	#3	Date and version identifier	2
Funding	#4	Sources and types of financial, material, and other support	22

For peer review only

Roles and responsibilities: contributorship	#5a	Names, affiliations, and roles of protocol contributors	1, 20
Roles and responsibilities: sponsor contact information	#5b	Name and contact information for the trial sponsor	1,2
Roles and responsibilities: sponsor and funder	#5c	Role of study sponsor and funders, if any, in study design; collection, management, analysis, and interpretation of data; writing of the report; and the decision to submit the report for publication, including whether they will have ultimate authority over any of these activities	22
Roles and responsibilities: committees	#5d	Composition, roles, and responsibilities of the coordinating centre, steering committee, endpoint adjudication committee, data management team, and other individuals or groups overseeing the trial, if applicable (see Item 21a for data monitoring committee)	14
**Introduction**			
Background and rationale	#6a	Description of research question and justification for undertaking the trial, including summary of relevant studies (published and unpublished) examining benefits and harms for each intervention	3-5
Background and rationale: choice of comparators	#6b	Explanation for choice of comparators	3-5
Objectives	#7	Specific objectives or hypotheses	4
Trial design	#8	Description of trial design including type of trial (eg, parallel group, crossover, factorial, single group), allocation ratio, and framework (eg, superiority, equivalence, non-inferiority, exploratory)	4
**Methods: Participants,**			

For peer review only

**interventions, and outcomes**			
Study setting	#9	Description of study settings (eg, community clinic, academic hospital) and list of countries where data will be collected. Reference to where list of study sites can be obtained	5
Eligibility criteria	#10	Inclusion and exclusion criteria for participants. If applicable, eligibility criteria for study centres and individuals who will perform the interventions (eg, surgeons, psychotherapists)	5-6
Interventions: description	#11a	Interventions for each group with sufficient detail to allow replication, including how and when they will be administered	9-10
Interventions: modifications	#11b	Criteria for discontinuing or modifying allocated interventions for a given trial participant (eg, drug dose change in response to harms, participant request, or improving / worsening disease)	9-12
Interventions: adherance	#11c	Strategies to improve adherence to intervention protocols, and any procedures for monitoring adherence (eg, drug tablet return; laboratory tests)	11
Interventions: concomitant care	#11d	Relevant concomitant care and interventions that are permitted or prohibited during the trial	11-12
Outcomes	#12	Primary, secondary, and other outcomes, including the specific measurement variable (eg, systolic blood pressure), analysis metric (eg, change from baseline, final value, time to event), method of aggregation (eg, median, proportion), and time point for each outcome. Explanation of the clinical relevance of chosen efficacy and harm outcomes is strongly recommended	7-9
Participant timeline	#13	Time schedule of enrolment, interventions (including any run-ins and washouts), assessments, and visits for participants. A schematic diagram is highly recommended (see Figure)	7-9
Sample size	#14	Estimated number of participants needed to achieve study objectives and how it was determined, including	17

For peer review only

		clinical and statistical assumptions supporting any sample size calculations	
Recruitment	#15	Strategies for achieving adequate participant enrolment to reach target sample size	12
**Methods: Assignment of interventions (for controlled trials)**			
Allocation: sequence generation	#16a	Method of generating the allocation sequence (eg, computer-generated random numbers), and list of any factors for stratification. To reduce predictability of a random sequence, details of any planned restriction (eg, blocking) should be provided in a separate document that is unavailable to those who enrol participants or assign interventions	12-13
Allocation concealment mechanism	#16b	Mechanism of implementing the allocation sequence (eg, central telephone; sequentially numbered, opaque, sealed envelopes), describing any steps to conceal the sequence until interventions are assigned	12-13
Allocation: implementation	#16c	Who will generate the allocation sequence, who will enrol participants, and who will assign participants to interventions	12-13
Blinding (masking)	#17a	Who will be blinded after assignment to interventions (eg, trial participants, care providers, outcome assessors, data analysts), and how	12-13
Blinding (masking): emergency unblinding	#17b	If blinded, circumstances under which unblinding is permissible, and procedure for revealing a participant's allocated intervention during the trial	12-13
**Methods: Data collection, management, and analysis**			
Data collection plan	#18a	Plans for assessment and collection of outcome, baseline, and other trial data, including any related processes to promote data quality (eg, duplicate	7-9

For peer review only

		measurements, training of assessors) and a description of study instruments (eg, questionnaires, laboratory tests) along with their reliability and validity, if known. Reference to where data collection forms can be found, if not in the protocol	
Data collection plan: retention	#18b	Plans to promote participant retention and complete follow-up, including list of any outcome data to be collected for participants who discontinue or deviate from intervention protocols	13-14
Data management	#19	Plans for data entry, coding, security, and storage, including any related processes to promote data quality (eg, double data entry; range checks for data values). Reference to where details of data management procedures can be found, if not in the protocol	12, 15
Statistics: outcomes	#20a	Statistical methods for analysing primary and secondary outcomes. Reference to where other details of the statistical analysis plan can be found, if not in the protocol	16
Statistics: additional analyses	#20b	Methods for any additional analyses (eg, subgroup and adjusted analyses)	16-17
Statistics: analysis population and missing data	#20c	Definition of analysis population relating to protocol non-adherence (eg, as randomised analysis), and any statistical methods to handle missing data (eg, multiple imputation)	14
**Methods: Monitoring**			
Data monitoring: formal committee	#21a	Composition of data monitoring committee (DMC); summary of its role and reporting structure; statement of whether it is independent from the sponsor and competing interests; and reference to where further details about its charter can be found, if not in the protocol. Alternatively, an explanation of why a DMC is not needed	14
Data monitoring: interim analysis	#21b	Description of any interim analyses and stopping guidelines, including who will have access to these	16-17

For peer review only

		interim results and make the final decision to terminate the trial	
Harms	#22	Plans for collecting, assessing, reporting, and managing solicited and spontaneously reported adverse events and other unintended effects of trial interventions or trial conduct	15-16
Auditing	#23	Frequency and procedures for auditing trial conduct, if any, and whether the process will be independent from investigators and the sponsor	NA
**Ethics and dissemination**			
Research ethics approval	#24	Plans for seeking research ethics committee / institutional review board (REC / IRB) approval	17
Protocol amendments	#25	Plans for communicating important protocol modifications (eg, changes to eligibility criteria, outcomes, analyses) to relevant parties (eg, investigators, REC / IRBs, trial participants, trial registries, journals, regulators)	17
Consent or assent	#26a	Who will obtain informed consent or assent from potential trial participants or authorised surrogates, and how (see Item 32)	18
Consent or assent: ancillary studies	#26b	Additional consent provisions for collection and use of participant data and biological specimens in ancillary studies, if applicable	NA
Confidentiality	#27	How personal information about potential and enrolled participants will be collected, shared, and maintained in order to protect confidentiality before, during, and after the trial	18
Declaration of interests	#28	Financial and other competing interests for principal investigators for the overall trial and each study site	22
Data access	#29	Statement of who will have access to the final trial dataset, and disclosure of contractual agreements that limit such access for investigators	-

For peer review only

Ancillary and post trial care	#30	Provisions, if any, for ancillary and post-trial care, and for compensation to those who suffer harm from trial participation	NA
Dissemination policy: trial results	#31a	Plans for investigators and sponsor to communicate trial results to participants, healthcare professionals, the public, and other relevant groups (eg, via publication, reporting in results databases, or other data sharing arrangements), including any publication restrictions	3
Dissemination policy: authorship	#31b	Authorship eligibility guidelines and any intended use of professional writers	-
Dissemination policy: reproducible research	#31c	Plans, if any, for granting public access to the full protocol, participant-level dataset, and statistical code	NA
**Appendices**			
Informed consent materials	#32	Model consent form and other related documentation given to participants and authorised surrogates	-
Biological specimens	#33	Plans for collection, laboratory evaluation, and storage of biological specimens for genetic or molecular analysis in the current trial and for future use in ancillary studies, if applicable	NA

None The SPIRIT checklist is distributed under the terms of the Creative Commons Attribution License CC-BY-ND 3.0. This checklist can be completed online using https://www.goodreports.org/, a tool made by the EQUATOR Network in collaboration with Penelope.ai

For peer review only
